# A mechanistic quantitative systems pharmacology framework enriched by multiscale data enables model-informed translational development of mesenchymal stem cell therapy in lupus nephritis

**DOI:** 10.1016/j.apsb.2026.08.018

**Published:** 2026-08-25

**Authors:** Sujie Zhang, Qixiang Zhang, Dandan Wang, Zichun Li, Xia Xiao, Qi Rao, Siyuan Yang, Yuchen Qu, Yiyang Xu, Ning Wei, Jing Wang, Yuan Gao, Lingyun Sun, Guangji Wang, Fang Zhou, Chen Zhao

**Affiliations:** a School of Pharmacy, Nanjing Medical University, Nanjing 210000, China; b School of Medicine, Yangzhou Polytechnic University, Yangzhou 225000, China; c Key Laboratory of Drug Metabolism and Pharmacokinetics, State Key Laboratory of Natural Medicines, China Pharmaceutical University, Nanjing 210000, China; d Department of Rheumatology and Immunology, The Affiliated Drum Tower Hospital of Nanjing University Medical School, Nanjing 210000, China; e Jiangsu Renocell Biotech Co., Ltd., Nanjing 210000, China; f QSPMed Technologies, Nanjing 210000, China; g Department of Pharmacy, the First Affiliated Hospital with Nanjing Medical University, Nanjing 210000, China

**Keywords:** Quantitative systems pharmacology, Lupus nephritis, Mesenchymal stem cell, Clinical trial simulation, Model-informed drug development, Patient stratification, Virtual patients, Clinical translation

## Abstract

Lupus nephritis (LN) is a complex autoimmune disease and mesenchymal stem cells (MSCs), given their unique immune-modulatory potential, have emerged as a promising route for treating LN. Understanding the multiscale dynamic LN–MSC interactions and selecting the optimal clinical design for MSCs is critical to enhance their therapeutic potential and achieve meaningful benefit-risk differentiation in patients. To guide MSC clinical development, we constructed the first mechanistic quantitative systems pharmacology model of LN pathophysiology that integrated multimodal preclinical–clinical data, with detailed illustration of standard-of-care immunosuppressant pharmacology and physiologically-based biodistribution and therapeutic mechanisms of MSCs. We created model-based virtual LN patients that well captured clinical interpatient heterogeneity and were validated by numerous sets of clinical efficacy readouts, including first-hand results from our investigator-initiated trial testing MSCs in LN patients. We used this computational framework and identified that a spaced two-infusion MSC dosing strategy would yield optimal clinical response in LN patients, which directly informed the phase 2 design of a novel MSC therapy. Modeling analyses further identified that CXCL10 in combination with proteinuria are potential predictive biomarkers that could boost clinical renal response to MSCs. These results demonstrated the exceptional importance of our QSP framework in driving MSC clinical development for LN patients.

## Graphical abstract

A state-of-the-art data-enriched QSP model and virtual clinical trial platform depicting complex LN pathophysiology and MSC bioactivity were built and analyzed to effectively guide the clinical translation of novel MSC-based therapies in lupus nephritis patients.

**Figure:**
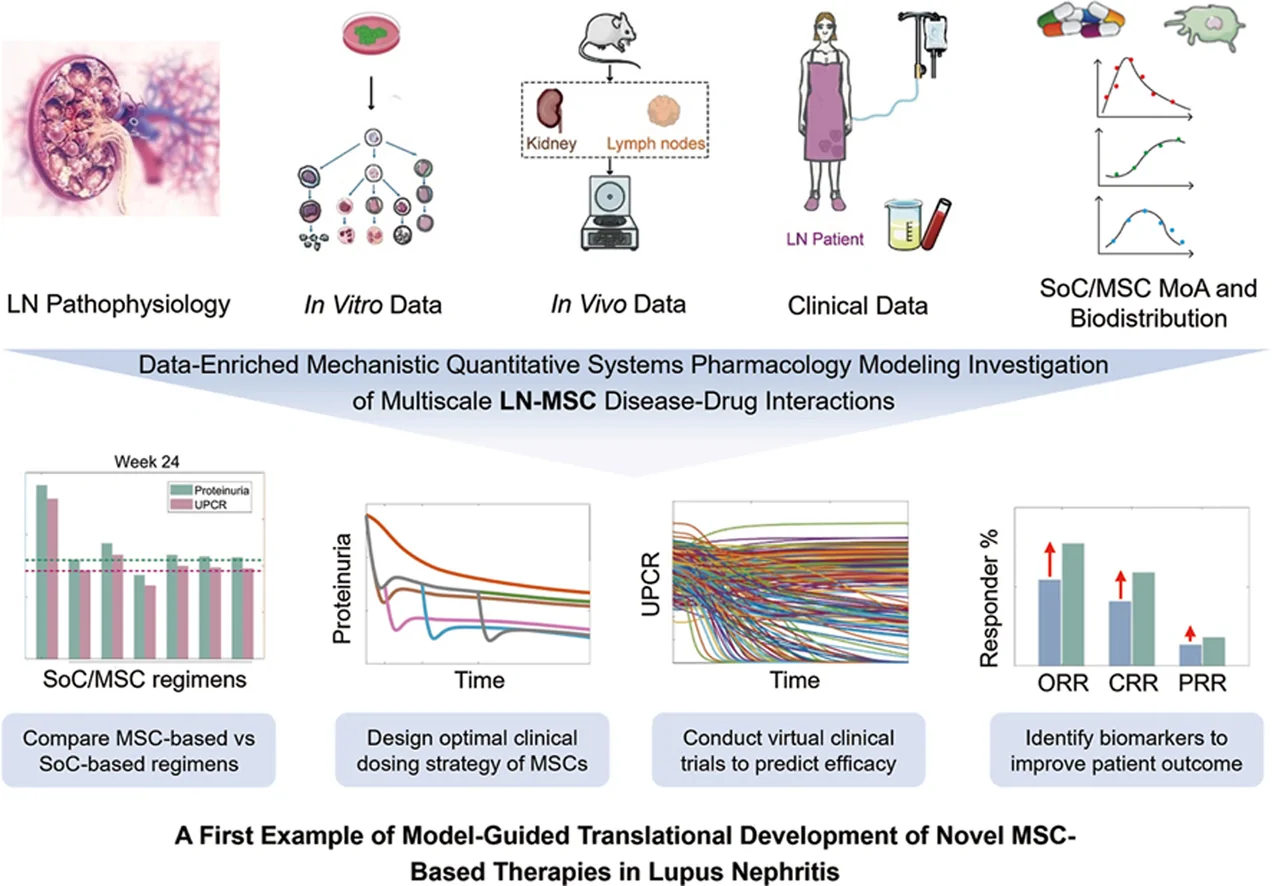

## 1 Introduction

It was reported that the majority of patients (20%–60%) with systemic lupus erythematosus (SLE) can develop lupus nephritis (LN) within five years of their SLE diagnosis, and 5%–20% of those LN patients would progress to end-stage kidney disease^1^^,^^2^. The pathogenesis of LN is highly complex and is influenced by a combination of genetic and environmental factors that collectively delay the timely clearance of apoptotic cells while triggering autoimmunity. In LN, the nuclear materials released from these apoptotic cells are often recognized as autoantigens that can lead to the formation of circulating immune complexes and auto-antibodies. These immune complexes are usually deposited in the glomeruli and can potently trigger the activation of the complement system which aberrantly upregulates immune cell function and inflammatory cytokine activity, and this will ultimately result in tissue inflammation and injury. In a vicious cycle, such tissue damage releases more DNA, nuclear materials and cellular debris, which can further stimulate chronic auto-immune response and exacerbate intrarenal inflammation as well as glomerular fibrosis^3^^,^^4^. For a long time, standard of care therapies for LN primarily include glucocorticoids and immunosuppressant drugs, which are known to have efficacy limitations as well as significant side effects associated with prolonged use^5^. More recently, clinical investigations have explored the use of novel calcineurin inhibitors and anti-B cell biologics for LN which led to the approval of several new therapies. However, challenges still remain in treating LN such as high rebound rates of proteinuria following drug discontinuation, delayed patient response trajectories, potential drug-induced renal toxicity, as well as high treatment expenditures associated with these drugs^6^, ^7^, ^8^. Thus, there is still a large unmet need for the development of new therapies to more effectively control disease progression and alleviate symptoms for LN patients.

Mesenchymal stem cells (MSCs), given their anti-inflammatory properties, are emerging as new therapeutic avenues in a variety of disorders related to alloimmunity and autoimmunity^9^^,^^10^. Mechanistically, MSCs can be conditionally activated by inflammatory environmental factors such as interferon *γ* (IFN-*γ*) and tumor necrosis factor (TNF-*α*), and subsequently they produce large amounts of immuno-modulatory factors and cytokines such as prostaglandin E2 (PGE2), transforming growth factor-beta (TGF-*β*), indoleamine-pyrrole 2,3-dioxygenase (IDO); this process was commonly referred to as “licensing”^11^, ^12^, ^13^. These molecules potently inhibit the proliferation, activation and pathophysiological function of various immune cells including T cells, B cells, plasma cells (PCs) and natural killer (NK) cells, and they also facilitate the induction of regulatory T (Treg) cells. In addition, activated MSCs can promote macrophage polarization toward M2-like immunosuppressive phenotypes to expedite resolution of inflammation^11^^,^^14^, ^15^, ^16^, ^17^. In recent years, increasing efforts have tried to apply MSCs in the clinic, particularly in autoimmune disorders (such as SLE)^18^^,^^19^. However, in LN, whether MSC-based regimens can be novel effective therapies for patients is still in early exploration stages with only one published randomized control trial so far (with a very small sample size). In addition, many technical challenges need to be resolved regarding the clinical trial design of MSCs^17^^,^^20^, such as the optimal MSC clinical dose, dosing strategy/frequency design, choice of efficacy endpoint and measurement methods, as well as the utilization of predictive biomarkers to enhance patient stratification. Considering the escalating cost and time expenditure of clinical trials and the high drug attrition rates (∼90%) during clinical development nowadays, it is critical to develop new translational approaches that can integrate multiscale data and knowledge of LN and MSCs. By prospectively simulating clinical treatment efficacy, such strategies would provide direct guidance for the successful clinical translation of MSC-based therapies in LN in a precision-medicine manner, instead of the traditional trial-and-error methods.

Mechanistic quantitative systems pharmacology (QSP) modeling has emerged in the past decade as a revolutionary concept and powerful research methodology in the modern model-informed drug development paradigm^21^. QSP modeling can integrate diverse multimodal data (preclinical and clinical) into disease-specific physiology-based computational frameworks, which can then enable systems-level quantitative evaluation of the dynamic complex interactions between potential therapeutic strategies and disease pathophysiology as well as the resulting clinical outcomes. Thus, it can provide critical and actionable guidance throughout the multiple stages of the research and development life cycle of innovative therapeutics^22^. To the best of our knowledge, there are no publications or attempts of QSP modeling of LN nor MSC-based treatments in autoimmune disorders; only a few early theoretical modeling studies have tried to characterize certain pathophysiological aspects of LN to aid drug research. Among which, Budu-Grajdeanu et al.^23^ have presented a simplified mathematical model that relates immune-related processes to the qualitative changes of LN disease severity, and another work by Hao et al.^24^ described a theoretical model of the progress from tubulointerstitial inflammation to fibrosis with implications in LN translational research. However, these prior models have not considered the complex multiscale cell-chemokine-cytokine interplay in LN nor the different therapeutic modalities used in clinical treatment (*e*.*g*. MSCs, immunosuppressants, glucocorticoids). In addition, model validation against real-world clinical data is lacking, meaning that there are still large gaps to be filled for model-informed translation research in the field of LN, especially for MSC-based therapies.

Therefore, building on our prior research of MSCs^25^, ^26^, ^27^, here we developed a state-of-the-art multiscale QSP model of LN pathophysiology to effectively guide the clinical translation of novel MSC-based therapies. Our QSP model is the first that mechanistically integrates the diverse mechanisms of LN pathogenesis and treatment (covering a wide range of essential immune cells, cytokines, chemokines, standard-of-care (SoC) drugs, and clinical efficacy biomarkers) as well as the biodistribution and pharmacology of MSCs. The model has undergone rigorous calibration–validation cycles extensively using multimodal datasets including a large number of preclinical experimental data, patient-level measurements and clinical trial readouts. Using this model, we successfully predicted the first-in-human efficacy of an MSC-based regimen implemented in an investigator-initiated trial (IIT) in LN patients, demonstrating its significant utility in LN translational research. We then created model-based virtual patients that incorporated LN patient heterogeneity and comparatively simulated the clinical-level outcomes of different MSC-based therapeutic regimens. Our simulations predicted that a two-infusion (with long interval) clinical dosing strategy of MSCs would achieve optimal efficacy outcomes for LN patients, and this new dosing plan was adopted in the phase II randomized control trial of a first-in-class MSC product (NCT06058078). Moreover, model-based clinical simulations revealed that MSCs can uniquely induce early and long-lasting patient response compared to traditional immunosuppressant regimens alone. Our analyses also showed that increasing MSC doses beyond certain levels may not achieve additional clinical response benefits for patients, and the inclusion of MSCs in clinical treatment can potentially allow dose reduction of immunosuppressant drug use to mitigate potential drug-induced adverse effects. Furthermore, through high-throughput virtual clinical trial analysis, we identified C–X–C motif chemokine 10 (CXCL10) combined with proteinuria or urine protein to creatinine ratio (UPCR) as potential predictive biomarkers that can help stratify LN patients into more advantageous patient subgroups with significantly enhanced clinical response rates to MSC-based regimens. Our data-enhanced mechanistic QSP framework presented here is an illuminating original attempt of model-informed drug development in LN and it has offered crucial guidance for the clinical translation of a novel MSC-based therapy for LN patients.

## 2 Materials and methods

### 2.1 Model formulation and in silico pathophysiological simulations using virtual patients and virtual clinical trials

Overview of QSP model components and interactions are illustrated in Fig. 1. Detailed descriptions of the biological rationale, molecular components and exemplar equations comprising the model are presented in the Supporting Information The QSP model was developed using the Simbiology Toolbox in MATLAB (MathWorks, Natick, MA, USA). The model was constructed based on ordinary differential equations (ODEs) using mass-action laws and Hill-type functions with about 80 molecular species in the system (including ∼30 species representing MSC and SoC PK components). Three clinically approved SoC therapeutics (MMF, CTX, PDN) were incorporated and their PK profiles were characterized using standard compartment models^28^, ^29^, ^30^, ^31^. To characterize the concentrations of these drugs entering the tissues, we assumed that drug exposures in the tissue are proportional to that in the blood by multiplying partition coefficients. For step-wise model calibration, at the cellular level we primarily used experimental data from *in vitro* cultures, including T cells and their subpopulations, B cells and their subpopulations, macrophages and podocytes. Then, a collection of multiscale data including the degradation and secretion rates of cytokines, antibodies, and complement proteins by various immune cells, the levels of cytokines and cells in animal and human tissues, and clinical levels of disease-related biomarkers were curated and incorporated as the basis for scoping and modelling of a healthy individual and a reference LN patient. We then added the mechanisms of action and targets of different SoC drugs and matched the reference LN virtual patient’s baseline biomarker characteristics according to literature. Notably, this reference LN virtual patient was calibrated to multiple clinical trials simultaneously regarding post-treatment response, encompassing different doses, tapering cycles and drug combinations (of MMF, CTX and prednisone). For MSCs, we developed a PBPK model by assuming fast processes of MSC transportation into various organs with a portion of MSCs being arrested and extravasated into organ tissue. All physiological parameters in the PBPK model (body weight, organ volume, blood volume and blood flow, etc.) were set according to the literature. LN-specific parameters relating to the MSC lifecycle were optimized against relevant preclinical and clinical data. In this sense, the LN disease module, MSC biodistribution module and SoC module were initially built and calibrated independently, and were then later combined together when simulating the patient-level response to different drug treatments.

**Figure 1:**
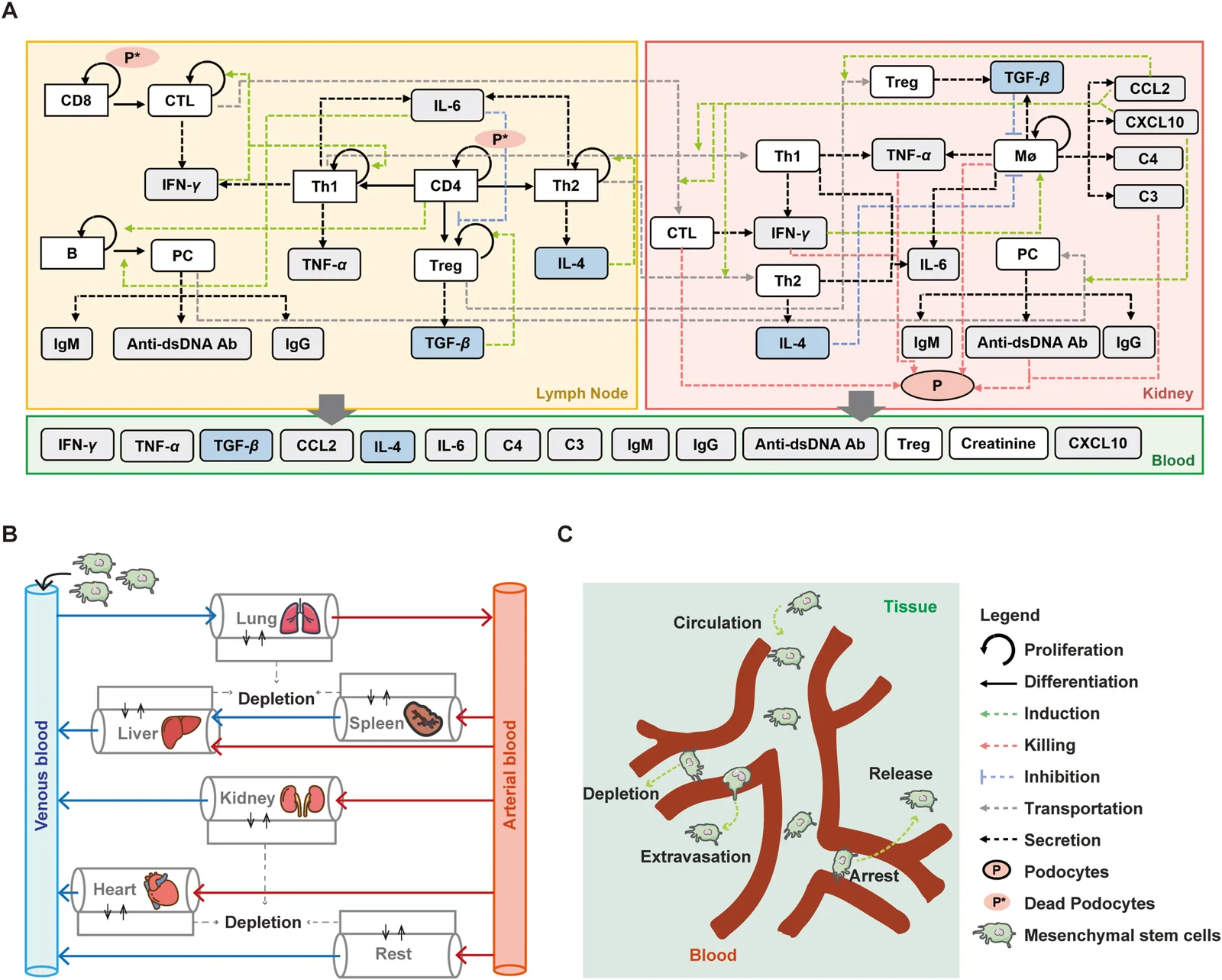
Overview the integrated LN QSP model framework. Within the QSP model. (A) the lupus nephritis disease module captures the complex interaction between numerous immune cells, regulatory biomolecules and disease outcome markers across tissue compartments including the lymph nodes, kidneys and peripheral blood. (B, C) In the MSC biodistribution module, MSCs travel with the circulatory system to different body organs, where they get passively arrested in the organs and they also extravasate to the tissue components within organs. Over time, MSCs are removed from the body. More details regarding the QSP model and its modules are described in Methods and Protocol S1. Abbreviations: B, B cell, PC, plasma cell, P, podocyte, M*Ф*, macrophage, Treg, regulatory T cells, CD4, CD4^+^ T cell, CD8, CD8^+^ T cell, CTL, cytotoxic T lymphocyte, Th1, T helper 1 cell, Th2, T helper 2 cell.

To further account for the interindividual variability that can result in heterogeneous patient response to different therapeutic treatments, we used the virtual patient population (Vpop) approach to match the population-level patient baseline characteristics and response features to different therapies. Initially, parameter distribution statistics (*e*.*g*., mean, standard deviation) were estimated for a set of model parameters assumed to vary between patients, and we sampled this resulting parameter space with each parameterization representing a potential virtual patient. These virtual patients would undergo multilevel selection processes to form a preliminary pool of plausible patients. Then, clinically-reported population-level response readouts such as percentages of patients achieving overall renal response/complete renal response/partial renal response were used to generate the optimal probability distribution to derive the final virtual patient population (Vpop). During biomarker screening analyses for MSCs, we took the top 25% and bottom 25% of patients according to their biomarker response and statistically assessed the comprehensive distributional differences of their pretreatment biomarkers to derive the candidate biomarkers for further testing. Our model-based Vpop was then categorized into different subgroups based on exhaustive pre-treatment thresholding of the candidate biomarkers (as well as their combinations), and absolute ORR percentages were simulated and calculated following clinical dosing protocols for each subgroup after SoC and MSC-based regimens to rank the biomarkers with the greatest potential for patient stratification. The process and details of model formulation, optimization, validation and simulations (including virtual clinical trials) are described in more details in the Supporting Information Protocols S1 and S2, and Tables S1–S8.

### 2.2 Global sensitivity analyses

For global sensitivity analyses, the partial rank correlation coefficient (PRCC) algorithm was used according to the methodology introduced by Marino et al.^32^. The resulting proteinuria value at Week 24 was the output of interest for all PRCC calculations. Two different treatment conditions were analyzed, including MMF plus prednisone, and MMF plus prednisone combined with MSCs. The exact dosages simulated are given as follows: (1) oral MMF given twice daily, titrated from 0.5 g twice daily in Week 1 and 1.0 g twice daily in Week 2, to a target dosage of 1.5 g twice daily in Week 3 and beyond, and oral prednisone is tapered from a maximum starting dosage of 60 mg per day; (2) oral MMF twice daily for 24 weeks at a total dose of 2 g per day, and oral prednisone starting at 60 mg per day for 4 to 6 weeks, then decreasing dose over a period of 3 to 4 months until a maintenance dose of between 5 and 10 mg per day, and MSCs (1 × 10^8^ cells each infusion) were administered intravenously two times at a one-week interval.

### 2.3 Mice

Female MRL/lpr and MRL-MpJ mice aged 6 to 8 weeks were purchased from Shanghai SLAC Laboratory Animal Co (Shanghai, China). Following the Animal Care and Use Committee guidelines, all mice were housed in a pathogen-free facility. All animal experiments were approved by Animal Ethics Committee of China Pharmaceutical University (Animal Authorization Reference Number: 2021-06-023).

### 2.4 hUC-MSCs preparation and dosage

hUC-MSCs were injected intravenously into mice with the product code ‘RY_SW01 Cell Injection’ manufactured by Jiangsu Renocell Biotech Co., Ltd (Nanjing, China). The cell product has been certified by the National Institutes for Food and Drug Control of China. The product consists of a cell suspension, and the cell activity remains stable for 48 h at 2–8 °C. Mice were given 200 µL hUC-MSCs (8 × 10^5^ cells each mouse) through the tail vein.

### 2.5 Detection of hUC MSCs

Detection of hUC-MSCs in tissues was performed using the qPCR method based on the Alu gene. Primers for quantitative polymerase chain reaction (PCR) were designed to target the human-specific unique sequence of Alu, utilizing a primer set specific for human Alu repeats^26^. Total genomic DNA was extracted from mouse tissue using the FastPure Cell/Tissue DNA Isolation Mini Kit (Vazyme, China), following the manufacturer's instructions. qPCR assay of gDNA was performed with SYBR Green Mix (Bio-Rad, USA). The standard curve of cycle threshold values (Ct) *versus* cell number (logarithmic form) was obtained through qPCR amplification. Each test tissue was analyzed alongside the corresponding standard curves, allowing for the calculation of hUC-MSCs per unit weight based on the standard curve. The precision and accuracy of this method have been validated for preclinical evaluation.

### 2.6 Imaging of RFP-MSCs in the kidney

hUC-MSCs were transfected *in vitro* using lentivirus (Jiman Biotechnology, Shanghai, China) to achieve stable expression of RFP fluorescent protein. Mice were given 200 µL RFP-hUC-MSCs (8 × 10^5^ cells/mice) through the tail vein. At the 1 day after injection, mice were anesthetized with isoflurane and sacrificed, kidneys were collection. Kidneys were fixed in 4% paraformaldehyde for 24 h, followed by sequential overnight dehydration with 20% sucrose and 30% sucrose in PBS. Tissue slides were washed with PBS, then stained with DAPI, and finally mounted. Images were observed using an Olympus Fluoview FV3000 confocal microscope (Olympus, Tokyo, Japan).

### 2.7 Immunofluorescence

Kidneys were fixed in 4% paraformaldehyde for 24 h, followed by sequential overnight dehydration with 20% sucrose and 30% sucrose in PBS. Kidney slides were blocked at room temperature for 1 h with 0.1% Triton X-100 in PBS and 5% donkey serum. They were then incubated overnight at 4 °C with anti-mouse CD4 antibody (1:500, Abcam). Alexa Fluor 594 anti-rabbit secondary antibodies were applied, washed with PBS, then stained with DAPI, and finally mounted. Images were observed using an Olympus Fluoview FV3000 confocal microscope (Olympus, Tokyo, Japan).

### 2.8 In situ hybridization

To assess fluorescent *CXCL1*0 mRNA expression in the glomeruli of MRL/lpr and MRL/MpJ mice, we conducted an *in situ* hybridization procedure. The mouse CXCL10 monoclonal factor probe for this process was synthesized by Bio-Techne (Minneapolis, MN, USA), with supporting reagents also supplied by Bio-Techne. Frozen kidney tissue sections after fixation were assayed in combination according to the manufacturer’s instructions. Images were then observed using an Olympus Fluoview FV3000 confocal microscope (Olympus, Tokyo, Japan).

### 2.9 Preparation of kidney homogenate

Mice were euthanized to obtain fresh kidneys. Each kidney was added to 2 mL of serum-free DMEM/F12 (Gibco, Grand Island, NY, USA) cell culture medium and homogenized using a glass homogenizer placed on ice. After homogenization, the homogenate was removed and centrifuged at 1000 ×*g* for 10 min at 4 °C. The supernatant was collected and filtered through a 0.22 μm five-strain filter in a five-strain biosafety cabinet. The collected supernatant was stored at −80 °C for later use.

### 2.10 T cell induced to Th1 cells and co-culture with MSC

MSCs were pre-inoculated on transwell inserts in 6-well plates. Naive CD4^+^ T cells were isolated from the spleens of mice. Naive CD4^+^ T cells were cultured in a 6-well plate which was pre-coated with 2 µg/mL anti-CD3. Th1 cells were induced using differentiation medium supplemented with IL-12 (20 ng/mL; PeproTech), IL-2 (20 ng/mL; PeproTech), anti-IL-4 antibody (10 µg/mL; PeproTech), and anti-CD28 antibody (5 µg/mL; PeproTech). After three days of culture and differentiation, MSCs implanted in transwell inserts were co-cultured with Th1 for two days. After 2 days of co-culture, cells were collected for Flow Cytometry.

### 2.11 PBMC extraction and co-culture with MSCs

PBMCs were extracted from fresh human blood samples by density gradient centrifugation, and were cultured in 1640 medium containing 10% calf serum (Gibco). Then, MSCs implanted in transwell inserts were co-cultured with PBMCs for 3 days. After three days of co-culture, MSCs were collected for q-PCR.

### 2.12 Enzyme-linked immunosorbent assay (ELISA)

Enzyme-linked immunosorbent assay (ELISA) kits for protein levels were as follows: Mouse IFN-*γ*, TNF-*α* are all from Wuhan Huamei (Wuhan, China), Mouse anti-dsDNA antibody is from FUJIFILM Wako Shibayagi (Shibukawa, Japan), TGF-*β*1 is from Elabscience Biotechnology Co., Ltd. (Wuhan, China). All experiments were conducted according to the manufacturer's instructions.

### 2.13 Flow cytometry

MSCs were harvested and stained with an anti-human CXCR3 antibody for subsequent analysis. Th1 cells were identified by intracellular staining of the transcription factor T-bet following fixation and permeabilization using a transcription factor staining buffer set (BD Biosciences). Flow cytometric analysis was performed on a CytoFLEX S flow cytometer (Beckman Coulter, Brea, CA, USA), and the resulting data were analyzed using FlowJo software (FlowJo, Ashland, OR, USA). All antibodies used for Flow cytometry were obtained from BioLegend (L/D 423,102, anti-human CXCR3 126,505, anti-mouse CD45 103,105, anti-mouse CD4 100,406, anti-mouse Tbet 644,823).

### 2.14 mRNA extraction and qRT-PCR amplification

Total mRNA was extracted with RNAiso Plus (Takara, Japan) based on the manufacturer’s protocol, the concentration and purity were detected by the Colibri Spectrophotometer (Berger, Germany). Then, total mRNA was converted to complementary DNA by HiScript Ⅲ RT SuperMix (Vazyme, China). The Bio-Rad C1000 server system (Bio-Rad, Hercules, USA) was used to determine mRNA levels. The 2^−ΔΔCt^ method was utilized for quantitative analysis. The mRNA levels were calculated with GAPDH as reference genes. All the primers were synthesized by Sangon Biotech Co., Ltd (Shanghai, China). Primer sequences are shown in Table 1.

**Table 1:** Primer sequences used in PCR analysis.

Gene name	Forward (5ʹ–3ʹ)	Reverse (5ʹ–3ʹ)
GAPDH_Homo	CATGTAGGCCATGAGGTCCACCAC	TGAAGGTCGGTGTCAACGGATTTGGC
TGFB1_Homo	GGCCAGATCCTGTCCAAGC	GTGGGTTTCCACCATTAGCAC
CXCR3_Homo	CCACCTAGCTGTAGCAGACAC	AGGGCTCCTGCGTAGAAGTT

### 2.15 Patient selection and treatment protocol of the investigator-initiated trial

Five active LN patients were recruited from the Affiliated Drum Tower Hospital of Nanjing University Medical School (Nanjing, China). All the patients aged 22–49 years were diagnosed as LN with a SLEDAI-2K score of more than eight and renal biopsy of WHO class III, IV, V, III+V or IV + V. All the patients were treated with allogenic hUC-MSCs transplantation (2 × 10^6^ cells/kg per patient, one infusion) and provided informed consents for the collection of peripheral blood. The immunosuppressants were withdrawn one week before MSC infusion; concomitant daily low-dose prednisone was allowed for patients. This study was approved by the Ethics Committee of The Affiliated Drum Tower Hospital of Nanjing University Medical School (No. SC-2017-002) and registered on ClinicalTrials.gov (Identifier: NCT01741857).

### 2.16 Statistical analyses

An *R*-square (*R*^2^) value was calculated to assess the goodness-of-fit between model-predicted ORR percentages and clinical observation data under different drug dosing regimens. *t*-Tests and Wilcoxon rank-sum tests were conducted using ttest 2 function and rank sum function in MATLAB respectively during model-simulated biomarker evaluations. Unpaired *t*-tests were used for experimental data analysis. *P* values < 0.05 were considered statistically significant.

## 3 Results

### 3.1 Formulation of the integrated LN QSP model framework

The integrated QSP model framework is comprised of three major components: the LN disease module, MSC *in vivo* biodistribution module, and SoC therapeutics module. The LN disease module consists of three main body compartments (lymph nodes, kidneys, and peripheral blood) and was primarily formulated based on established immune-related mechanisms of LN pathogenesis, including the complex interplay between a number of major immune cell players such as macrophages, B cells, T cells (various subtypes) and plasma cells as well as a number of immuno-modulatory cytokines and chemokines (Fig. 1A). In short, processes of lymphocyte proliferation and differentiation take place in the lymph nodes, and this is driven by excessive anti-self killing of renal cells (podocytes) in the kidneys and resulting antigen presentation and immune activation. The various subtypes of B and T cells residing in the lymph nodes can interact extensively with each other through immune cytokines (IL-4, IL-6, TNF-*α*, IFN-*γ*, TGF-*β*, etc.). The kidneys are the main sites where effector lymphocytes (B, T and plasma cells) exert their regulatory function such as cytokine production, auto-antibody production and killing of renal cells (podocytes)^33^^,^^34^. The killing of podocytes is used as a representation of renal tissue damage and it is collectively driven by cytotoxic T lymphocytes (CTL), macrophages, autoantibodies, and a number of pro-inflammatory/pro-apoptotic cytokines^35^, ^36^, ^37^. In turn, the cytokines and chemokines (*e*.*g*., CCL2–C–C motif chemokine ligand 2, and CXCL10) produced in the kidney tissue microenvironment by the immune cells would further recruit more immune effector cells into the inflamed renal tissues, thereby exacerbating tissue damage^38^, ^39^, ^40^, ^41^. These biomolecules in the renal tissue can also dynamically regulate the activational states of the various residing immune cells. Ultimately, the extent of renal cell killing is quantitatively linked to LN outcome measures such as 24 h proteinuria and UPCR, and the expression of various biomolecules and cells in the lymph nodes and inflamed kidneys would directly contribute to the corresponding 10+ peripheral observational markers in LN patients. Additional details regarding the LN disease module are detailed in the Methods section and Supporting Information Protocol S1.

For the MSC biodistribution module, we developed a physiologically-based pharmacokinetic (PBPK) model to assess MSC *in vivo* biodistribution and persistence. The PBPK model divided the entire body into eight compartments: arterial blood, venous blood, lungs, spleen, liver, kidneys, heart, and the rest of the body, similar to a prior work (Fig. 1B)^42^. All compartments were interconnected *via* the systemic blood circulation. After intravenous injection, MSCs were transported to different organs *via* blood flow, and after reaching the tissue components of the organs, some MSCs can get trapped (arrested) in the tissue capillaries due to their relatively large size or their adherence to the endothelial wall. A portion of these trapped MSCs can be dynamically released back into the organ vascular components and then back to the systemic circulation, or they can also get eliminated *via* cell death and removal (depletion) (Fig. 1C). In this way, a portion of infused MSCs would dynamically travel to and reside in the kidneys, and over time they will get removed. For the SoC therapeutics module, mycophenolate mofetil (MMF), cyclophosphamide (CTX) and prednisone (PDN) were considered. The *in vivo* metabolism and disposition of these SoC drugs were described by one-compartment or two-compartment pharmacokinetic models (Protocol S1).

Different pharmacology and mechanisms of action were considered and implemented in the QSP model for MSCs and different SoC therapeutics. We incorporated the following established mechanisms of MSCs, including “licensing” of MSCs triggered by inflammatory cytokines, enhanced migration of MSCs induced by chemokines, secretion of various immuno-modulatory cytokines by MSCs, regulation of immune cells’ cytokine-secreting capacity by MSCs, and Treg differentiation induced by MSCs^11^^,^^43^. For CTX, it is modeled as an inactive prodrug that can be metabolized to 4-hydroxycyclophosphamide (4-OHCTX) that can then exert direct cytotoxic effects on immune cells^44^^,^^45^. MMF is the modeled as the prodrug of mycophenolic acid (MPA), and the metabolite MPA can downregulate lymphocyte proliferation and inhibit antibody production^46^, ^47^, ^48^. The major class of glucocorticoid implemented in the model is prednisone, and it can be metabolized to form prednisolone (Pred), which can promote apoptosis of activated T cells^49^, inhibit antibody production by plasma cells^50^, and suppresses the cytokine secretion^51^, ^52^, ^53^, thereby alleviating tissue damage and inflammation. In addition, methylprednisolone is also included as a SoC therapeutics with a simplified conversion step. More details on the model implementation and representative model equations, rules, and reactions can be found in Protocol S1.

### 3.2 QSP model-based development and calibration of a reference LN virtual patient

After we designed the high-level mechanistic QSP model structure, we then incorporated a large number of multiscale experimental data in a step-wise manner to inform the parameterization and calibration of the QSP model, from the *in vitro* level all the way to the clinical LN patient level (Fig. 2A). We started with the cell-level proliferation and differentiation processes by incorporating time-course cell number data obtained for podocytes, naive CD4^+^ and CD8^+^ T cells, naive B cells, macrophages (M*ø*) as well as their effector subpopulations including T-helper 1 cells (Th1), T-helper 2 cells (Th2), Tregs, CTLs, and plasma cells (PCs) (Supporting Information Figs. S1 and S2). In addition, a large number of curated cytokine release rates as well as different cell and cytokine expression levels in tissues derived from *in vitro* and *in vivo* measurements were used together and calibrated during model parameterization (Tables S1 and S2). After iterative optimization of the LN disease module, at the clinical level, we are able to simulate the quantitative expression as well as up- and down-regulations of the various cells and biomolecules in the lymph nodes and renal tissues in a healthy adult *versus* in a diseased LN patient (Fig. 2B and C). The overall trends derived from the individual LN patient simulations suggested that the majority of pro-inflammatory mediators were upregulated in LN tissues while immuno-suppressive mediators (*e*.*g*., Treg, TGF-*β*) were downregulated, and this is consistent with a number of literature reports as well as our findings from an experimental MRL/lpr mouse model of lupus nephritis revealing that inflammatory mediators such as CD4^+^ T cells, CXCL10, IFN-*γ* and TNF-*α* were highly expressed in the kidneys of MRL/lpr mice compared to normal controls (Fig. 2D and E, and Supporting Information Fig. S3A–S3C). We then enriched our model-based reference LN patient by calibrating its efficacy-related clinical endpoints (*e*.*g*., 24 h proteinuria, UPCR) and circulating biomarkers in blood (creatinine, total IgG/IgM, anti-dsDNA Ab, C3, C4, IL-4, IL-6, TNF-*α*, IFN-γ, TGF-*β*, CCL2, CXCL10 and Treg%) against corresponding clinical data, and overall our model has achieved a high-level quantitative consistency with the reported clinical measurements (Fig. 2F and G, and Table S3). The same methodology was also applied to assess the accuracy of simulated efficacy endpoints and circulating biomarkers in the reference healthy subject, and the results again match well with published clinical data obtained from healthy adults (Fig. 2F), which further supports the validity and utility of our model-based virtual patient strategy from the perspective of LN disease pathophysiology.

**Figure 2:**
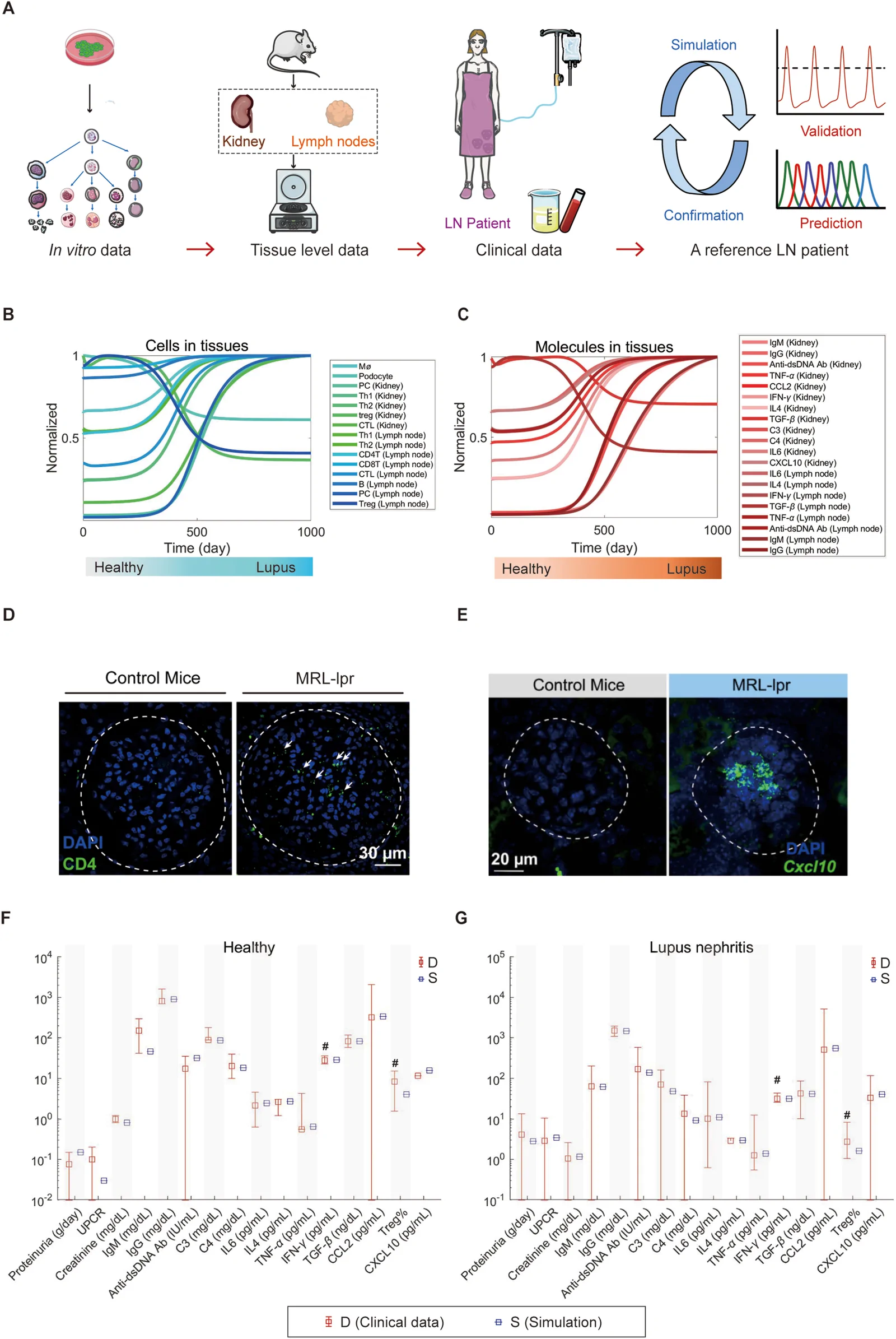
Formulation and calibration of the LN disease module in the QSP model. (A) Workflow of module formulation and utilization of multiscale experimental data toward the development of model-based reference LN virtual patient. (B, C) Relative changes in the expression of various immune cells and regulatory biomolecules in tissues of a healthy volunteer compared to a LN patient as simulated by the QSP model (*y*-axes are normalized relative expression levels). (D, E) Comparison of fluorescent CD4^+^ T cell amounts and *Cxcl1*0 mRNA expression in the glomeruli of control and MRL/lpr (lupus nephritis) mice. Scale bar: 30 μm and 20 μm. (F, G) Comparison of model simulated values (blue) and clinically reported ranges of baseline biomarker levels (red, in medians plus ranges according to literature) for a reference health volunteer and LN virtual patient. S, model simulation; D, clinical data. Abbreviations: B, B cell, PC, plasma cell, P, podocyte, M*Ф*, macrophage, Treg, regulatory T cells, CD4, CD4^+^ T cell, CD8, CD8^+^ T cell, CTL, cytotoxic T lymphocyte, Th1, T helper 1 cell, Th2, T helper 2 cell.

### 3.3 Physiological model-based characterization of *in vivo* MSC biodistribution and validation by multiscale experimental data

A PBPK module was built to characterize the physiological *in vivo* cellular kinetics of administered MSCs in order to enable cross-species quantitative prediction of *in vivo* MSC cell fate (Fig. 3A, and Tables S4 and S5). Using this 8-compartment PBPK framework, we successfully captured the differential time-course dynamics of MSCs in major organs under different doses in mice as reported in literature (Fig. 3B^42^, and Supporting Information Fig. S4A and S4B^55^^,^^56^). Then, we adjusted species-specific physiological parameters including body weight, organ volume and blood flow according to corresponding numbers of rats, and we quantitatively validated the model's predictive capacity as it again accurately depicts the reported *in vivo* concentration-time patterns of MSCs measured in rats (Fig. 3C)^54^. In addition, we explored the predictive utility of this PBPK framework in diseased animals (experimental LN mice), and by adjusting only a few disease-related physiological parameters, the model can recapitulate the temporal dynamics of MSCs in LN mice (Fig. 4A)^57^. To further validate our PBPK framework, we administered MSCs (8 × 10^5^ cells per mouse) in control as well as in MRL/lpr mice and measured the distribution of MSCs in different organs. Our mouse data showed significantly higher and more persistent renal homing and less lung homing of MSCs in MRL/lpr mice compared to controls, which is consistent with our model simulations suggesting that MSCs may be preferentially attracted to inflamed tissues in LN disease settings (Fig. 4B and C, and Fig. S4F).

**Figure 3:**
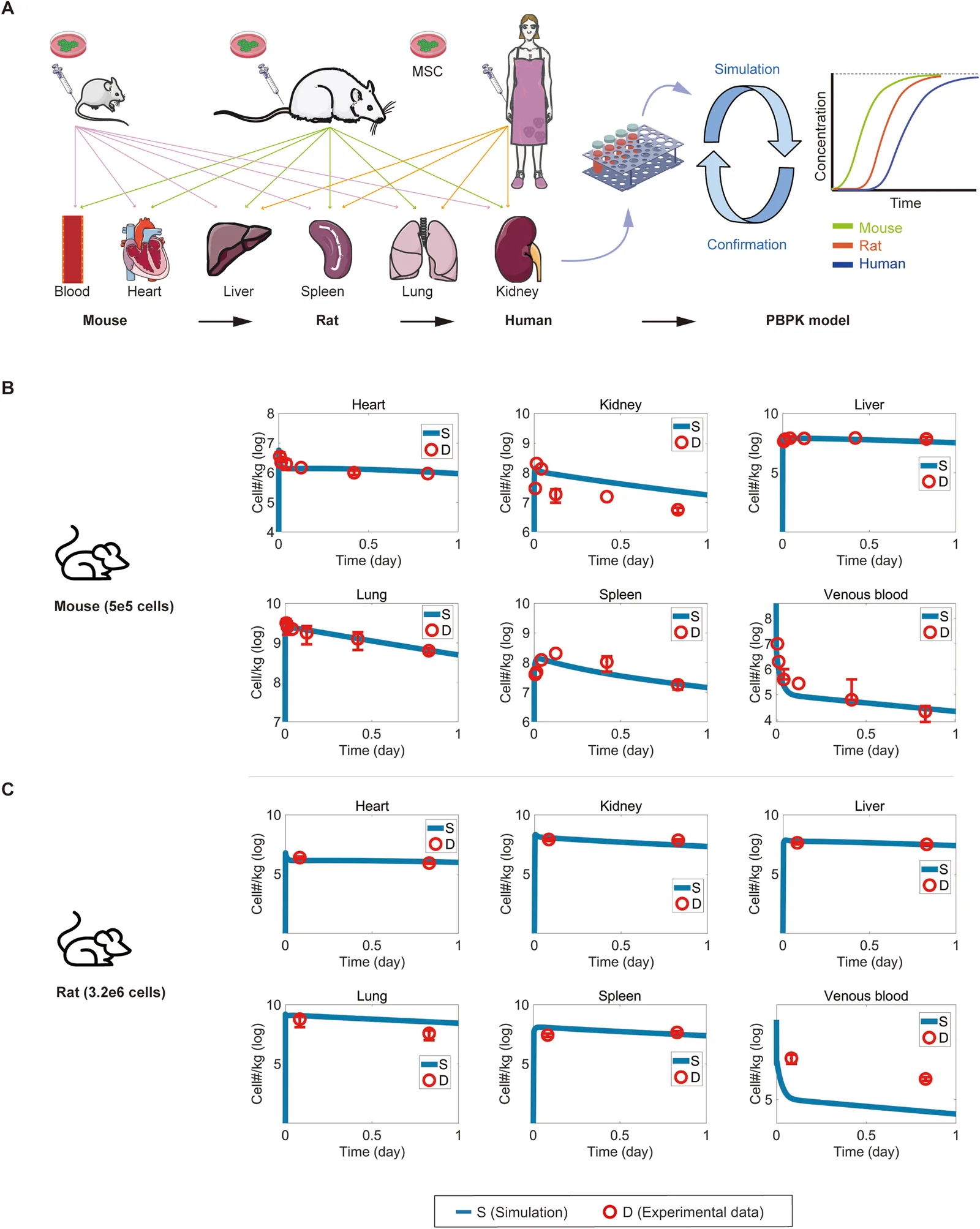
Physiologically-based description of MSC biodistribution *in vivo* by the modeling framework and comparison with experimental data. (A) Workflow describing the cross-species formulation and calibration of the MSC biodistribution PBPK model. (B, C) Model simulations of dynamic MSC biodistribution in the different organs in rodents upon intravenous MSC dosing and direct comparison with corresponding experimental data, including short-term measurements from the heart, kidneys, liver, lungs, spleen and venous blood of (B) BALB/c mice (5 × 10^5^ cells per animal, as reported in Ref. 42) and (C) Sprague–Dawley rats (3.2 × 10^6^ cells per animal, as reported in Ref. 54). MSC concentrations in the different organs were expressed in the unit of absolute cell number per kilogram of organ tissue (on a log 10 scale). S, model simulation; D, experimental data.

**Figure 4:**
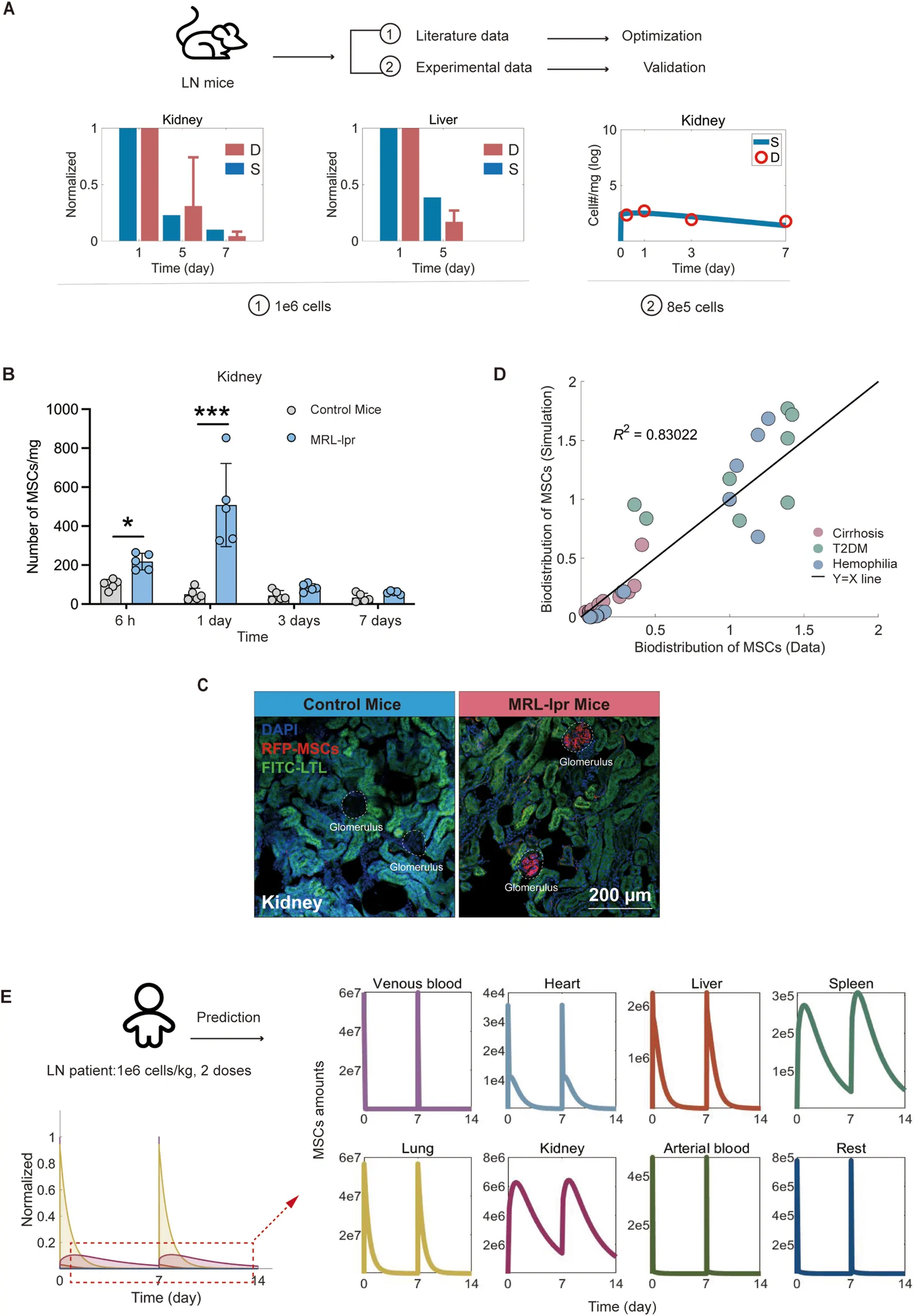
Enhanced MSC migration observed in LN disease settings drives model-based dynamic characterization of MSC biodistribution at the clinical level. (A) The PBPK module was first calibrated by MSC biodistribution data (values are normalized to Day 1 values) measured in MRL/lpr mice (1 × 10^6^ cells per animal i.v. as reported in Ref. 57), and it was then validated against additional in-house data measuring hUC-MSC levels in the kidneys of MRL/lpr mice (dosed with 8 × 10^5^ MSCs per animal i.v.). In the validation set, model simulations and experimental data are in the unit of cell number/mg of kidney tissue (on log 10 scale). (B) Abundance of hUC-MSCs in the kidneys of diseased mice (MRL/lpr) as detected by qPCR during a course of 7 days are considerably higher and more persistent than that in the control mice (*n* = 5). ∗*P* < 0.05 and ∗∗∗*P* < 0.001, respectively; data are expressed as mean ± SD. (C) Strong expression of RFP fluorescent protein in kidneys of MRL/lpr mice injected with RFP-hUC-MSCs (8 × 10^5^ cells/mouse), indicating significantly enhanced presence of MSCs in the kidneys of diseased mice *versus* in control mice. Scale bar: 200 µm. (D) The PBPK module has achieved an overall high goodness-of-fit between clinically-measured MSC biodistribution data *versus* corresponding model predictions at the patient-level (normalized data from 4 trials measuring biodistribution in human using radiolabeling were summarized here). Details regarding comparisons of quantitative simulation results *versus* clinical data are provided in Fig. S4C–S4E. (E) Model-simulated quantitative profiles of MSC biodistribution (in cell numbers) in the different organs in a reference LN patient who received two repeated i.v. MSC injections (1 × 10^6^ cells/kg per injection, at 1-week interval). S, model simulation; D, experimental data.

Acquiring MSC biodistribution data at the human level is a very challenging task as the only way to trace MSCs in human is through radiolabeling. Thus, the aforementioned validated PBPK framework can provide important clinical-level predictions of MSC *in vivo* cell fate *via* cross-species extrapolation. This translational idea was first tested against a dataset comprising published human MSC biodistribution measurements (obtained *via* radiolabeling, patients diagnosed with different diseases other than LN), and we found that the simulation results, upon minor model parameter adjustments, can well characterize (*R*^2^ > 0.83) the general spatiotemporal disposition of MSCs in real human patients (Fig. 4D, and Fig. S4C–S4E). With this additional layer of validation, we then used the PBPK framework to infer the MSC biodistribution in lupus nephritis patients after intravenous MSC dosing. For the exemplar scenario of two MSC doses (1 × 10^6^ cells/kg each dose, one week interval) in LN patients, our simulations suggested that the time-course concentration profiles of MSCs can differ significantly between the various human organs in LN patients, with particularly enhanced MSC persistence in the kidneys and spleen compared to other organs (Fig. 4E). According to our translational simulations, MSCs would get rapidly re-distributed from the blood to organs in human after intravenous injection, with the lungs retaining the highest amount of cells (similar to rodents); in the meantime, it seems that the majority of MSCs would get cleared within 1–2 weeks in most organs.

### 3.4 Comprehensive calibration and validation of the model-based reference LN virtual patient using clinical treatment response data

In an iterative calibration-validation manner, we then aim to further strengthen the predictive capacity of our reference LN virtual patient in terms of simulating post-treatment efficacy response, for which we have incorporated 10+ published trials of LN that have used different SoC treatment regimens (covering MMF, glucocorticoids and CTX) (Fig. 5A). Among them, 15 trials testing low dose SoC treatment regimens were selected as the calibration set and 4 trials testing higher doses of SoC were selected as the validation set. Clinical-level pharmacokinetic (PK) profiles of the SoC drugs under different dosing regimens were described by compartmental models as mentioned before and were calibrated by published PK data (Supporting Information Fig. S5). For the low dose MMF plus glucocorticoid (prednisone) combination regimen, simulations from our reference LN virtual patient showed gradual mitigation of efficacy markers (UPCR, 24 h proteinuria, serum creatinine) and increase in circulating C3 and C4, which were in very good agreement with reported clinical trial data (Fig. 5B–I)^58^^,^^59^. Moreover, model-simulated medium-dose MMF plus prednisone under different tapering regimens can also match well with published clinical data (Fig. 5J–N)^60^, ^61^, ^62^. Furthermore, the 4-drug combination regimen of induction intravenous methylprednisolone (MP) and CTX followed by oral MMF and prednisone was also simulated under the default model parameterization, and our results were highly consistent with reported data (Fig. 5O and P)^17^. In addition to the simulation-data comparison presented in Fig. 5, another nine trials testing different doses/regimens of SoC drugs were also utilized during this calibration process (Supporting Information Figs. S6 and S7). These results together demonstrated that our integrated QSP model and model-based reference virtual patient are in a good position to quantitatively assess LN disease progression and treatment response.

**Figure 5:**
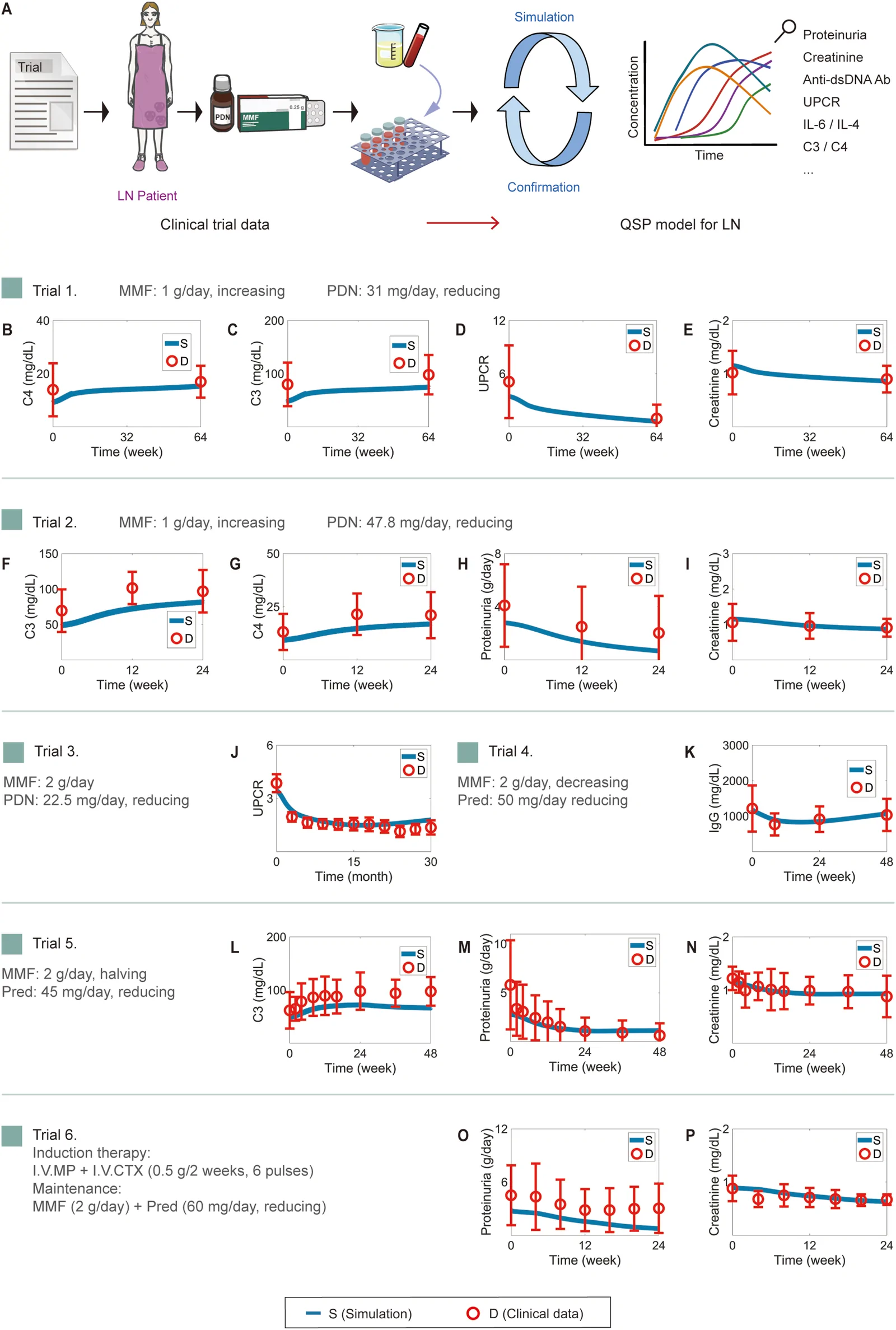
Quantitative calibration of the LN reference virtual patient using clinical trial data. (A) Diagram illustrating that quantitative data of patient biomarker response as reported in a number of clinical trials were used during the calibration process. (B–P) Quantitative comparisons of clinically-reported biomarker response data (red) with model-simulated biomarker response (blue) using our model-based reference virtual LN patient. Therapies including different combinations of mycophenolate mofetil, prednisone and cyclophosphamide were simulated using the same dosing regimen (summarized briefly in the figure) as provided in the original references; biomarker response data used include serum C3/C4, 24 h proteinuria, UPCR, serum creatinine and IgG^17^^,^^58^, ^59^, ^60^, ^61^, ^62^. Details of the dosing regimens are provided in Protocol S1. S, model simulation; D, clinical data.

To further validate the value of our integrated QSP model framework in predicting LN treatment response, we used data from another four independent LN clinical trials using high doses of MMF plus prednisone (Fig. 6A, and Fig. S7I–S7K). As shown in Fig. 6B–K, respective model predictions of reference patient response under three different dosing regimens are in very good quantitative agreement with reported trends of clinical efficacy biomarkers, including UPCR, 24 h proteinuria, circulating C3, C4, IgG, IgM, anti-dsDNA Ab and serum creatinine^63^, ^64^, ^65^. Overall, the high goodness of fit of our model simulations (of a reference virtual LN patient) can also be visualized by comparing all the observed and predicted values of response biomarkers (under all dosing regimens and all timepoints) altogether, with the vast majority of data-to-simulation ratios being close to one (Fig. 6L). We also explored the potential expression changes of various immune-related cytokines in the systemic circulation post SoC treatment; again, the simulations are generally in good qualitative and quantitative agreement with literature data (Supporting Information Fig. S8).

**Figure 6:**
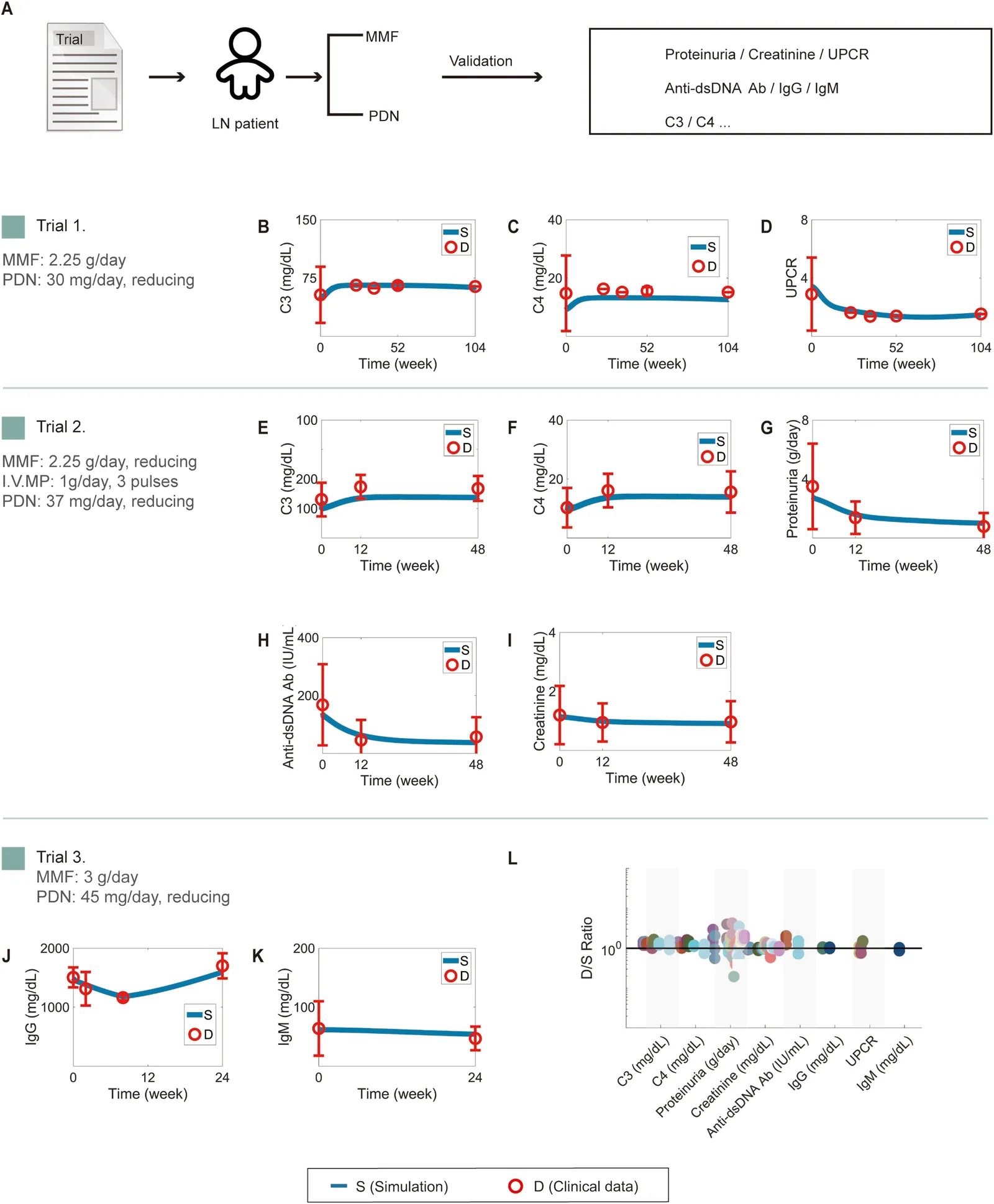
Validation of model-based LN virtual patient using high dose SoC clinical response data. (A) Workflow diagram for validating the model-based reference LN virtual patient. (B–K) Comparison of clinical data (red) with calibrated simulated response (blue) for the “reference LN virtual patient” based on four clinical trials of LN patients on mycophenolate mofetil and prednisone background therapy, with response biomarkers including C3, C4, UPCR, creatinine, 24 h proteinuria, IgG, IgM, anti-dsDNA Ab^63^, ^64^, ^65^. Details of the dosing regimen are given in the Protocol S1. (L) Goodness-of-fit plots showing the ratio of clinical observations to model predictions for patients with lupus nephritis. S, model simulation; D, clinical data; D/S ratio, quantitative ratio of each observed datapoint to corresponding simulated result.

### 3.5 The integrated QSP model enables translational efficacy prediction of MSC-based therapeutic regimens in LN patients

With the aforementioned validated QSP model framework, we seek to assess the clinical treatment response of novel MSC-based regimens in LN patients. We adjusted specific model parameters according to internal *in vitro* (cell-based assays) and *in vivo* data (preclinical biodistribution) obtained for a hUC-MSC product, and we then predicted the potential clinical response to MSC plus low dose background glucocorticoid using our reference virtual LN patient. The predictions were directly compared to an investigator-initiated trial testing this new MSC-based regimen in LN patients (the trial results were not disclosed to the modeling team beforehand). As shown in Fig. 7A and B, our prospective model predictions agree very well with the IIT results in terms of UPCR response after a single dose MSC infusion, and this UPCR response is more potent than that of the classical SoC regimen. This direct validation further drives us to simulate more MSC-containing regimens in LN, and for this purpose we referenced data from two clinical studies of MSCs in LN by Deng et al.^17^ and Gu et al.^20^ (the only two studies published so far). By plugging in the corresponding MSC and SoC regimens as reported in the two studies (assuming averaged similar MSC properties), we found that the simulated treatment response trends again matched well with the clinically reported time-course efficacy data (Fig. 7C–G). To obtain a comprehensive overview of the dynamic biomarker response after MSC treatment, we simulated two intravenous injections of MSCs under a background regimen of MMF and prednisone (tapering) and visualized the results in Fig. 7H. Our simulations suggested that for a reference LN patient, MSCs can potently drive the reduction of classical renal damage-associated markers (*e*.*g*., proteinuria, UPCR) and proinflammatory cytokines while increasing the amounts of circulating C3/C4 and Tregs; these model-predicted trends regarding biomarkers were generally consistent with a number of literature findings (Supporting Information Table S6) which further validates our model.

**Figure 7:**
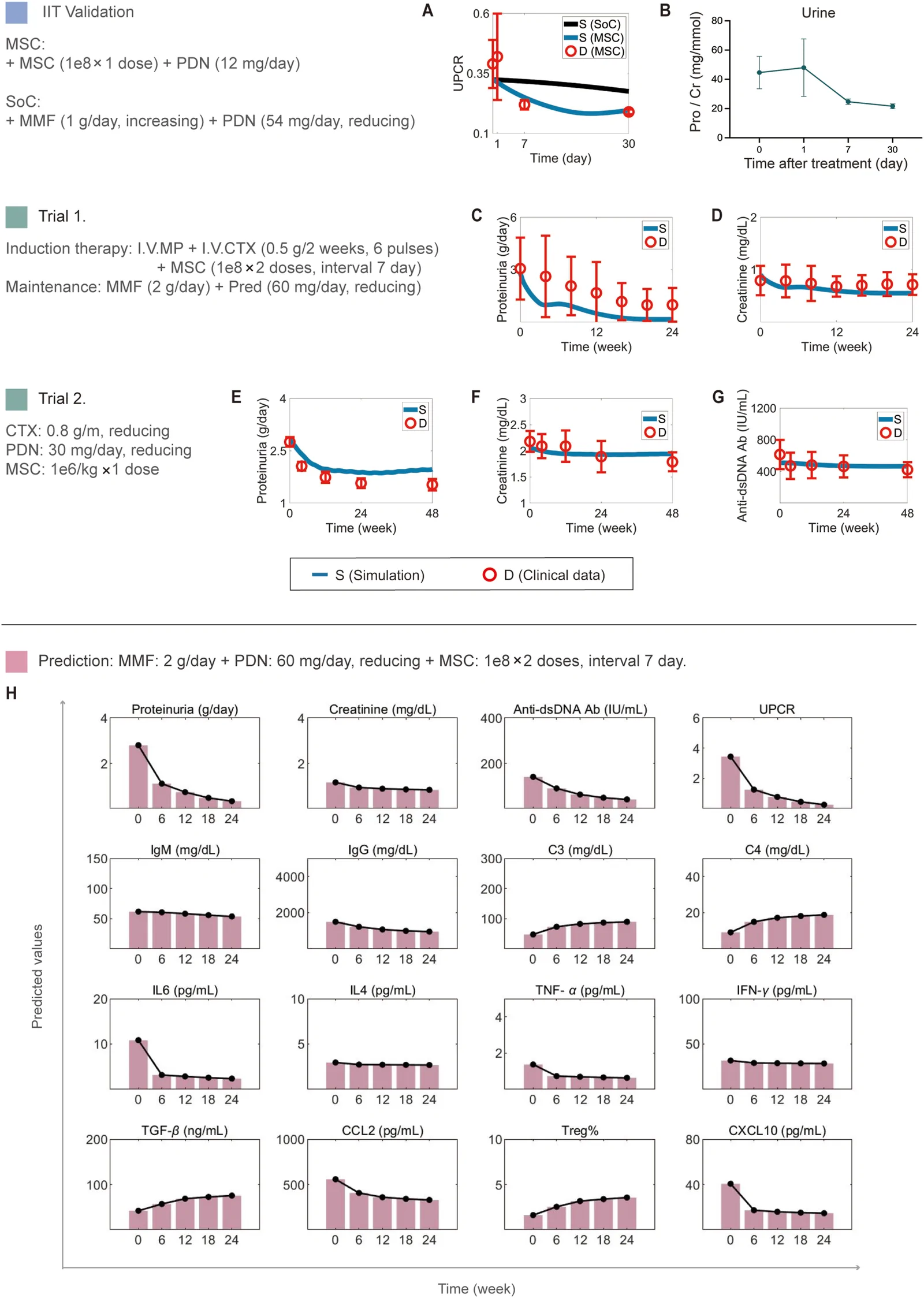
QSP model enables comprehensive characterization of the dynamic biomarker response to MSC-based treatment regimens in LN. (A, B) Model-simulated temporal changes of UPCR are quantitatively validated by clinical observations derived from our in-house IIT with five LN patients being treated with 1 dose of allogenic human umbilical cord-derived MSCs (hUC-MSCs) at 2 × 10^6^ cells/kg (∼1 × 10^8^ cells per patient), and the resulting UPCR profiles are predicted to be superior than that of the SoC regimen. Unitless UPCR values were calculated from the (B) clinically measured raw urine Pro/Cr (proteinuria to creatine) ratios; data are shown as mean ± SEM. (C–G) Additional validation of the QSP model simulations (blue) by referencing clinical efficacy data (red) obtained under complex MSC-containing regimens in LN patients as reported in Deng et al.^17^ and Gu et al.^20^. (H) Predictions of time–course response profiles of various efficacy readouts and circulating biomarkers in LN patients in response to a typical MSC-containing regimen. Details of the dosing regimen are given in the Supplementary Protocol S1. S, model simulation; D, clinical data.

### 3.6 Sensitivity analyses and high-throughput model simulations reveal optimal MSC dosing regimens in LN patients

We next performed global sensitivity analyses using the partial rank correlation coefficient (PRCC) method to investigate the contribution of QSP model parameters (representing different physiological processes) in regulating model output, *e*.*g*., clinical efficacy biomarkers. Using proteinuria as the output of interest, sensitivity analyses revealed that processes relating to podocyte survival, plasma cell and T/B cell survival, and potency of therapy (*e*.*g*., extent of inhibition of lymphocytes by MMF) can most profoundly influence renal inflammatory damage and thereby proteinuria values, and such parameter sensitivities results are similar in both the SoC only scenario and the SoC plus MSC scenario (Fig. 8A and B). Among the T cell-related parameters, enhanced clearance of CD4, CD8, Th1 and Th2 cells were correlated with reduced proteinuria, while clearance of Treg cells were correlated with more severe proteinuria.

**Figure 8:**
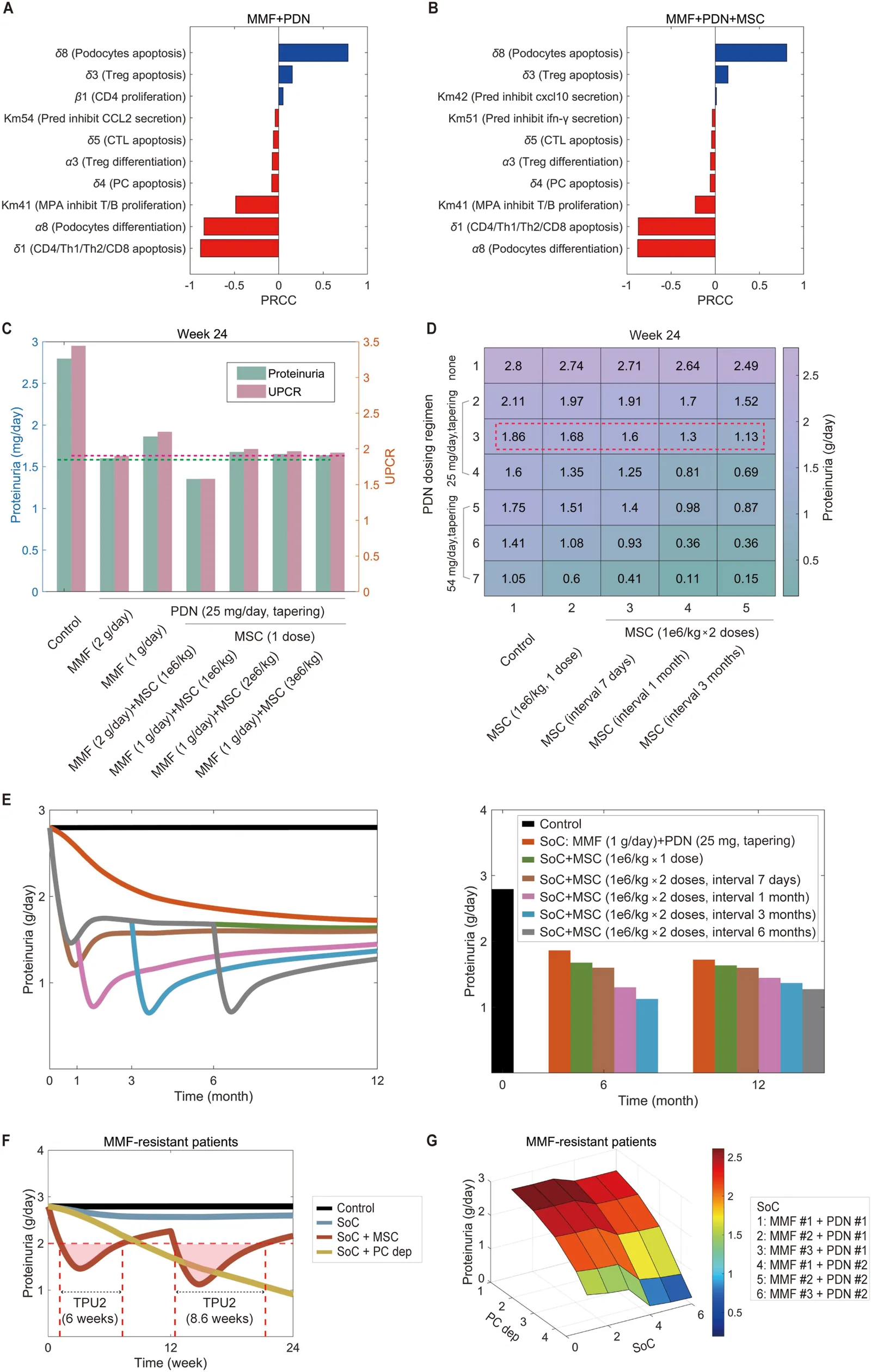
Model-based analyses identified the optimal dosing strategy and comparative advantages of MSCs in LN treatment. (A, B) PRCC indices of parameters that can significantly influence proteinuria values under (A) MMF plus prednisone regimen and (B) MMF plus prednisone and MSC regimen. The positive or negative signs of PRCC values represent positive or negative effects of parameter increase on the model output. Detailed information on the parameters can be found in Supporting Information Table S7. (C) Simulated 24 h proteinuria and UPCR at week 24 under 6 different dosing regimens involving MMF, prednisone and MSCs in LN patients. (D) Simulated 24 h proteinuria outcomes at week 24 (numbers in the matrix) under 35 different dosing regimens involving MMF, prednisone and MSCs in LN patients. Rows #1–7 of the vertical axis of the matrix represent different dosing regimens of MMF (#1, none; #2 and 5, target total dose 0.5 g per day; #3 and 6, target total dose 1 g per day; #4 and 7, target total dose 2 g per day; MMF was administered in bid manners). (E) Time–course simulation of proteinuria response in a reference virtual LN patient under different dosing strategies and quantitative comparisons of 6-month and 12-month proteinuria outcomes derived from the different regimens. (F) Predicted 24-week time–course proteinuria response in MMF-resistant patients under different treatment strategies. PC dep, plasma cell depletion; TPU2, time of proteinuria under 2 g/days. (G) Simulated 24-week proteinuria response surface to different SoC plus PC depletion strategies in MMF-resistant LN patients. PC dep axis represents different intensities of plasma cell depletion (the larger the value, the more plasma cell depletion). MMF#1, 0.25 g twice daily; MMF#2, 0.5 g twice daily; MMF#3, 1 g twice daily; PDN#1, initial dose 25 mg/days, tapering; PDN#2, initial dose 54 mg/days, tapering. Details of the dosing regimens presented here are provided in Protocol S1.

We then seek to comparatively explore the potential efficacy of different MSC-based regimens in LN patients using our high-throughput simulation framework. Compared to control condition (no treatment), the classical combination of MMF and prednisone can significantly improve the 24-week proteinuria values (with higher MMF doses being more efficacious), and the addition of one MSC infusion (on Day 0) can further reduce the proteinuria readouts, indicating better patient remission (Fig. 8C). Interestingly, the addition of one MSC infusion can halve the dose of MMF used and yield similar proteinuria endpoints, indicating potential benefits of MSCs in reducing immunosuppressant usage and related adverse events. However, increasing the MSC dose does not seem to have additional benefit in reducing proteinuria readouts in this scenario (Fig. 8C). We also investigated the impact of different MSC infusion intervals on patient treatment response. For the combination regimen of MSC with different MMF plus prednisone dosages (with tapering), model simulations revealed that two MSC infusions at an interval of 3 months can most significantly improve the proteinuria readout at 6 months (24 weeks) (Fig. 8D). Direct comparisons of MSC-based regimens (MSC plus SoC) to SoC only regimens further showed that MSC-based regimens can lead to a much faster and long-lasting impact in reducing proteinuria values, in both low dose and high dose MMF scenarios (Fig. 8E, and Supporting Information Fig. S9). Thus, it can be concluded from our reference LN patient simulations that the optimal dosing strategy for MSCs would be 2 infusions with a 3-month interval for an observational period of 6 months in an investigational MSC-based regimen (MSC plus SoC), considering feasibility and regulatory requirements in a clinical trial setting. This model prediction together with population-level simulations shown later in Fig. 9 informed and supported the dosing design of the phase 2 randomized control trial of a first-in-class MSC product RY_SW01 in LN patients (ongoing) in which the MSC plus SoC regimen is compared head-to-head to the SoC only regimen. In addition, we have also validated the predictive capacity of our QSP model against quantitative phase 1 trial results of RY_SW01 in LN patients (internal data) in which 3 different doses of MSCs were given.

**Figure 9:**
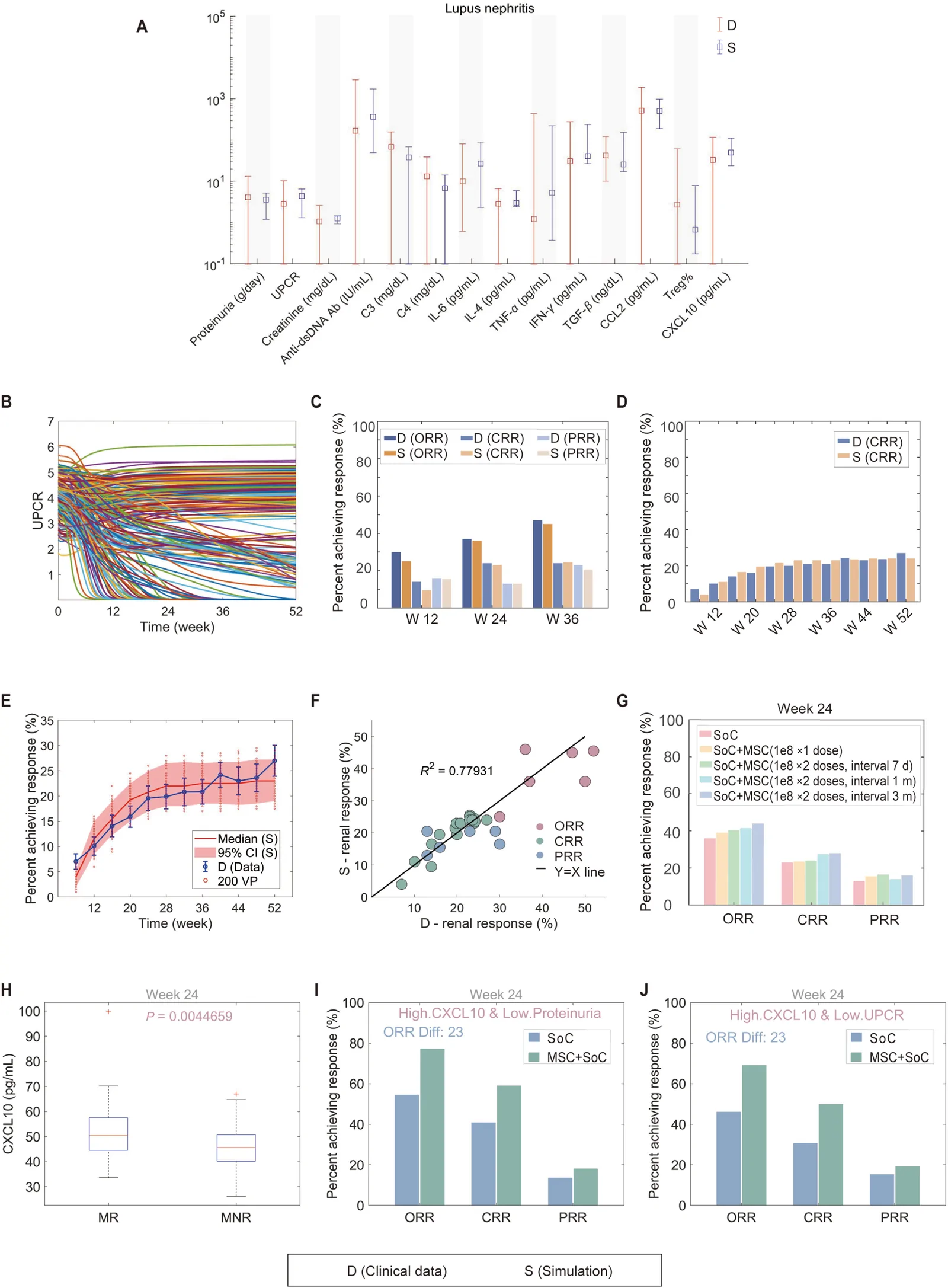
Development and validation of the LN virtual patient population and model-based exploration of potential pretreatment biomarkers for patient stratification. (A) Comparison of the simulated pretreatment biomarker ranges of the LN virtual patient population (*n* = 991) with corresponding clinical data. Data are expressed as ranges and simulated values are expressed as median (range). (B) A representative run of virtual clinical trials under the classical SoC regimen using UPCR as the major observation. (C) Calibration of the virtual patient population using published clinical trial data as reported by Furie et al.^63^ in terms of time-course patient renal response percentages (ORR, CRR, PRR), in which LN patients received the MMF plus prednisone regimen. (D) Validation of the virtual patient population using BLISS-LN trial data^66^ in which LN patients received the MMF plus prednisone regimen (at a different dosing regimen); clinically reported percentages of patients achieving complete renal response (CRR) were quantitatively compared with the simulation results. (E) Repeated high-throughput sampling (100 times) of the virtual patient population demonstrated robust clinical CRR predictions that are consistent with the reported data in the BLISS-LN trial. Red line represents the median model predictions; red shading represents the 5th to 95th prediction ranges; blue line represents the actual clinical data; red dots represent the 100 repeatedly simulated CRR values for a final virtual patient population of *n* = 200. (F) Goodness-of-fit plot summarizing clinical data *versus* model simulations of patients’ renal response percentages in lupus nephritis across the 3 trials used. (G) Simulated 24-week population-level renal response percentages under different MSC dosing regimens using model-based virtual clinical trials. The dosing regimen of SoC was the same as in Fig. 9C. (H) Comparison of pre-treatment circulating CXCL10 abundance distribution in responders *versus* non-responders to MSC therapy (at Week 24) in the virtual patient population. The 25th, 50th, and 75th percentiles are encoded by box plots with whiskers that define 1.5 times the interquartile range away from the bottom or top of the box. MR, responders to MSC therapy. MNR, non-responders to MSC therapy. *P*-values were calculated by *t*-test. (I, J) Simulated 24-week population-level renal response percentages in patient subgroups stratified by the proposed pretreatment biomarker criteria. Predicted ORR differences between the SoC only treatment scenario and SoC plus MSC treatment scenario were calculated for the resulting patient subgroup. High CXCL10 & Low Proteinuria, 48 (pg/mL) ≤ CXCL10 and Proteinuria ≤3.5 (g/days); High CXCL10 & Low UPCR, 48 (pg/mL) ≤ CXCL10 and UPCR ≤ 4.4. S, Simulation; D, Clinical data; Details of the dosing regimens are given in Protocol S1.

We also tested the potential benefit of MSC-based regimens in a reference virtual patient with severe refractory LN that was already resistant to MMF. Although the MSC-based regimen only modestly improved the 24-week clinical endpoint in this patient, this new regimen actually brings substantial clinical benefit over the entire treatment duration if we consider the objective proteinuria reduction in terms of the TPU2 metric (time of proteinuria under 2 g/day): this was maintained for about 15 weeks during the entire 24-week period under the MSC-based regimen, while it is 0 week for the classical SoC regimen (Fig. 8F). We also found that in this particular patient, plasma cell depletion was predicted to be exceptionally efficacious, and we simulated and compared the treatment response surface of proteinuria under different SoC doses combined with varying degrees of plasma cell depletion (Fig. 8G). Together, these results demonstrated that MSCs and depletion of plasma cells may provide unique treatment benefits for highly refractory LN patients and our QSP model can serve as an efficient platform for comparative efficacy evaluation.

### 3.7 Identifying novel LN patient stratification criteria to enhance MSC treatment response through virtual clinical trials and in silico biomarker screening

After we formulated the QSP model and explored the utility of our model-based reference virtual patient in LN translational research, we further extended our modeling analyses from a single patient to the population level to assess population-level efficacy response. Based on the QSP model, we iteratively generated a large plausible virtual population with baseline biomarker characteristics quantitatively consistent to those of real LN patients, from which we then selected the final virtual patient population (VPP) (*n* = 200) using the prevalence-weighting optimization approach (Fig. 9A and B), and quantitative clinical response data from control arms (SoC regimen) of the NOBILITY and AURORA 1 trials were used during this process.^63^^,^^67^ The resulting virtual patient population selected can quantitatively recapitulate the clinically-observed patient response percentages (achieving overall renal response–ORR, complete renal response–CRR, and partial renal response–PRR, using the same clinical criteria) as reported in the two trials at various observational timepoints (Fig. 9C, and Supporting Information Fig. S10). We then validated this virtual patient population by referencing another independent trial (BLISS-LN)^66^ unused during the aforementioned optimization process; again, model-predicted CRR rates and trends under the SoC regimen are highly consistent with the reported clinical data at 12 different observational timepoints from 8 to 52 weeks (Fig. 9D and E). Thus, we have successfully formulated a powerful and robust model-based virtual patient population that is highly reflective of the real-world LN patients (Fig. 9F) in terms of pathophysiology as well as biomarker and treatment response heterogeneity.

We then aim to assess the potential population-level efficacy of different MSC-based regimens in LN patients. After grouping the differential MSC parameter ranges obtained during the fitting processes against different MSC studies as detailed in Fig. 7 and plugging them into the virtual population, we comprehensively simulated different infusion strategies/intervals of typical MSCs (at 2 × 10^6^ cells/kg, equivalent to 1 × 10^8^ cells/patient) in combination with high or low SoC regimens. The results showed that the largest clinical efficacy improvement (percent of ORR) for LN patients at Week 24 would be achieved when MSCs were given at two doses with a 3-month interval (Fig. 9G, and Supporting Information Fig. S11), which is consistent with our prior predictions from the reference single LN patient; this result also explained the seemingly contradictory findings from the small sample size study by Deng et al.^17^ that MSC infusions with a 7-day interval did not result in significantly improved efficacy. Comparatively, two MSC infusions with a 3-month interval in combination with the SoC regimen were predicted to elevate the population-level ORR percentages by an additional 0.2 to 0.4 fold (in average, relative fold increase) compared to SoC regimen alone at Week 24 (Fig. 9G and Supporting Information Fig. S11), and we envision that for longer observational periods with more MSC infusions this clinical benefit will further rise in numbers.

Another important question to the clinical treatment of LN using MSC-based regimens is the identification of optimal patient subgroups. Using our virtual patient population, we ranked all patients in terms of their quantitative biomarker response (*e*.*g*., UPCR) after MSC-based treatment and then picked out the top quartile of patients (with more notable response gain in MSC + SoC *versus* SoC, termed MR) and bottom quartile of patients (without notable response gain in MSC + SoC *versus* SoC, termed MNR) to comparatively assess their pretreatment biomarker distributions. All circulating pretreatment biomarkers were individually assessed and the results (in boxplots) are shown in Supporting Information Figs. S12–S15. According to our analyses, several pretreatment circulating biomarkers were predicted to be significantly higher in responders (MR) *versus* non-responders (MNR) in MSC treatment, and CXCL10 was one of the top hits (Fig. 9H). Then, we conducted model-based high-throughput virtual clinical trials to quantitatively assess the clinical impact of these potential candidate biomarkers in terms of achieving optimal clinical patient stratification. After comprehensively simulating the respective ORR gains in all the biomarker-guided groups (by enumerating all individual markers and possible threshold values) compared to the default full virtual population, we found that the impact of any single biomarker in guiding patient stratification and enhancing subgroup ORR outcomes is rather limited (Supporting Information Figs. S16–S22). Instead, the optimal stratification effect can be achieved by combining CXCL10 with UPCR or proteinuria as the selection criteria, for which the ORR gains in the resulting virtual patient subgroups after implementing appropriate thresholds (high CXCL10 plus low UPCR or proteinuria) are the largest (Fig. 9I and J). Specifically, the absolute ORR percent gain of LN patients stratified by this 2-biomarker criteria can be as high as over 23% (in MSC-based regimen compared to SoC regimen, where SoC drugs were given at high doses), which is significantly larger than the ORR percent gain of ∼8% in the unstratified population. The translational utility of this 2-biomarker criteria is also demonstrated in the SoC low dose scenario (Fig. S11). We also extended such biomarker analyses to a larger calibrated virtual population that is approximately 5-fold in size (Fig. S20). Stratifying the larger virtual patient population using the same 2-biomarker criteria as aforementioned can still result in a ∼14% absolute ORR gain in the resulting LN patient subgroup, which is considerably larger than that of the unstratified population and indicates that more patients may likely benefit from the MSC-based regimen (Fig. S20C and S20D). It can therefore be concluded from our simulations that LN patients with less severe kidney dysfunction (lower UPCR or proteinuria) and higher circulating CXCL10 may more likely become responders to MSC therapies, and this may offer invaluable insights for the future biomarker-enriched clinical trial design of MSC-based regimens in LN.

Mechanistically, we also investigated how LN tissue microenvironment can impact MSC function through the CXCL10 signaling axis. We turned to the receptor for CXCL10, CXCR3, and we stimulated MSCs with kidney homogenates from LN (MRL/lpr mice) or control mice (MRL/MpJ mice) and detected the level of CXCR3 expression on MSCs (Fig. 10A). We found that compared with control group (using kidney homogenate of normal mice), stimulation using kidney homogenates from LN mice significantly upregulated the expression of CXCR3 on MSCs (Fig. 10B). We also found that IFN-*γ* stimulation can significantly upregulate CXCR3 expression on MSCs (Fig. 10C and D). This indicated that the inflammatory environment in the LN renal tissue, especially high-levels of IFN-*γ*, can upregulate the expression of CXCR3 on MSCs, thereby enhancing MSCs’ chemotactic response to CXCL10 and improving overall treatment response. This finding regarding CXCL10–CXCR3 in LN may help identify specific functional groups within the MSC population and potential new quality control indicators for MSC drugs.

**Figure 10:**
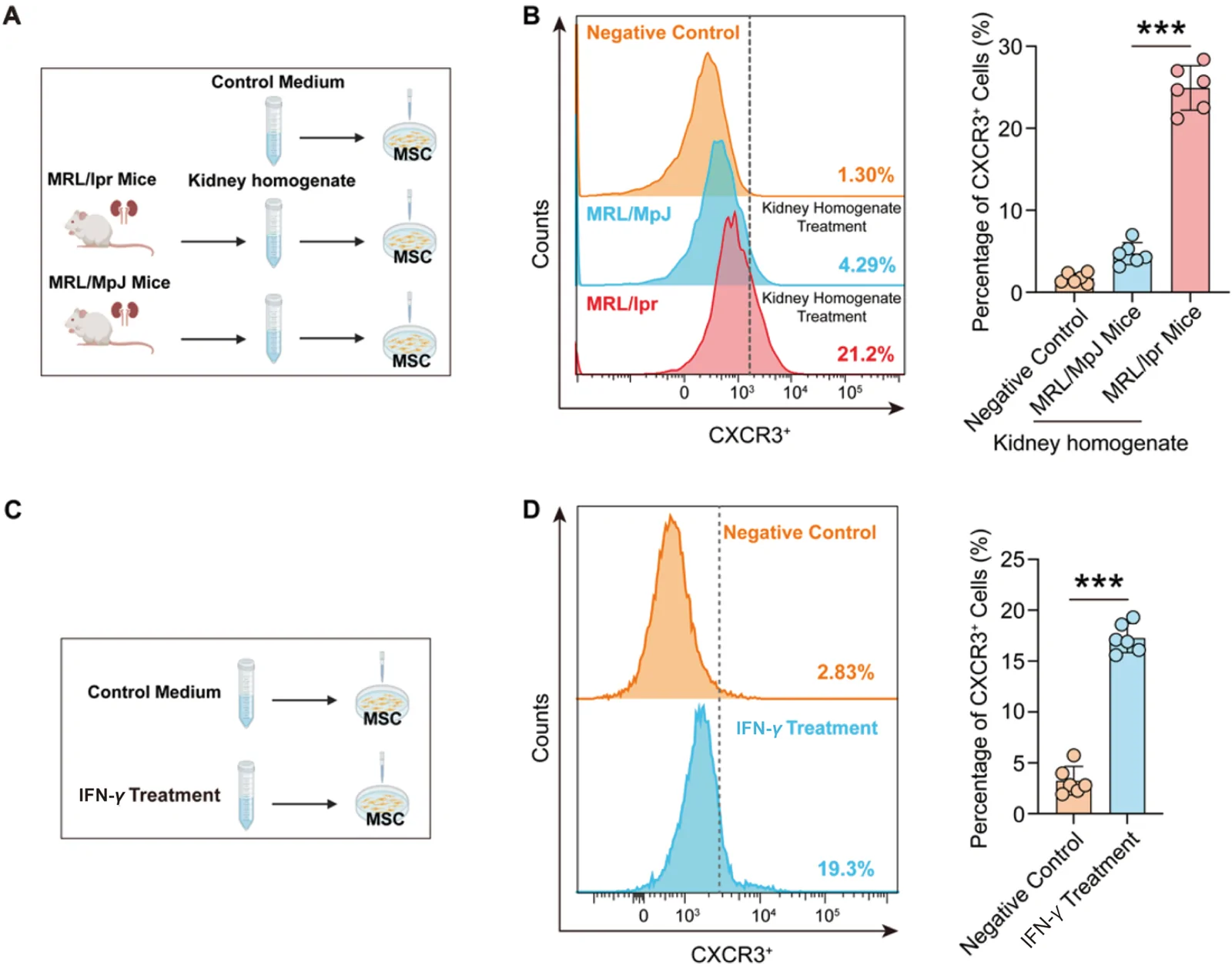
Effects of kidney homogenate from lupus nephritis mice/control mice or IFN-*γ* stimulation on CXCR3 expression on MSC cell membrane. (A) Schematic diagram of MSC stimulation using kidney homogenate from lupus nephritis mice or control mice. (B) The CXCR3 expression level on MSCs stimulated with mouse kidney homogenates for 12 h was determined by flow cytometry (*n* = 6). (C) Schematic diagram of MSC experiments with or without IFN-*γ* stimulation. The concentration of IFN-*γ* was 100 ng/mL, and MSCs were stimulated for 12 h. (D) CXCR3 expression on MSCs stimulated with IFN-*γ* for 12 h was determined by flow cytometry (*n* = 6). Data represent mean ± SD. Two-tailed unpaired equal-variance *t*-test. ∗∗∗*P* < 0.001.

## 4 Discussion

Lupus nephritis (LN) is a complex autoimmune disease in which mesenchymal stem cells (MSCs) are being increasingly explored due to their anti-inflammatory potential. However, mixed findings have been reported so far and optimal MSC clinical dosing design as well as patient selection remain a critical challenge from the clinical drug development perspective^68^. To guide the successful clinical translation of MSCs and bridge the gap between preclinical study results and clinical patient efficacy, we developed a first-of-its-kind QSP model grounded in LN pathophysiology and supported by extensive multiscale data. The model has enabled high-throughput assessment of MSC clinical response in terms of dynamic renal and cytokine biomarker profiles as well as prospective efficacy comparison with SoC drugs. As our QSP model incorporated a variety of immune cells, cytokines and chemokines key to the pathogenesis of LN, it can also serve as a disease platform model to facilitate future drug target identification/validation in LN translational research. In recent years, many candidate therapeutic targets/strategies were proposed and explored by researchers for LN, with a major one being B cell-related interventions, including B cell depletion (*e*.*g*., Rituximab, Obinutuzumab), inhibition of B cell activation (*e*.*g*., Belimumab), immune co-stimulation blockade (*e*.*g*., Abatacept), and anti-plasma cell therapies (*e*.*g*., Bortezomib)^69^. Next-generation approaches such as chimeric antigen receptor T-cell (CAR-T) and CAR-Treg therapies targeting specific B cells are also on the horizon^70^, ^71^, ^72^. The CD40/CD40L axis, a central pathway in immune cell crosstalk, is also under investigation for treating LN (*e*.*g*., BI-655064)^73^. Other potential therapies include miscellaneous cytokine-directed treatments (*e*.*g*., ustekinumab), anti-complement therapies (*e*.*g*., ravulizumab), immunoproteasome inhibitors, kinase inhibitors as well as combinations^69^. Additionally, Voclosporin, a novel immunosuppressive agent in the class of calcineurin inhibitors, was approved for treating LN in 2021^67^. Therefore, it is highly valuable for future efforts to incorporate these emerging target mechanisms into the LN QSP model framework to continuously expand its scope and utility. Indeed, as an immediate next step, we are now actively working on additional modules of B cell life cycle to simulate the translational potential of certain monoclonal and bispecific biologics.

An essential goal of this work is to explore the impact of MSCs in combination with SoC drugs on the clinical response in LN patients (Supporting Information Fig. S23) and how such combinations can provide additional benefits for patients. Interestingly, our modeling analyses showed that by adding MSCs into the treatment regimen, LN patients can achieve more in-depth clinical responses; they can also achieve remission at significantly earlier timepoints and lower the required daily dosages of immunosuppressants, compared to the traditional SoC only regimen (Supporting Information Fig. S24). These model-based predictions are generally consistent with findings from several published trials of MSCs in SLE and LN^74^, ^75^, ^76^, ^77^, ^78^, further reinforcing the validity of our QSP model and stressing that MSCs can likely bring substantial benefits to LN patients in terms of both treatment response and mitigating adverse effects (of SoC drugs). Our modeling analyses also suggested that the dose-response of MSCs is highly nonlinear in LN so that increasing the MSC dose beyond certain thresholds (*e*.*g*., 1 × 10^6^–2 × 10^6^ cells/kg per dose) would not provide additional treatment benefits. This finding is also qualitatively supported by our new *in vitro* data which showed that the cytokine secretion, immune modulatory and migratory capacities of MSCs are not linearly related to cell concentrations (Supporting Information Fig. S25). Similar results were seen in a preclinical rat brain injury model where researchers increased the intravenous MSC dose by 3-fold but did not observe any further enhancement of treatment efficacy^79^. A recent meta-analysis of 914 MSC trials also corroborated our finding and pointed out that the vast majority of published MSC trials that have tested two or more dose levels failed to observe any linear trends of increase in MSC efficacy *versus* dose^80^. Our mechanistic hypothesis is that MSCs, due to their innate anti-inflammatory properties, can act as brakes that can efficiently mitigate (but not eliminate) the vicious hyperactive immune cycles in autoimmune disorders (*e*.*g*., LN). In this sense, simply braking hard once (or twice within a short interval) early on cannot control the system well in the long-run; instead, activating the brake every once in a while would more likely achieve that goal. Therefore, we argue that simply increasing the single MSC infusion dose will not contribute to clinical success, instead, researchers should carefully design the infusion interval and frequency in order to maximally sustain the treatment efficacy of MSCs in the long run. In the clinical trial settings for LN, we proposed that two MSC infusions at 3-month intervals instead of shorter intervals such as 7 days would result in much more significant improvements in long-term response, albeit the shorter interval strategy (with more than two infusions) has been adopted in the MSC-based regimens in treating other acute immune disorders such as acute graft-*versus*-host disease^81^, ^82^, ^83^. Considering that repetitive MSC infusions are generally safe in the clinic^84^^,^^85^, it certainly holds great promise for chronic autoimmune disorders such as LN and SLE that MSC infusions should be administered on a regular frequency basis in order to deliver the best possible benefits for patients^86^. In this sense, our in silico QSP model framework can provide key insights into the optimal cost–effective design of MSC dosing in such clinical treatment scenarios.

In LN, much prior research has established differential correlations between the various circulating cytokines and LN disease activity^87^, ^88^, ^89^; however, no attempts were made to investigate potential efficacy-related predictive biomarkers in the MSC treatment setting. Using our QSP model, we innovatively generated virtual LN patient populations resembling real patient characteristics and behavior, and our simulations predicted that the combination of high CXCL10 with low proteinuria or UPCR could be a potential biomarker of enhanced patient response to MSC-based regimens (compared to SoC alone). This novel finding is consistent with our empirical observations from the preclinical animal experiments with LN mice. It can also be partially explained by our recent findings that elevated CXCL10 in inflamed kidneys can drive the directed migration of hUC-MSCs and that blocking the CXCL10–CXCR3 axis will negatively impact MSC migration and treatment efficacy in experimental LN mice^25^. The chemoattractant potential of CXCL10 in directing MSC migration has also been demonstrated in other preclinical experiments/models of injury and inflammation^90^. For the proteinuria/UPCR component, it can be envisioned that patients with high proteinuria or UPCR typically have more severe immune dysregulations. So in such highly refractory patients, it is less likely that MSCs (despite their strong potency in mitigating immune activation) can deliver sufficient correction of the dysfunction to push these refractory patients into clinical renal responders. Thus, the overall mechanistic hypothesis behind our prediction is that for LN patients with high CXCL10 and low proteinuria/UPCR, more MSCs would migrate toward damaged kidneys (due to CXCL10) and successfully reset the inflammatory tissue microenvironment (of less severe patients). Therefore, the additional treatment impact of MSCs will be most pronounced in this stratified patient subgroup. Despite these biological rationales, this predicted biomarker for LN-MSC patient stratification should be considered in a more qualitative manner, as it would still require further validation at the clinical level and delicate considerations of the thresholding criteria when implementing it toward clinical trials or individual practice. Certain real-world factors may also influence the accuracy of such biomarker measurements. For example, for UPCR which is now commonly used to infer the proteinuria condition for patients, it was reported that the sample collection time (from hour to hour) may considerably influence the readout values^91^^,^^92^, especially at the single-patient level. Hopefully, with more and more emerging patient-specific omics data and machine learning-based analytical techniques becoming available, it can be envisioned that such data-model hybrid framework can enable more feasible biomarkers to be discovered and quantitatively validated toward personalized medicine in LN clinical treatment (including but not limited to MSC-based regimens). Overall, the mechanistic QSP model framework presented here has opened up new simulation-based opportunities for the effective model-informed screening and understanding of different driving factors of MSC treatment response in LN, which will be essential in guiding future clinical research design and personalized medicine implementation.

## 5 Conclusions

We built the first mechanistic QSP model of LN pathophysiology, integrating multimodal preclinical-clinical data, MSC biodistribution and mechanisms, and immunosuppressant pharmacology. Model-based virtual patients well captured interpatient treatment response heterogeneity, and virtual clinical trials identified a spaced two-infusion MSC regimen as the optimal strategy and guided the phase 2 design of a novel MSC therapy. Furthermore, simulations suggested CXCL10 plus proteinuria as predictive biomarkers for enhanced renal response in patients, together demonstrating the importance of our QSP framework in driving MSC clinical development for LN.

## 6 Limitations of the study

While our study has formulated a novel mechanism-based QSP model framework for assessing clinical translational potential of MSC-based therapeutic regimens in LN, a few potential limitations should still be noted. First, as MSC-based therapeutic products are made from live cells, they are inevitably associated with batch-to-batch variations. Also, the source (whether made from bone marrow, umbilical cord, or adipose) could also impact the quality and potency of MSC products. While the current study has implemented a virtual population approach that implicitly incorporated some aspects of potential product-to-product variations, in future the model could still be enriched with more experimental findings on MSC quality control and source differences. Regarding the clinical predictions of multi-drug combinations (involving SoC and MSC), it was currently assumed that the different drugs do not exhibit drug–drug interactions in terms of PK or PD. The reason is that mixed results have been reported on this issue, especially on how different SoC immunosuppressants may influence MSC viability and function^93^^,^^94^. Pooled analysis and more dedicated experiments on this topic may help with better delineating such drug–drug interactions and their potential dose–responses, and this will better inform the future versions of our LN QSP model. Another point is about long-term patient outcome predictions. It has been reported that many LN patients exposed to chronic drug treatments (*e*.*g*., SoC) may experience disease relapse due to loss of treatment efficacy^95^^,^^96^. So far, mechanisms relating to such gradual drug resistance (of SoC or MSC) have not been incorporated into the model, as the exact underlying pathways are still unclear. Thus, model predictions with respect to long-term patient efficacy outcomes (*e*.*g*., >1 year) should be interpreted with caution. Similarly, mechanisms relating to the potential long-term safety concerns of SoC drugs or MSCs are another area for future QSP model enrichment. Last but not least, as patient-level data are always challenging to obtain, we envision that in the near future, by leveraging advanced *in vitro* testing systems such as validated kidney organoids or organ-on-a-chips with immune components^97^^,^^98^, the incoming data sources for our LN QSP model can be further expanded. This will also echo the ongoing trend of reducing animal use and employing new alternative methods (NAMs) in translational drug development.

## Ethics declaration

Written informed consent to take part in the study and to publish the article has been obtained from all participants or their legal representatives. The privacy rights of participants have been observed.

This study included organ or tissue donors. This study includes human biological material and consent was obtained by donors, or their next of kin or legal representatives, for use in this study and for publication of the article. The samples used in this research were not sourced from executed prisoners or prisoners of conscience.

This study was performed in compliance with relevant laws, regulatory frameworks and guidelines where the research took place. This study was approved by the Ethics Committee of The Affiliated Drum Tower Hospital of Nanjing University Medical School (Approval No. SC-2017-002).

This study was conducted in accordance with the ARRIVE (Animal Research: Reporting of In Vivo Experiments) guidelines. This study was approved by the Animal Ethics Committee of China Pharmaceutical University (Approval No. 2021-06-023)

## Author contributions

Chen Zhao, Fang Zhou, Guangji Wang and Lingyun Sun conceptualized and supervised the study. Sujie Zhang implemented the computational model, performed model simulations and analyses. Qixiang Zhang designed and performed the *in vivo* experiments and analyzed the data. Ning Wei and Jing Wang helped with the production of hUC-MSC and experimental data analyses. Qi Rao, Zichun Li, Xia Xiao, Siyuan Yang, Yuchen Qu, Yiyang Xu and Yuan Gao helped with the modeling and data analyses. Dandan Wang and Lingyun Sun conducted the IIT, collected and processed the clinical samples. Sujie Zhang and Chen Zhao wrote the first draft, and all authors contributed to the review and editing of the manuscript.

## Conflicts of interest

The authors declare no conflicts of interest.

## Appendix A Supporting information

The following is the Supporting Information to this article.
