# Supplementary material for: A mechanistic quantitative systems pharmacology framework enriched by multiscale data enables model-informed translational development of mesenchymal stem cell therapy in lupus nephritis
Source: Acta Pharm Sin B. 2026 Aug 25;16(10):6756–79. doi: 10.1016/j.apsb.2026.08.018 (PMC13712365; doi:10.1016/j.apsb.2026.08.018)
Supplement: Supplementary file 1 [file mmc1.pdf]

Supporting Information for

Original article

A mechanistic quantitative systems pharmacology framework enriched by multiscale data enables model-informed translational development of mesenchymal stem cell therapy in lupus nephritis

Sujie Zhang[a,b,†], Qixiang Zhang[c,†], Dandan Wang[d,†], Zichun Li[a], Xia Xiao[a], Qi Rao[a], Siyuan Yang[a], Yuchen Qu[a], Yiyang Xu[a], Ning Wei[e], Jing Wang[e], Yuan Gao[f], Lingyun Sun[d,*], Guangji Wang[c,*], Fang Zhou[c,*], Chen Zhao[a,g,*]

[a]*School of Pharmacy, Nanjing Medical University, Nanjing 210000, China*

[b]*School of Medicine, Yangzhou Polytechnic University, Yangzhou 225000, China*

[c]*Key Laboratory of Drug Metabolism and Pharmacokinetics, State Key Laboratory of Natural Medicines, China Pharmaceutical University, Nanjing 210000, China*

[d]*Department of Rheumatology and Immunology, The Affiliated Drum Tower Hospital of Nanjing University Medical School, Nanjing 210000, China*

[e]*Jiangsu Renocell Biotech Co., Ltd., Nanjing 210000, China*

[f]*QSPMed Technologies, Nanjing 210000, China*

[g]*Department of Pharmacy, the First Affiliated Hospital with Nanjing Medical University, Nanjing 210000, China*

Received 19 July 2025; received in revised form 11 January 2026; accepted 27 February 2026

*Corresponding authors.

E-mail addresses: lingyunsun@nju.edu.cn (Lingyun Sun), guangjiwang@hotmail.com (Guangji Wang), zf1113@163.com (Fang Zhou), chenzhao22@njmu.edu.cn (Chen Zhao).

[†]These authors made equal contributions to this work.

This file includes (2 supplementary protocols, 25 supplementary figures, 8 supplementary tables):

Supplementary Protocol S1. Detailed description of the integrative QSP model.

Supplementary Protocol S2. Virtual population generation and *in silico* patient stratification.

Supplementary Figure S1. LN disease module: calibration of immune cell expansion kinetics against *in vitro* experimental data.

Supplementary Figure S2. LN disease module: calibration of immune cell and podocyte kinetics against *in vitro* experimental data.

Supplementary Figure S3. Experimental comparison of cytokine expression in kidneys of control and MRL/lpr mice.

Supplementary Figure S4. Additional calibration and validation of the PBPK module against independent datasets regarding MSC *in vivo* biodistribution.

Supplementary Figure S5. PK module calibration against time-course plasma profiles of mycophenolic acid (MPA), prednisolone (Pred), methylprednisolone (MP), cyclophosphamide (CTX) and 4-hydroxycyclophosphamide (4-OHCTX) in patients.

Supplementary Figure S6. Additional reference LN virtual patient calibration and validation against published LN trials—part 1.

Supplementary Figure S7. Additional reference LN virtual patient calibration and validation against published LN trials—part 2.

Supplementary Figure S8. QSP model-based simulation of plasma biomarker response in response to SoC therapy.

Supplementary Figure S9. Evaluation of proteinuria response in lupus nephritis patients under various treatment regimens of SoC and MSC.

Supplementary Figure S10. Additional QSP model validation of the final virtual patient cohort against SoC clinical response datasets.

Supplementary Figure S11. Evaluation of biomarker-based patient stratification in 24-week MSC plus SoC therapy using virtual clinical trials—part 1.

Supplementary Figure S12. Model-based screening of potential pretreatment biomarkers in 24-week MSC plus SoC therapy—part 1.

Supplementary Figure S13. Model-based screening of potential pretreatment biomarkers in 24-week MSC plus SoC therapy—part 2.

Supplementary Figure S14. Model-based screening of potential pretreatment biomarkers in 52-week MSC plus SoC therapy—part 1.

Supplementary Figure S15. Model-based screening of potential pretreatment biomarkers in 52-week MSC plus SoC therapy—part 2.

Supplementary Figure S16. Evaluation of biomarker-based patient stratification in 24-week MSC plus SoC therapy using virtual clinical trials—part 2.

Supplementary Figure S17. Clinical response prediction in an expanded LN virtual population.

Supplementary Figure S18. Evaluation of biomarker-based patient stratification in 24-week MSC plus SoC therapy using virtual clinical trials—part 3.

Supplementary Figure S19. Evaluation of biomarker-based patient stratification in 24-week MSC plus SoC therapy using virtual clinical trials—part 4.

Supplementary Figure S20. Evaluation of biomarker-based patient stratification in 24-week MSC plus SoC therapy using virtual clinical trials—part 5.

Supplementary Figure S21. Pretreatment biomarker distribution of virtual patients in 24-week MSC plus SoC therapy.

Supplementary Figure S22. Pretreatment biomarker distribution of virtual patients in 52-week MSC plus SoC therapy.

Supplementary Figure S23. Global sensitivity analysis of MSC-specific modulatory parameters.

Supplementary Figure S24. Predicted 24-week clinical response in MMF-resistant patients in response to different treatment strategies.

Supplementary Figure S25. Effect of different MSC concentrations on the immunomodulatory potential and *in vivo* homing ability of MSCs.

Supplementary Table S1. Calculated reference cell amount values (in lymph nodes and kidneys) used in the LN disease module.

Supplementary Table S2. Calculated reference cytokine/biomarker secretion or production rates used in the LN disease module.

Supplementary Table S3. Reference clinical measurement values of efficacy biomarkers, circulating cytokines and biomolecules in healthy volunteers and LN patients.

Supplementary Table S4. Physiological parameters used in the PBPK module.

Supplementary Table S5. MSC-specific parameters used in the PBPK module.

Supplementary Table S6. General trends of efficacy markers upon MSC treatment as observed in published trials.

Supplementary Table S7. Description of top-ranked sensitive model parameters.

Supplementary Table S8. Proposed MoAs of SoC therapeutics and MSCs in the integrative QSP model.

Supplementary Protocol S1.

Detailed description of the integrative QSP model.

In general, the QSP model includes three body compartments, lymph nodes, kidneys and peripheral blood. As a trigger of the immunity cycle, the process of self-antigen presentation by APCs was simplified by assuming that damaged podocytes can serve as autoantigens to intensify the proliferation and differentiation of T cells. The activated T cells further trigger the proliferation and differentiation of B cells[1]. These immune cells can infiltrate into the kidneys and produce a variety of inflammatory cytokines which potently regulate tissue damage. Below we discuss different modular processes of the immunity cycle included in the LN QSP model (and their associated exemplar equations).

T-cell dynamics

In the model, $CD4^+$ T cells in the lymph nodes are consist of four subpopulations. The first one is naïve $CD4^+$ T cells, as a subpopulation that is at homeostasis in the absence of self antigen and is activated in the presence of self antigen. These cells can proliferate and differentiate into three subtypes, $CD4^+$ T-helper 1 (Th1), $CD4^+$ T-helper 2 (Th2), and Regulatory T cells (Treg). We assumed that the ratio of damaged podocytes in the kidneys represents the antigenic trigger for the autoimmune activation that starts with the activation of these T cells[1]. Then, Th1 and Th2 cells as effector T cells can secrete cytokines to promote their own proliferation, and their terminally differentiated forms are attracted to renal tissues with the help of chemokines CCL2 and CXCL10[2-4]. The last subpopulation is Treg cells, which can also proliferate but their differentiation process is inhibited by the cytokine IL-6[5,6].

Similarly, $CD8^+$ T cells in the lymph nodes are consist of two subpopulations. The first one is naïve $CD8^+$ T cells, as a population that is at homeostasis in the absence of self antigen and can be activated in the presence of self antigen. The naïve $CD8^+$ T cells can proliferate and differentiate into cytotoxic T cells (CTL), which can potently secrete cytokines to promote their own proliferation and infiltrate into renal tissues under the influence of chemokines CCL2 and CXCL10[3,7].

The respective amounts of Th1, Th2, Treg and CTL cells present in the renal tissue were calculated by using a proportional factor (with respect to the corresponding cells in the lymph nodes). The following exemplar equations govern the dynamics of naïve

CD4$^+$ T cell and their sub-populations (*e.g.*, Th1, Treg) in the model, and the dynamic equations for other immune cells (Th2, CD8$^+$ T, CTL) are similar in structure.

$$\frac{dT_{\text{naïve}}^{LyN}}{dt} = K_{pro}^{\text{naïve}} * T_{\text{naïve}}^{LyN} * \left(1 - \frac{T_{\text{naïve}}^{LyN}}{Tmax_{\text{naïve}}^{LyN} * \frac{M^1 * Ag^2}{K^1 + Ag^2}}\right) - \left(K_{diff}^{Th1} + K_{diff}^{Th2} + K_{diff}^{Treg} * \left(1 - \frac{C_{IL6}^{LyN^3}}{K^4 + C_{IL6}^{LyN^3}}\right)\right) * T_{\text{naïve}}^{LyN} - K_{dea}^{\text{naïve}} * T_{\text{naïve}}^{LyN} \quad (1)$$

$$\frac{dT_{\text{Th1}}^{LyN}}{dt} = K_{diff}^{Th1} * T_{\text{naïve}}^{LyN} + K_{pro}^{\text{Th1}} * T_{\text{Th1}}^{LyN} * \left(1 - \frac{T_{\text{Th1}}^{LyN}}{Tmax_{\text{Th1}}^{LyN} * \frac{M^2 * C_{IFN\gamma}^{LyN}}{K^2 + C_{IFN\gamma}^{LyN}}}\right) - K_{dea}^{\text{Th1}} * T_{\text{Th1}}^{LyN} \quad (2)$$

$$\frac{dT_{Treg}^{LyN}}{dt} = K_{diff}^{Treg} * \left(1 - \frac{C_{IL6}^{LyN^3}}{1 + C_{IL6}^{LyN^3}}\right) * T_{\text{naïve}}^{LyN} + K_{pro}^{\text{Treg}} * T_{\text{Treg}}^{LyN} * \left(1 - \frac{T_{\text{Treg}}^{LyN}}{Tmax_{\text{Treg}}^{LyN} * \frac{M^3 * C_{TGF\beta}^{LyN}}{K^3 + C_{TGF\beta}^{LyN}}}\right) - K_{dea}^{\text{Treg}} * T_{\text{Treg}}^{LyN} \quad (3)$$

where T_{Y}^{X} denotes the number of T cells per gram or millilitre of tissue X (LyN = lymph nodes; Kid = Kidney; PB = peripheral blood) in terms of the subtype Y, K_{Z}^{Y} denotes the rate of cell Y in reaction Z (pro = proliferation; diff = differentiation; dea = death), $Tmax_{\text{Y}}^{X}$ denotes the maximum number of T cells (fixed constant) in the subtype Y in the tissue X, C_{A}^{X} the concentration of cytokine A in the tissue X, and M and K are Hill parameters used in the stimulation (or inhibition) of cell proliferation (or differentiation).

B-cell dynamics

In the model, there are primarily two subpopulation of B cells. The first one being naïve B cells, whose proliferation can be activated by CD4$^+$ T cells and have very strong proliferative potential. The second one is plasma cells (being differentiated from naïve B cell) which do not have proliferative capacity but can potently secrete antibodies including IgM, IgG, and anti-double-stranded DNA antibody (anti-dsDNA Ab). The process of B cell differentiation into plasma cells is assumed to be regulated by IL-6

and CD4+ T cells (to represent T follicular helper cells)[8]. The attraction of differentiated plasma cells into renal tissues can be facillitated by chemokines[9,10]. Exemplar equations are shown below.

$$\frac{dB_{\text{naïve}}^{LyN}}{dt} = K_{pro}^{\text{naïve}} * B_{\text{naïve}}^{LyN} * \left(1 - \frac{B_{\text{naïve}}^{LyN}}{Bmax_{\text{naïve}}^{LyN} * \frac{M^5 * T_{\text{naïve}}^{LyN}}{K^5 + T_{\text{naïve}}^{LyN}}}\right) - \left(K_{diff}^{PC} * \frac{M^6 * C_{IL6}^{LyN^3}}{K^6 + C_{IL6}^{LyN^3}} * \frac{T_{\text{naïve}}^{LyN\ 3}}{K^7 + T_{\text{naïve}}^{LyN\ 3}}\right) * B_{\text{naïve}}^{LyN} - K_{dea}^{\text{naïve}} * B_{\text{naïve}}^{LyN} \tag{4}$$

$$\frac{dB_{PC}^{LyN}}{dt} = K_{diff}^{PC} * \frac{M^6 * C_{IL6}^{LyN^3}}{K^6 + C_{IL6}^{LyN^3}} * \frac{T_{\text{naïve}}^{LyN\ 3}}{K^7 + T_{\text{naïve}}^{LyN\ 3}} * B_{\text{naïve}}^{LyN} - K_{dea}^{\text{PC}} * B_{\text{PC}}^{LyN} \tag{5}$$

where B_{Y}^{X} denotes the number of B cells per gram or millilitre of tissue X (LyN = lymph nodes; Kid = Kidney; PB = peripheral blood) in terms of subtype Y, K_{Z}^{Y} denotes the rate of cell Y in reaction Z (pro = proliferation; diff = differentiation; dea = death), $Bmax_{\text{Y}}^{X}$ denotes the maximum number of cells (fixed constant) in the subtype Y in the tissue X.

Cytokine, chemokine and antibody dynamics

In our model, cytokines are secreted by immune cells in the lymph nodes and in the kidneys. Specifically, IFN-γ is secreted by CTL and Th1 cells, TNF-α by Th1 and macrophages, IL-4 by Th2 cells, TGF-β by Treg and macrophages, and IL-6 by Th1, Th2 and macrophages. Chemokines (CCL2, CXCL10) and complement proteins (C3, C4) are secreted by macrophages. Antibodies are mainly secreted by plasma cells. An exemplar equation of cytokine concentration in tissue (lymph nodes) due to cell secretion is shown below for IFN-γ.

$$\frac{dC_{IFN\gamma}^{LyN}}{dt} = K_{sec}^{IFN\gamma} * (T_{\text{Th1}}^{LyN} + T_{\text{CTL}}^{LyN}) - K_{deg}^{IFN\gamma} * C_{IFN\gamma}^{LyN} \tag{6}$$

where K_{Z}^{A} denotes the rate processes of cytokine A in reaction Z (sec = secretion; deg = degradation).

Trafficking of immune cells from lymph nodes to kidneys

In our model, trafficking of Th1, Th2, CTL cells into the kidneys is mainly facilitated by chemokines CCL2 and CXCL10, whereas Treg migration is driven by CCL2 and plasma cells by CXCL10[3,7,11]. An exemplar equation of Th1 cells trafficking into the

kidneys is shown below.

$$\frac{dT_{th1}^{Kid}}{dt} = K_{in}^{Th1} * T_{\mathrm{Th1}}^{LyN} * \frac{C_{ccl2}^{Kid}}{K_{ccl2}^{th1}+C_{ccl2}^{Kid}} * \frac{C_{cxcl10}^{Kid}}{K_{cxcl10}^{th1}+C_{cxcl10}^{Kid}} \quad (7)$$

Where K_{in}^{Y} denotes the proportion of cell Y (relative to its concentration in lymph nodes) that are infiltrating into the kidney, K_{A}^{Y} denote Hill function parameters of cytokine A in regulating cell Y trafficking.

Dynamics of macrophages and podocytes within the kidneys

In the model, macrophages in the kidney are regulated by three cytokines: functional activation by IFN-γ[12,13] and inhibition by TGF-β and IL-4[14-16]. Excessive podocyte killing can be driven by immune cells, inflammatory cytokines, complement proteins, and anti-self antibodies[17-21]. Exemplar equations are shown below.

$$\frac{dN_{M\o}^{Kid}}{dt} = K_{pro}^{M\o} * N_{M\o}^{Kid} * \left(1 - \frac{N_{M\o}^{Kid}}{Nmax_{M\o}^{Kid} * \frac{M^{8} * C_{IFN\gamma}^{Kid}}{K^{8}+C_{IFN\gamma}^{Kid}} * \left(1-\frac{M^{9} * C_{TGF\beta}^{Kid}}{K^{9}+C_{TGF\beta}^{Kid}}\right) * \left(1-\frac{M^{10} * C_{IL4}^{Kid}}{K^{10}+C_{IL4}^{Kid}}\right)}\right) - K_{dea}^{M\o} * N_{M\o}^{Kid} \quad (8)$$

$$\frac{dN_{podo}^{Kid}}{dt} = K_{diff}^{podo} - K_{\mathrm{dea}}^{podo} * N_{podo}^{Kid} * \frac{M^{11} * T_{CTL}^{Kid}}{K^{11}+T_{CTL}^{Kid}} * \frac{C_{IFN\gamma}^{Kid}}{K^{12}+C_{IFN\gamma}^{Kid}} * \frac{C_{dsDNA}^{Kid}}{K^{13}+C_{dsDNA}^{Kid}} * \frac{C_{TNF\alpha}^{Kid}}{K^{14}+C_{TNF\alpha}^{Kid}} * \frac{C_{C3}^{Kid}}{K^{15}+C_{C3}^{Kid}} * \frac{T_{M\o}^{Kid}}{K^{16}+T_{M\o}^{Kid}} \quad (9)$$

where N_{Y}^{X} denotes the number of cell Y (Mø = macrophages; podo = podocytes) per gram or millilitre in kidneys, $Nmax_{\mathrm{Y}}^{X}$ (fixed constant) denotes the maximum number of cell Y (Mø = macrophages; podo = podocytes) in the kidneys.

Cytokine dynamics in serum

In the model, the amount of cytokines in the serum was assumed to be the sum of cytokines present in the peripheral blood and the combined contribution of excessive cytokines produced in the lymph nodes and kidneys. Disease outcome markers such as 24-h proteinuria, UPCR, and serum creatinine were quantitatively correlated with the amount of damaged renal cells (*e.g.*, the proportion of damaged podocytes)[22]. Below are exemplar equations showing the derivation of serum IFN-γ and creatinine levels from other molecular species.

$$\frac{dC_{IFN\gamma}^{PB}}{dt} = IFN\gamma_{base}^{PB} + F_{\mathrm{IFN\gamma}}^{tiss} * \left(\frac{C_{IFN\gamma}^{LyN} * V_{LyN} + C_{IFN\gamma}^{Kid} * V_{Kid}}{V_{PB}}\right) \quad (10)$$

$$\frac{dC_{Scr}^{PB}}{dt} = Scr_{\text{base}}^{PB} + F_{\text{Scr}}^{tiss} * \left(\frac{Nmax_{podo}^{Kid} - N_{podo}^{Kid}}{Nmax_{podo}^{Kid}}\right)^3 \tag{11}$$

where F_{A}^{tiss} represents the relative contribution of cytokine/marker A (in tissue) when calculting serum cytokine/marker A levels, A_{base}^{PB} represents the basal concentration of cytoknie A in peripheral blood, V_X represents the volume of the tissue *X*.

Pharmacokinetics (PK) of mycophenolate mofetil, prednisone and cyclophosphamide

A standard two-compartment PK model was used to describe the circulating concentration of mycophenolic acid (MPA) in serum based on a modification of the model developed by Jiao et al[23]. A standard one-compartment PK model was used to describe the circulating concentration of prednisolone (Pred) and methylprednisolone (MP) in serum as shown below. Amount of drug given was converted to nominal concentration in the central compartment and agglomerated absorption/metabolism parameter K was assumed to be first order and was individually fitted for prednisone, prednisolone, and MP.

$$\frac{dC_{Pred}^{cen}}{dt} = K_{absorb}^{Pred} * C_{PDN}^{cen} - K_{clear}^{Pred} * C_{Pred}^{cen} \tag{12}$$

$$\frac{dC_{MP}^{cen}}{dt} = K_{formate}^{Pred} * C_{MP_{ester}}^{cen} - K_{clear}^{MP} * C_{MP}^{cen} \tag{13}$$

A standard two-compartment PK model was used to describe the circulating concentration of cyclophosphamide (CTX) and its active metabolite 4-hydroxycyclophosphamide (4-OHCTX) in serum upon i.v. administration, as shown below.

$$\frac{dC_{CTX}^{cen}}{dt} = \left(-\left(\frac{Q_{\overleftrightarrow{cen\,per}}^{CTX}}{V_{cen}}\right) * C_{CTX}^{cen} * V_{cen} + \left(\frac{Q_{\overleftrightarrow{cen\,per}}^{CTX}}{V_{per}}\right) * C_{CTX}^{per} * V_{per} - p * Cl * C_{CTX}^{cen} - (1-p) * Cl * C_{CTX}^{cen} \right) \Big/ V_{cen} \tag{14}$$

$$\frac{dC_{4OHCTX}^{cen}}{dt} = (p * Cl * C_{CTX}^{cen} - K_{clear}^{4OHCTX} * C_{4OHCTX}^{cen} * V_{cen})/V_{cen} \tag{15}$$

$$\frac{dC_{CTX}^{per}}{dt} = \left(\frac{Q_{\overleftrightarrow{cen\,per}}^{CTX}}{V_{cen}} * C_{CTX}^{cen} * V_{cen} - \frac{Q_{\overleftrightarrow{cen\,per}}^{CTX}}{V_{per}} * C_{CTX}^{per} * V_{per} \right) \Big/ V_{per} \tag{16}$$

Where C_D^C represents the concentration of drug D in the comparment C (cen = central ; per = periphery), V_C represents the volume of the comparment C, K_E^D denotes the rate of drug D in reaction process E (E: absorption or clearance), $Q_{\overleftrightarrow{cen\,per}}^{D}$ represents intercompartmental volumetric flow of drug D between the central and peripheral compartment. Parameter p defines the proportion of parent drug CTX metabolized into 4-OHCTX and $1 - p$ is the proportion of parent drug CTX eliminated by alternative routes, either renal clearance or through other metabolites. Parameter Cl represents total volumetric clearance of parent drug CTX. CTX infusion was assumed to be completed within two hours in the model.

Mechanisms of action and transport processes of standard-of-care (SoC) drugs

In the model, except for mycophenolate mofetil which was transported to both the lymph nodes and kidneys, it was assumed that the other drugs entered only the renal tissues[24]. To characterise the concentration of drugs in the renal tissues, we made a simplifying assumption that the amount of drug exposure in the tissue is proportional to the amount of drug exposure in the central compartment. In the model, mycophenolate mofetil can exert its immuno-regulatory effects through four reporetd mechanisms[25-29]: (1) inhibition of T and B cell proliferation; (2) promotion of T cell apoptosis; (3) inhibition of antibody secretion; and (4) inhibition of Treg differentiation. Prednisone can exert its immuno-regulatory effects through three reported mechanisms[25,30-35]: (1) promotion of T cell apoptosis; (2) inhibition of cytokine secretion, including TNF-α, IL-6, IFN-γ, CCL2, CXCL10; (3) inhibition of antibody secretion. Cyclophosphamide can exert its immuno-regulatory effect through two reporetd mechanisms[36,37]: (1) promotion of T cell apoptosis; (2) inhibition of antibody secretion. Consistent with the target of drug action, the PK and disease models of different drugs were integrated by exemplar Hill-type functions as follows:

$$C_D^X = P_D^X * C_D^{cen} \tag{17}$$

$$D_{drug}^{inhibit} = 1 - C_D^X/(K_D^X + C_D^X) \quad (18)$$

$$D_{drug}^{induce} = 1 + C_D^X/(K_D^X + C_D^X) \quad (19)$$

Where C_D^X represents the concentration of drug D in the tissue X (LyN = lymph nodes; Kid = Kidney), C_D^{cen} represents the concentration of drug D in the central comparment, P_D^X represents the partition coefficient of drug D in tissue X, $D_{drug}^{inhibit}$ and D_{drug}^{induce} represent the intensity of inhibition or induction of the drug on its respective targets. K_D^X are parameters used in the hill functions.

Physiologically-based pharmacokinetic (PBPK) modeling of MSC biodistribution

The overall PBPK model structure was described in the main text. Since a fraction of MSCs could be arrested in organs and extravasated from the blood circulation, these MSCs are described separately as in the extravascular (tissue) space of organ. The arrest-extravasate-release-deplete life cycle of MSCs was described similar to the framework proposed by Wang et al[38]. Below show the exemplar equations describing concentration of MSCs in the lung vascular space in the PBPK module.

$$\frac{dC_{MSC}^{Lung_{vas}}}{dt} = \frac{\left(Q_{Lung} * \left(C_{MSC}^{Vein} - \frac{C_{MSC}^{Lung_{vas}}}{P_{Lung}}\right) + K_{rel}^{Lung} * N_{MSC}^{Lung_{arr}}\right)}{V_{Lung}} - K_{ext}^{Lung} * C_{MSC}^{Lung_{vas}} \quad (20)$$

$$\frac{dN_{MSC}^{Lung_{arr}}}{dt} = K_{ext}^{Lung} * C_{MSC}^{Lung_{vas}} * V_{Lung} - (K_{rel}^{Lung} * N_{MSC}^{Lung_{arr}} + K_{dep}^{Lung} * N_{MSC}^{Lung_{arr}}) \quad (21)$$

where $C_{MSC}^{X_{vas}}$ denotes the concentration of MSCs in the vascular space within the organ X (can be lung, liver, spleen, kidney, heart, or the rest of body), $N_{MSC}^{X_{arr}}$ denotes the amount of arrested MSCs in the extravascular tissue space of the specific organ, P_X (unitless) denotes the partition coefficient of the organ X, Q_X denotes the blood flow to the organ X, V_X denotes the volume of blood vessels in the organ X, K_{ext}^X denotes the extravasation rate (from vascular space into tissue) of MSCs in organ X, K_{rel}^X denotes the release rate of MSCs (from tissue to vascular space) in the organ X, and K_{dep}^X denotes the depletion rate of MSCs (from tissue) in the organ X.

Mechanisms of action and transport processes of MSCs

In the model, migration of MSCs into the inflamed tissues can be influenced by chemokine CXCL10 and MSCs exert immunosuppressive effects by secreting massive amounts of TGF-β[39,40], which modulates an array of downstream immune-related events including induction of Tregs[41,42]. The "licensing" effect of MSCs in the model depended on IFN-γ and through the IFN-γ-driven expression of indoleamine 2,3-dioxygenase (IDO), MSCs can potently inhibit pro-inflammatory cytokine secretion by T cells[43-47]. Such enforced expression of IDO by MSCs can also downregulate the M1-like inflammatory macrophages in the inflamed kidneys[46]. Below is an exemplar equation showing the derivation of inhibitory effect by MSCs on their downstream targets.

$$D_{MSC}^{inhibit} = (1 - M * N_{MSC}^{Kid_{total}} / (K + N_{MSC}^{Kid_{total}})) * (1 - b * E_{IDO}^{Kid}) \quad (22)$$

where $N_{MSC}^{Kid_{total}}$ denotes the total amount of MSCs in kidney, E_{IDO}^{Kid} denotes the relative amount of IDO expressed by MSCs (value between 0 and 1), and $D_{MSC}^{inhibit}$ denotes the calculated strength of the inhibitory effect of MSCs on cytokine secretion and other pro-inflammatory processes by immune cells (value between 0 and 1).

SoC dosing protocols used in the modeling analyses

We assumed that the weight of an average LN patient is approximately 50 to 60 kilograms as most patients are female.

The dosages *(Fig. 5B–E)* are given as follows[48]: mycophenolate mofetil (single oral target dose of 0.5 g, twice daily, then increased to 0.75 g or 1 g twice daily by 2 to 4 weeks); prednisone (initial oral dose of 31 mg per day for 2 months, then reduced to 12 mg per day).

The dosages *(Fig. 5F–I)* are given as follows[49]: mycophenolate mofetil (single oral target dose of 0.5 g, twice daily, increased to 0.75 g twice daily at week 2, and advanced weekly to maximum dose 1 g three times daily); prednisone (initial oral dose of 47.8 mg per day, with tapering by 10 to 20 percent at one-week or two-week intervals, on the basis of clinical improvement).

The dosages (*Fig. 5J*) are given as follows[50]: mycophenolate mofetil (single oral

target dose of 1 g, twice daily); prednisone (initial oral dose of 22.5 mg per day, tapered by 5 mg every fortnight until 12.5 mg, then maintained at 10 mg for two consecutive weeks, then decreased by 5 mg per week until 5 mg, then decreased by 5 mg per week until 2.5 mg). The median was used as the initial dose for prednisone.

The dosages (*Fig. 5K*) are given as follows[51]: mycophenolate mofetil (single oral target dose of 1 g, twice daily for six months, then reduced to 0.75 g twice daily); prednisolone (presumed initial oral dose of 50 mg per day, tapered to 7.5 mg per day at six months and to 5 to 7.5 mg per day at 12 to 15 months, then maintained at the same dose for at least two years).

The dosages *(Fig. 5L–N)* are given as follows[52]: mycophenolate mofetil (single oral target dose of 1g, twice daily during the first six months, then halved); prednisolone (initial oral dose of 0.8 mg per kilogram of body weight per day, then reduced by 5 mg per day every two weeks until 20 mg per day, after which it was reduced by 2.5 mg per day every two weeks for four weeks and then by 2.5 mg per day every four weeks, until a maintenance dose of 10 mg per day had been reached, at approximately six months). For simplicity, we adjusted the starting dose of prednisolone to 45 mg according to the trial protocol.

The dosages (*Fig. 5O–P*) are given as follows[53]: mycophenolate mofetil (single oral target dose of 1 g, twice daily at week 12 for 12 weeks); methylprednisolone (pulse intravenous 1 g each for 3 consecutive days); prednisolone (starting at oral 60 mg per day for 4 to 6 weeks, then decreasing dose over a period of 3 to 4 months until a maintenance dose of between 5 and 10 mg per day was achieved); cyclophosphamide (six pulses intravenous at a fixed dose 0.5 g every 2 weeks).

The dosages *(Fig. 6B–D)* are given as follows[54]: mycophenolate mofetil (single oral target dose of 1.125 g twice daily); prednisone (initial oral dose of 0.5 mg per kilogram of body weight per day, with taper to 7.5 mg per day by week 12). For simplicity, we omitted short-term cyclophosphamide and the median was used as the initial dose for mycophenolate mofetil.

The dosages *(Fig. 6E–I)* are given as follows[55]: mycophenolate mofetil (single oral target dose of 1.125 g, twice daily for 3 months, then 0.75 g twice daily as maintenance therapy); methylprednisolone (pulses intravenous 1 g each for 3 consecutive days); prednisone (oral starting at 37 mg per day for 1 month, then progressively tapered). For simplicity, we omitted other drugs and the median was used as the starting dose for mycophenolate mofetil and prednisone.

The dosages *(Fig. 6J–K)* are given as follows[56]: mycophenolate mofetil (single oral target dose of 1.5 g, twice daily); prednisone (initial oral dose of 45 mg per day, then tapered starting at Week 5 by 5 mg per day per week, to 10 mg per day by Week 12).

The dosages (*Fig. 7A and B*) are given as follows: MSCs (1 intravenous infusions of 1×10^8 MSCs).

The dosages (*Fig. 7C and D*) are given as follows[53]: mycophenolate mofetil (single oral target dose of 1 g, twice daily for 12 weeks since week 12); methylprednisolone (pulses intravenous 1 g each for 3 consecutive days); prednisolone (starting at oral 60 mg per day for 4 to 6 weeks, then decreasing dose over a period of 3 to 4 months until a maintenance dose of between 5 and 10 mg per day was achieved); cyclophosphamide (six pulses intravenous at a fixed dose 0.5 g every 2 weeks); MSCs (two intravenous infusions of 1×10^8 MSCs each at 7-day interval apart).

The dosages (*Fig. 7E–G*) are given as follows[57]: cyclophosphamide (starting at pulses intravenous 0.8 g per month, then tapered to 0.46 g per month); prednisone (starting at oral 30 mg per day for 1 month, then tapered by 5 mg every 2 weeks until 15 mg per day, then by 2.5 mg every 2 weeks until 7.5 mg per day); MSCs (1×10^6 MSC per kilogram of body weight per infusion). For simplicity, we adjusted the starting dose of prednisone to 30 mg according to the trial protocol.

The dosages (*Fig. 7H*) are given as follows: mycophenolate mofetil (single oral target dose of 1 g, twice daily for 24 weeks); prednisolone (starting at oral 60 mg per day for 4 to 6 weeks, then decreasing dose over a period of 3 to 4 months until a maintenance dose of between 5 and 10 mg per day was achieved); MSCs (two intravenous infusions of 1×10^8 MSCs each at 7-day interval apart).

The dosages (*Supplementary Fig. S6A and S6B*) are given as follows[58]: mycophenolate mofetil (single oral target dose of 1 g, twice daily); methylprednisolone (pulses intravenous 0.5 g each for 3 consecutive days); prednisone (starting at oral 40 mg per day at four-week intervals, then tapered by 5 mg every one week until 20 mg per day, then reduced by 5 mg every two weeks until a maintenance dose of 10 mg per day had been reached); The median was used as the initial dose for prednisone.

The dosages *(Supplementary Fig. S6C and S6D)* are given as follows[59]: mycophenolate mofetil (single oral target dose of 1 g, twice daily for 6 months, then reduced by 25% after the first 6 months and continued after the first year at a dose of 500 mg twice daily); prednisolone (starting at oral 45 mg per day orally and tapered to

reach 10 mg per day at approximately 6 months, then reduced further to 7.5 mg per day after 9 to 12 months from baseline and to 5 to 7.5 mg per day at 12 to 15 months, then maintained at the same dose thereafter). For simplicity, we omitted methylprednisolone and adjusted the starting dose of prednisolone to 45 mg according to the trial protocol.

The dosages (*Supplementary Fig. S6E and S6F*) are given as follows[60]: mycophenolate mofetil (single oral target dose of 0.75 g, twice daily); prednisolone (initial oral dose of 60 mg per day for one month, then tapered at the rate of 10 mg every 2 weeks to 40 mg per day, then tapered at the rate of 5 mg every 2 weeks to 20 mg per day, tapered at the rate of 2.5 mg every 2 weeks to 10 mg per day). The doses of prednisolone were adjusted in accordance with the trial protocol and the guidelines for the treatment of lupus nephritis (LN).

The dosages (*Supplementary Fig. S6G and S6H*) are given as follows[61]: mycophenolate mofetil (single oral target dose of 1 g, twice daily); prednisolone (starting at oral 50 mg per day orally, reduced by 10 mg every 2 weeks until the dose was 40 mg per day, after which it was reduced by 5 mg every 2 weeks, until a maintenance dose of 10 mg per day had been reached, at 4 months). The starting dose of prednisolone was set at 50 mg according to the trial protocol.

The dosages (*Supplementary Fig. S6I–S6L*) are given as follows[62]: mycophenolate mofetil (single oral target dose of 1 g, twice daily); prednisolone (oral 60 mg per day for 4 to 6 weeks, then 40 mg per day for 2 weeks, then the dose was reduced by 5 mg per day every 2 weeks until 20 mg per day, then 2.5 mg per day every 2 weeks until 12.5 mg per day, followed by 2.5 mg per day reduction every 4 weeks until a maintenance dose of between 5 to 10 mg per day was achieved).

The dosages (*Supplementary Fig. S7A–S7D*) are given as follows[63]: mycophenolate mofetil (single oral target dose of 1 g, twice daily); prednisone (starting at oral 30 mg per day orally, then the dose was reduced by 5 mg per day every 2 weeks until 15 mg per day, then the dose was reduced by 2.5 mg per day every 2 weeks until 5 mg per day).

The dosages in *Supplementary Fig. S7E*[64] are similar to that used in Fig. 5F–I.

The dosages (*Supplementary Fig. S7F*) are given as follows[65]: mycophenolate mofetil (single oral target dose of 0.5 g, twice daily, titrated from 0.5 g twice daily in week 1 and 1 g twice daily in week 2, to a target dosage of 1.5 g twice daily in weeks 3); prednisone (initial oral dose of 54 mg per day, then tapered to 11 mg per day at approximately 6 months).

The dosages in *Supplementary Fig. S7G and S7H* [64] are similar to that used in Supplementary Fig. 7F.

The dosages *(Supplementary Fig. S7I–S7K)* are given as follows[66]: mycophenolate mofetil (single oral target dose of 1.5 g, twice daily, reduced to 1 g twice daily at month 3, and advanced to 0.5 g twice daily after 6 months) plus methylprednisolone (pulses intravenous 0.75 g for 3 consecutive days) plus prednisone (initial oral dose of 30 mg per day, tapered by 5 mg every fortnight until 10 mg, then tapered by 2.5 mg every fortnight until 2.5 mg). For simplicity, we omitted short-term cyclophosphamide and speculated on the detailed dosing regimen based on trial data and protocols.

The dosages of prednisone (*Fig. 8C*) are given as follows (similar to AURORA 1 trial)[67]: initial oral dose of 25 mg per day, tapered by 5 mg every fortnight until 15 mg, then maintained at 10 mg for two consecutive weeks, then decreased by 5 mg per week until 5 mg, then decreased by 2.5 mg per week until 2.5 mg.

The dosages (*Fig. 8F and Supplementary Fig. S24*) are given as follows: SoC, consisted of mycophenolate mofetil (single oral target dose of 1 g, twice daily) combined with prednisone (initial oral dose of 25 mg, tapering. The dosing regimen was derived from the prednisone dosing regimen in Fig. 8C); MSC, single total dose of 1×10^8 cells, two doses infused at three-month intervals;

The dosages (*Fig. 8G*) are given as follows: PDN#1, initial oral dose of 25 mg, tapering. The dosing regimen for prednisone was derived from the regimen used in Figure 8C; PDN#2, initial oral dose of 54 mg per day, then tapered to 11 mg per day at approximately 6 months, similar to reported in Appel et al.[65].

The dosages (*Fig. 9B and C*) are given as follows[54]: mycophenolate mofetil (single oral target total dose of 1.125 g, twice daily) plus methylprednisolone (3 intravenous pulses of 0.5 g each) plus prednisone (initial oral dose of 0.5 mg per kilogram weight per day, maximum 60 mg per day, with taper to 7.5 mg per day by Week 12).

The dosages (*Fig. 9D*) are given as follows[66]: mycophenolate mofetil (single oral target dose of 1.5 g, twice daily, reduced to 1 g twice daily at month 3, and advanced to 0.5 g twice daily after 6 months) plus methylprednisolone (pulses intravenous 0.75 g for 3 consecutive days) plus prednisone (initial oral dose of 30 mg per day, tapered by 5 mg every fortnight until 10 mg, then tapered by 2.5 mg every fortnight until 2.5 mg).

The dosages (*Fig. 9H–J*) are given as follows, similar to [54]: SoC, mycophenolate mofetil (target oral total dose 2–2.5 g/days) plus methylprednisolone (a total of 1000–3000 mg intravenous) plus prednisone (initial oral dose 0.5mg/kg/days, maximum 60

mg/days, with taper to 7.5 mg/days by Week 12); MSC+SoC, MSC (1×10^8 cells, two doses infused at three-month intervals) plus SoC.

Supplementary Protocol S2. Virtual population generation and in silico patient stratification

After model scoping and construction, we developed a reference virtual lupus nephritis patient that can match the dynamic clinical response data (of multiple biomarkers) for various therapeutic regimens. Then, to account for interindividual variability that results in the heterogeneous patient response in LN, we successfully developed a virtual patient population (Vpop) that can match both patient biomarker baseline characteristics and dynamic patient population-level responses to SoC therapies. Statistics regarding disease biomarker distribution derived from literature (*e.g.*, mean, standard deviation) were used to estimate the potential variations within a subset of model parameters, and random sampling obeying lognormal parameter value distributions (using parameters of reference virtual patient as the mean, $0.2x$–$1x$ of values as standard deviations) were used to generate individual parameterization sets which in theory could represent a virtual patient. Parameters relating to immune cell turnover (macrophage, $CD4^+$ T, Th1, Th2, Treg, $CD8^+$ T, CTL, plasma cell), cytokine secretion (IL-6, TNFα, IFNγ, CCL2, TGFβ, CXCL10) were varied between potential virtual patients. By repeating the aforementioned sampling steps and iterative checking and screening using available baseline biomarker distribution data, we generated about 1000 plausible virtual patients (from an initial preliminary population of 50,000) that can quantitatively match all the baseline patient biomarker distributions. This was ensured by running every single virtual patient for a minimum of 52 weeks (without interventions) and matching individual patient's biomarkers ranges (baseline and values throughout the simulation timespan) against literature-derived biomarker value ranges (see Supplementary Table S3 for details of biomarker ranges used). The overall workflow of virtual patient generation and refinement followed the classical sample-rejection and prevalence-weighting methods as proposed by prior studies[68-71].

In order to further refine the virtual population so that patient response at the population level can statistically match the actual observation in the real LN patient populations, dynamic patient response data from three clinical trials (including

percentages of patients achieving overall renal response, complete renal response, and partial renal response) were used to achieve the goal, and a final virtual patient cohort consisting of 200 virtual patients selected from the larger virtual patient population were generated through prevalence weighting, with its population-level response calibrated and validated against the observations derived from the three trials. In order to identify potential predictive biomarkers for patient stratification for MSC-based regimens, patients in the final virtual cohort were sorted according to their differential response to MSC plus SoC (by evaluating the absolute efficacy readout improvement under MSC-based regimen compared to SoC alone) and the top 25% (best responders) and bottom 25% (worst responders) of patients were selected. By comparing the distribution of major possible disease-related biomarkers in the best responders versus worst responders, we picked out the candidate predictive biomarkers that were differentially expressed with statistical significance. We then divided the total virtual population into subgroups by setting numerical inclusion criteria with respect to the various candidate predictive biomarkers, and population-level efficacy (as well as efficacy differences, in terms of ORR, CRR and PRR) for the stratified subgroups in response to SoC and MSC-based regimens (SOC plus MSC) were simulated to comparatively select the candidate biomarkers with the greatest potential for patient stratification.

1. Supporting figures

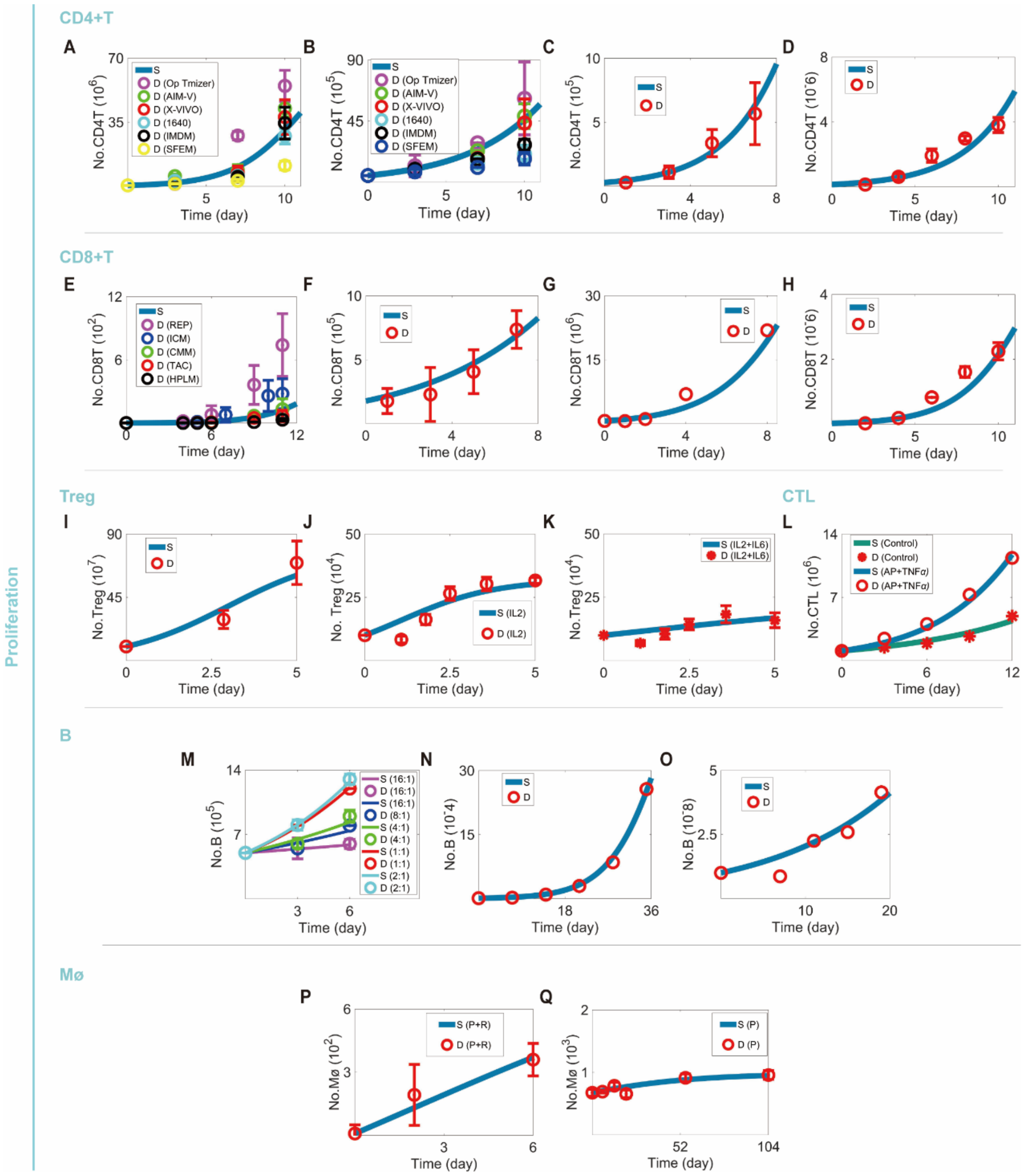

Figure S1 LN disease module: calibration of immune cell expansion kinetics against *in vitro* experimental data. Model simulated and experimentally measured expansion profiles of (A–D) naïve $CD4^+$ T cells[72-74], (E–H) naïve $CD8^+$ T cells[73-76], (I–K) Treg cells[77,78], (L) CTLs[79], (M–O) naïve B cells[80-82] and (P, Q) macrophages[83]. No., number of; S, simulation; D, experimental data; P+R, proliferation and recruitment; P, proliferation; Mø. Macrophages. All data used here are derived from published literature.

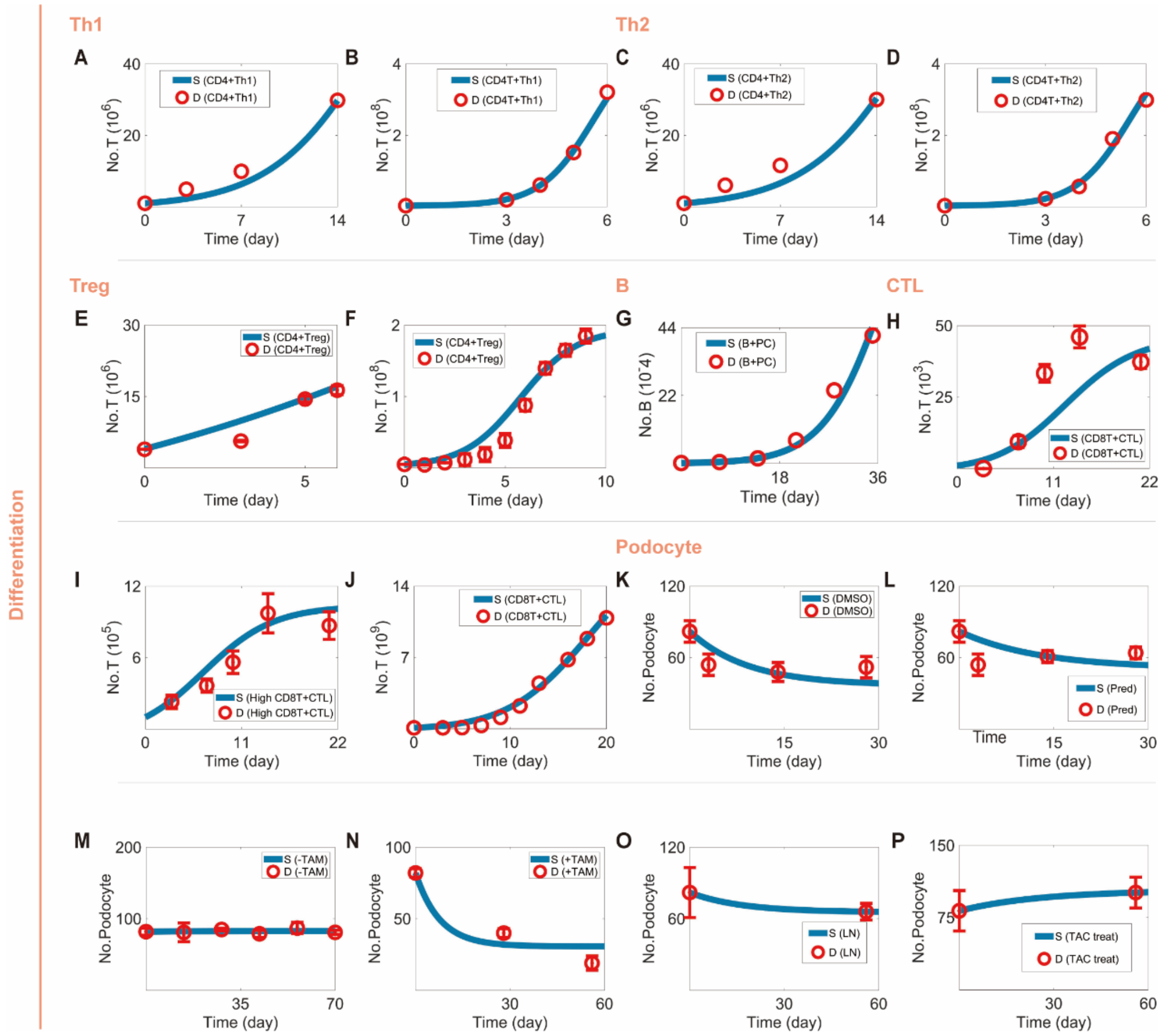

Figure S2 LN disease module: calibration of immune cell and podocyte kinetics against *in vitro* experimental data. (A–F) Simulated and experimentally measured proliferation and differentiation of naïve $CD4^+$ T cells into (A, B) Th1 cells[84,85], (C, D) Th2 cells[84,85] and (E, F) Treg cells[86,87]. (G) Simulated and experimentally measured proliferation and differentiation of naïve B cells into plasma cells[81]. (H–J) Simulated and experimentally measured proliferation and differentiation of naïve $CD8^+$ T cells into CTLs[88,89]. (K–P) Simulated and experimentally measured differentiation kinetics of podocytes[90-92]. No., number of; S, simulation; D, experimental data. All data are derived from experiments in the published literature.

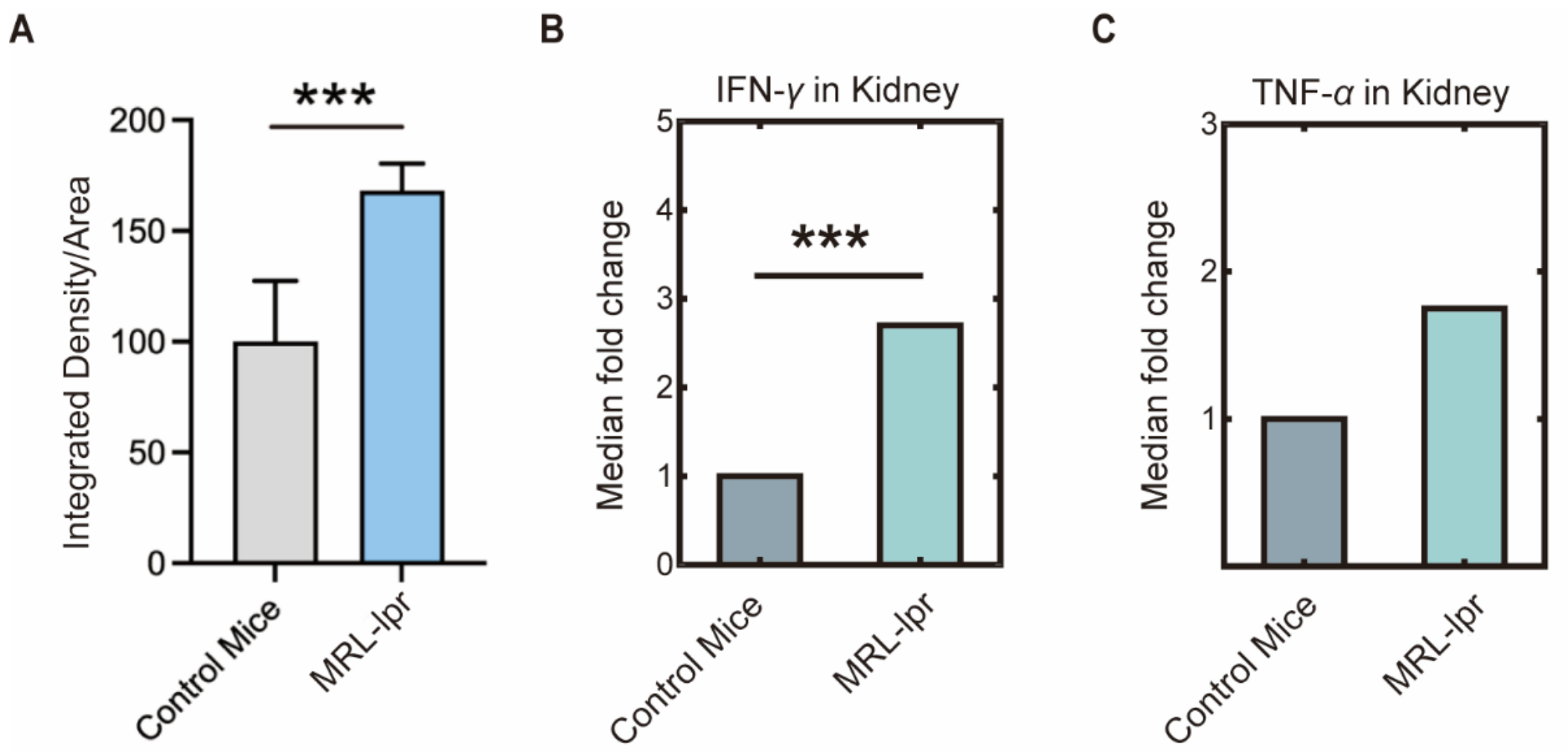

Figure S3 Experimental comparison of cytokine expression in kidneys of control and MRL/lpr mice. (A) Quantified relative CXCL10 mRNA expression in the glomeruli of control and MRL/lpr mice from Fig. 2E. (B, C) Relative fold changes of IFN-*γ* and TNF-*α* protein expression in the kidneys of control and MRL/lpr mice.

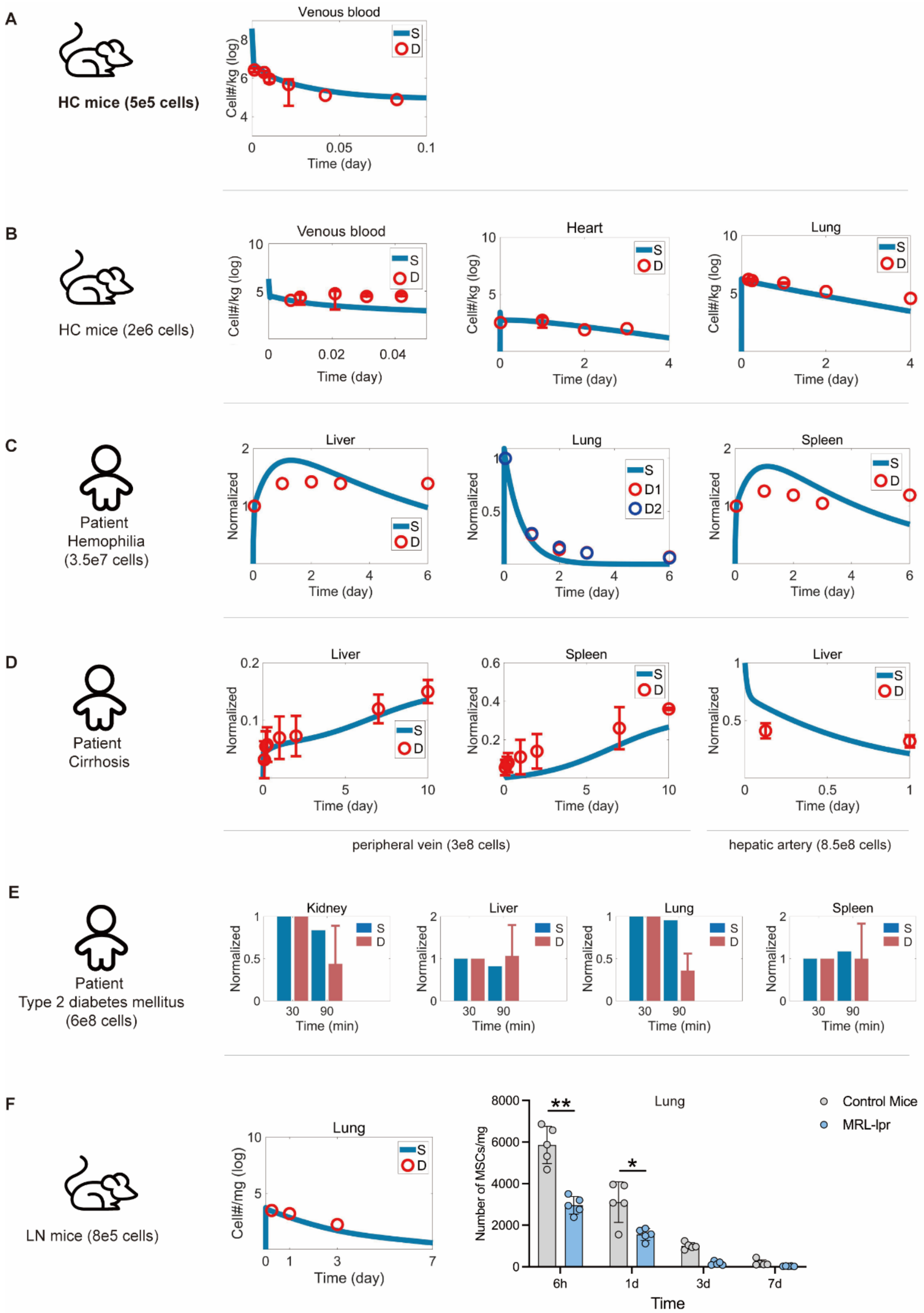

Figure S4 Additional calibration and validation of the PBPK module against independent datasets regarding MSC *in vivo* biodistribution. (A) MSC biodistribution profile in venous blood of BALB/c nude mice after intravenous infusion of 5×10^5 cells[93] and corresponding model simulations. (B) MSC biodistribution profile in venous blood, heart and lung of NOD/SCID mice after intravenous infusion of 2×10^6 cells[94] and corresponding model simulations. (C) MSC biodistribution profile in the liver, lung and spleen of hemophilia patients after intravenous infusion of 3.5×10^7 liver-derived

mesenchymal stem cells[95] and corresponding model simulations. (D) Cell biodistribution profile in the liver and spleen of cirrhosis patients after infusion of 3×10^8 cells (through intravenous)[96], or 8.5×10^8 cells (through hepatic artery)[97], and with corresponding model simulations. (D, Left and middle) *Y*-axis values represent the proportion of organ radioactivity relative to whole body radioactivity at different time points. (D, Right) Eight patients received bone marrow mononuclear cells via the hepatic artery, and *Y*-axis values represent the proportion of liver radioactivity relative to whole body radioactivity at different time points. (E) Cell biodistribution in the kidney, liver, lung and spleen in patients with type 2 diabetes mellitus after intravenous infusion of 6×10^8 BMMNCs[98]. (C–E) Y axes are relative levels (values in C were normalized to their respective maximum values, and values in E were normalized to their respective control values). (F, Left) MSC biodistribution profile in the lung of MRL/lpr mice after intravenous infusion of 8×10^5 cells (and corresponding model simulations). (F, Right) Detection of hUC-MSCs in the lung of mice (8×10^5 cells/mouse) performed using the qPCR method. Cell/kg(log) indicates concentration of MSCs expressed as the number of cells per kilogram of tissue (log10 transformed). S, simulation; D, experimental data.

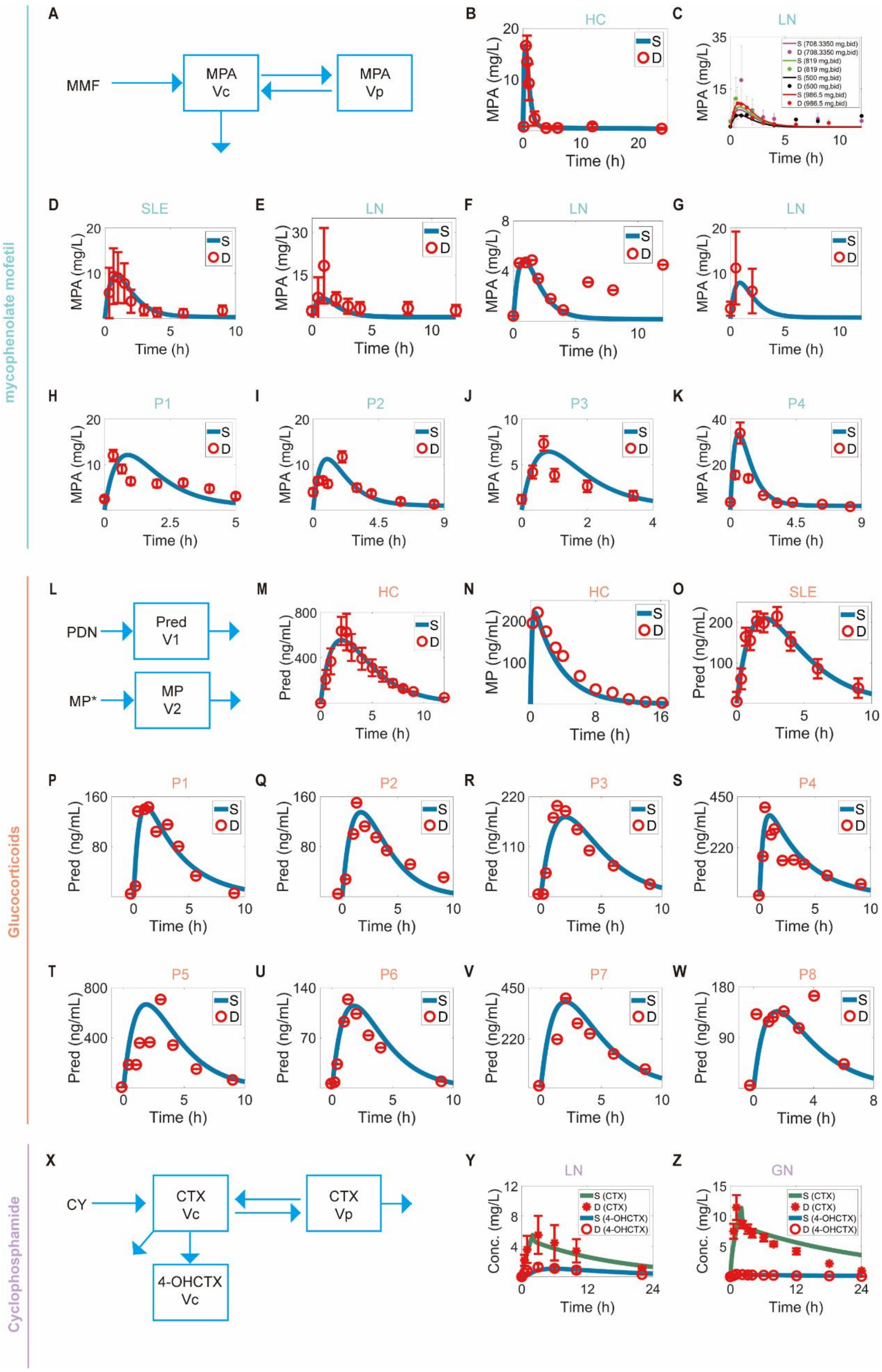

Figure S5 PK module calibration against time-course plasma profiles of mycophenolic acid (MPA), prednisolone (Pred), methylprednisolone (MP), cyclophosphamide (CTX) and 4-

hydroxycyclophosphamide (4-OHCTX) in patients. (A) Pharmacokinetic module diagram of mycophenolate mofetil (MMF). (B) Simulated and reported plasma time-course concentration profiles of the metabolite MPA after administration of a single oral dose of 500 mg MMF in healthy volunteers (data from Ref. 23). (C) Summary of plasma time–course concentration profiles of MPA after administration of single oral dose of MMF in patients (showing results from D–G). (D) Simulated and reported plasma time–course concentration profiles of MPA after administration of a single oral dose of 986.5 mg MMF in patients with childhood-onset systemic lupus erythematosus (data from Ref. 99). (E–G) Simulated and reported plasma time–course concentration profiles of MPA in lupus nephritis patients after administration of a single oral MMF dose of (E) 708.3350 mg (data from Ref. 100), (F) 500 mg (data from Ref. 101), (G) 819 mg (data from Ref. 102), respectively. (H–K) Simulated and reported plasma time–course concentration profiles of MPA after administration of a single oral dose of MMF in four individuals with childhood lupus (data from [103]). The single oral doses of MMF given were (H, I) 728 mg, (J) 350 mg, and (K) 1250 mg, respectively. (L) Pharmacokinetic module diagram of prednisone (PDN) and prednisolone. (M) Simulated and reported plasma time–course concentration profiles of prednisolone after administration of a single oral dose of 20 mg prednisolone in healthy volunteers (data from Ref. 104). (N) Simulated and reported plasma time–course concentration profiles of MP after intravenous single dose of 21.1 mg methylprednisolone sodium succinate (MP*) in healthy volunteers (data from Ref. 105). (O) Simulated and reported plasma time–course concentration profiles of prednisolone after administration of a single oral dose of 19.4 mg PDN in young patients with systemic lupus erythematosus (data from Ref. 106). (P–W) Simulated and reported plasma time–course concentration profiles of prednisolone after administration of a single oral dose of PDN in eight individuals from panel O. The single oral dose of PDN given were (P, R) 5 mg, (Q) 15 mg, (S, W) 20 mg, (T) 40 mg, (U, V) 25 mg, respectively. (X) Pharmacokinetic module diagram of cyclophosphamide (CTX). (Y) Simulated and reported plasma time–course concentration profiles of CTX and metabolite 4-OHCTX after intravenous single dose of 600 mg CTX in lupus nephritis patients (data from Ref. 107). (Z) Simulated and reported plasma time–course concentration profiles of CTX and 4-OHCTX after intravenous single dose of 1600 mg CTX in glomerulonephritis patients (data from Ref. 108). S, simulation; D, experimental data.

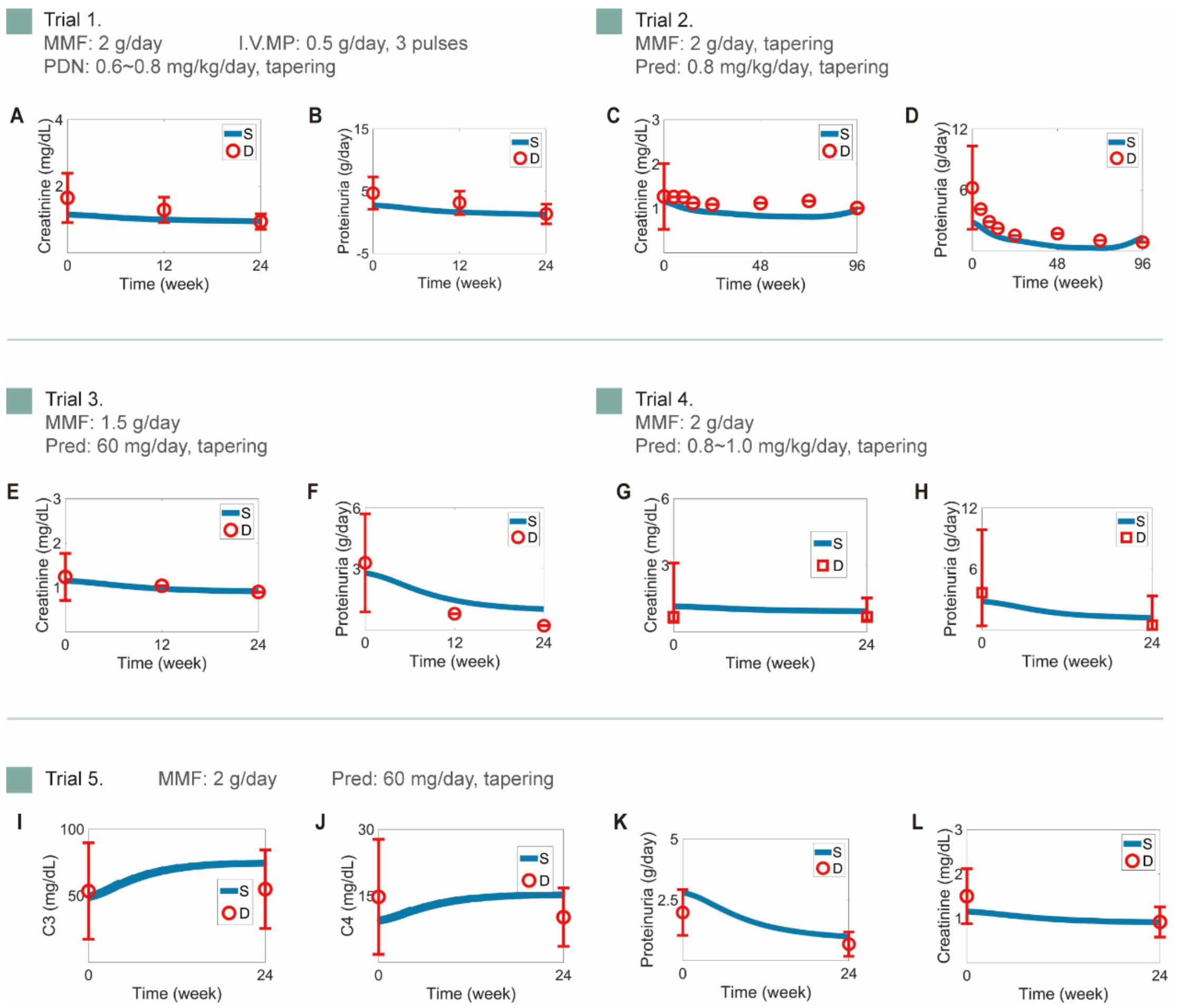

Figure S6 Additional reference LN virtual patient calibration and validation against published LN trials–part 1. (A–L) Calibration and comparison of clinical biomarker response data (red) with model simulations (blue) for the model-based "reference LN virtual patient". Clinical data were derived from published trials in LN patients on mycophenolate mofetil and prednisone therapy, including C3, C4, proteinuria and creatinine (A and B, data from Ref. 58; C and D, data from Ref. 59; E and F, data from Ref. 60; G and H, data from Ref. 61; I–L, data from Ref. 62). S, simulation; D, clinical data.

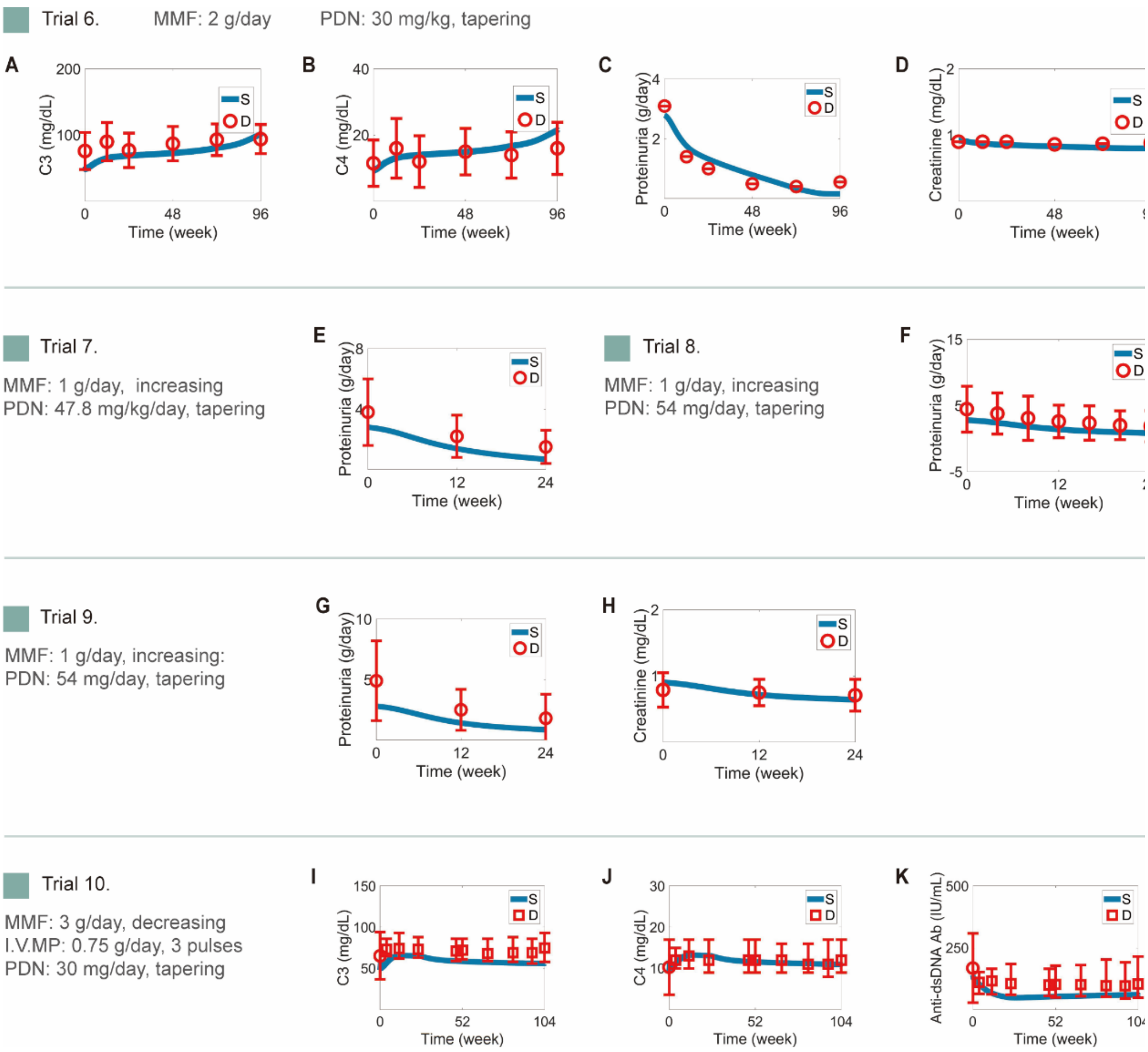

Figure S7 Additional reference LN virtual patient calibration and validation against published LN trials–part 2. (A–K) Calibration and comparison of clinical biomarker response data (red) with model simulations (blue) for the model-based "reference LN virtual patient". Clinical data were derived from published trials in LN patients on mycophenolate mofetil and prednisone therapy, including C3, C4, proteinuria, serum creatinine and anti-dsDNA Ig (A–D, data from Ref. 63; E, data from Ref. 64; F, data from Ref. 65; G–H, data from Ref. 64; I–K, data from Ref. 66). S, simulation; D, clinical data.

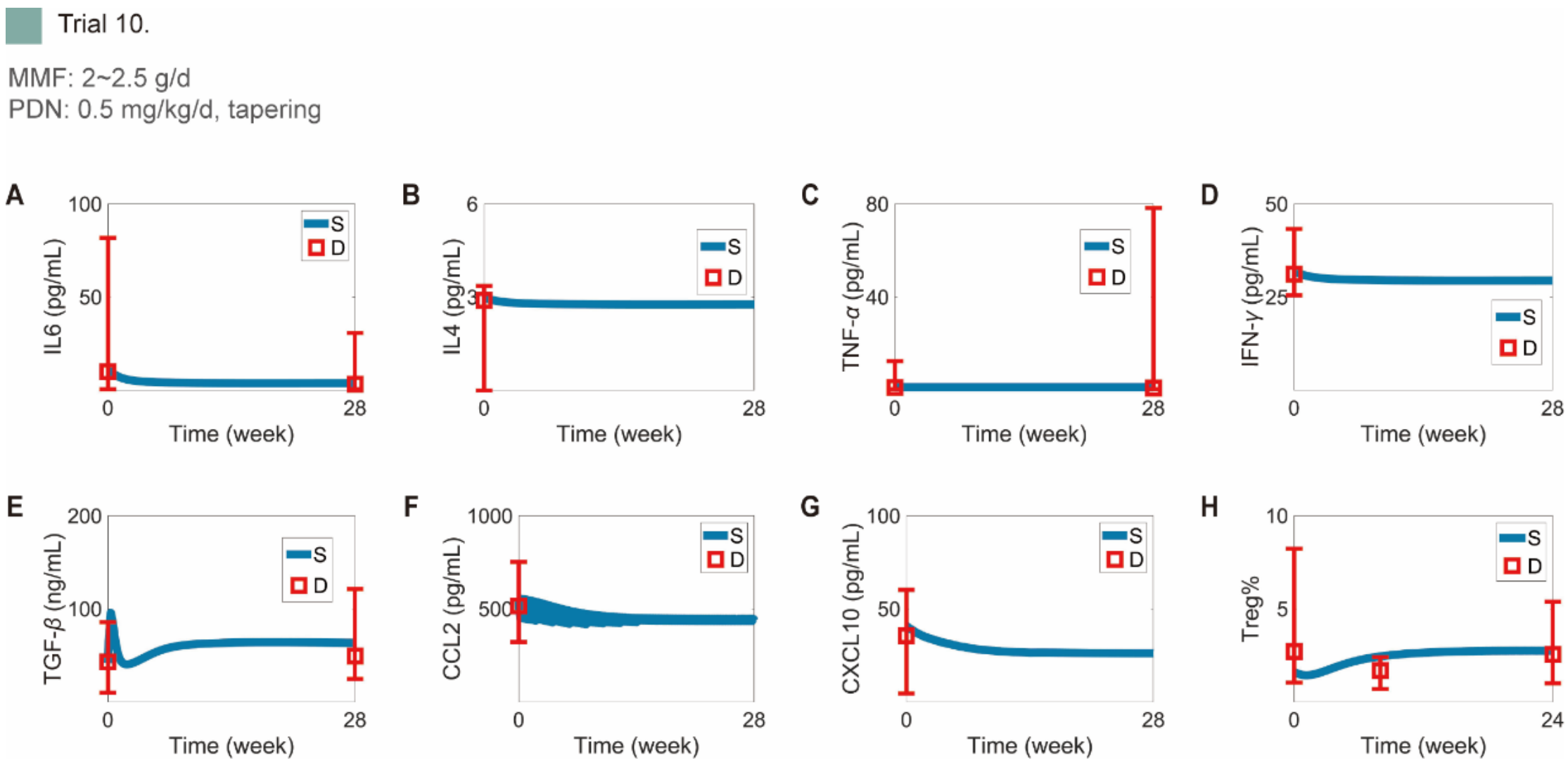

Figure S8 QSP model-based simulation of plasma biomarker response in response to SoC therapy. (A–H) Quantitative comparison of reported clinical data (red) with simulated biomarker values (blue) for a "reference LN virtual patient" under SoC treatment regimens, including IL-6 (data from Ref. 109), IL-4 (data from Ref. 110), TNF-α (data from Ref. 109), IFN-γ (data from Ref. 111), TGF-β (data from Ref. 109), CCL2 (data from Ref. 112), CXCL10 (data from Refs. 113,114), Treg% (data from Ref. 115). The dosages in the panels are given as follows: mycophenolate mofetil total dose 2.25 g/day; Prednisone initial dose 0.5 mg/kg/day, then the dose was reduced by 5 mg/day every 2 weeks until 10 mg/day; followed by 2.5 mg/day reduction every 2 weeks until a maintenance dose of 2.5 mg/day was achieved; administered for 24 weeks. S, simulation; D, clinical data.

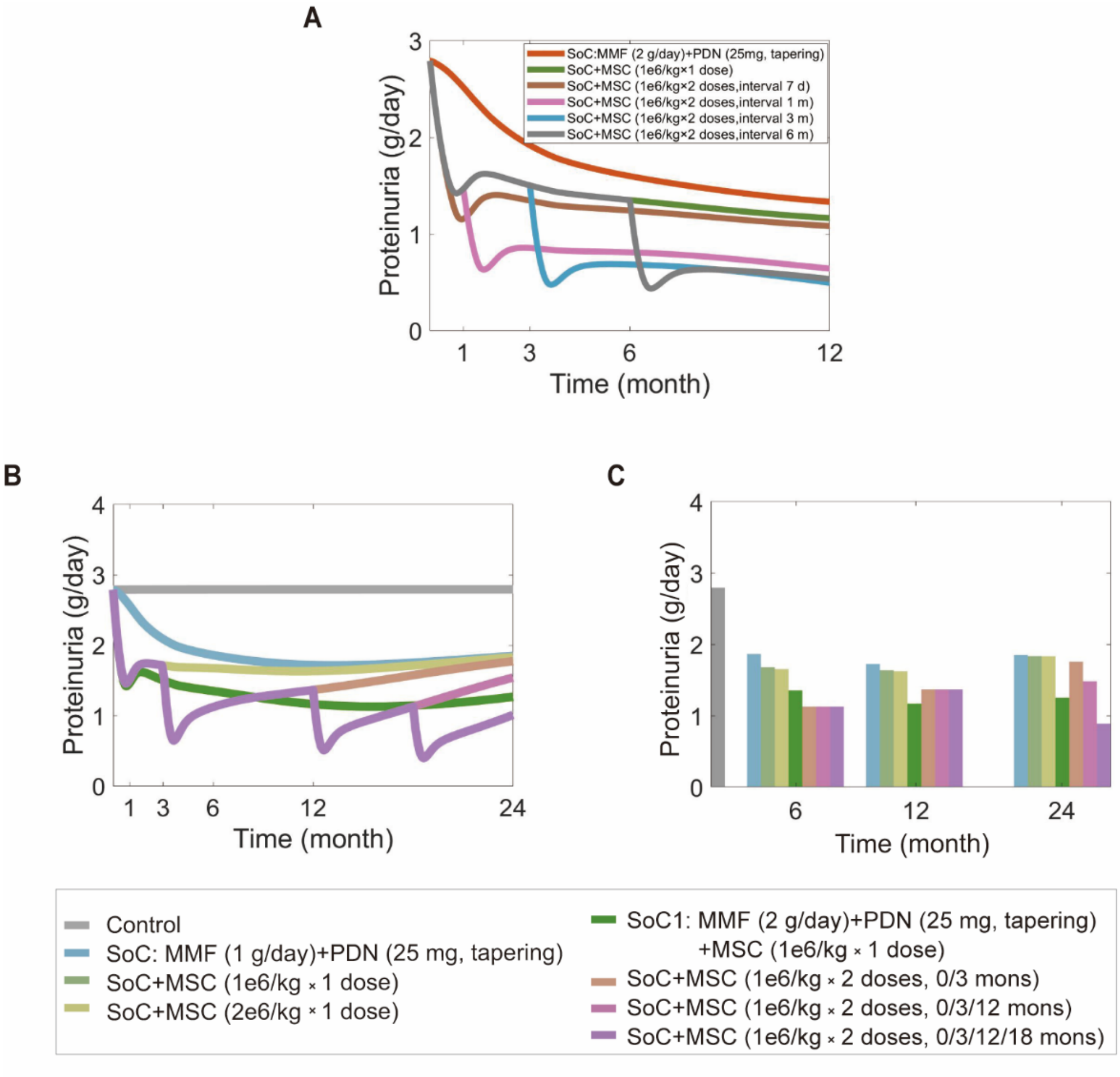

Figure S9 Evaluation of proteinuria response in lupus nephritis patients under various treatment regimens of SoC and MSC. (A) Comparison of 12-month patient 24 h proteinuria profiles under different MSC dosing regimens. The dosages of prednisone are given as follows (similar to AURORA 1 trial)[67]: initial dose of 25 mg/day, tapered by 5 mg every fortnight until 15 mg/day, then maintained at 10 mg/day for two consecutive weeks, then decreased by 5 mg per week until 5 mg/day, then decreased by 2.5 mg per week until 2.5 mg/day. (B) Time–course efficacy biomarker profile (proteinuria) during a course of 24 months in response to different SoC/MSC treatment regimens in a reference virtual patient. (C) Simulated proteinuria outcomes at 6, 12, 24 months in response to different SoC/MSC treatment regimens in a reference virtual patient.

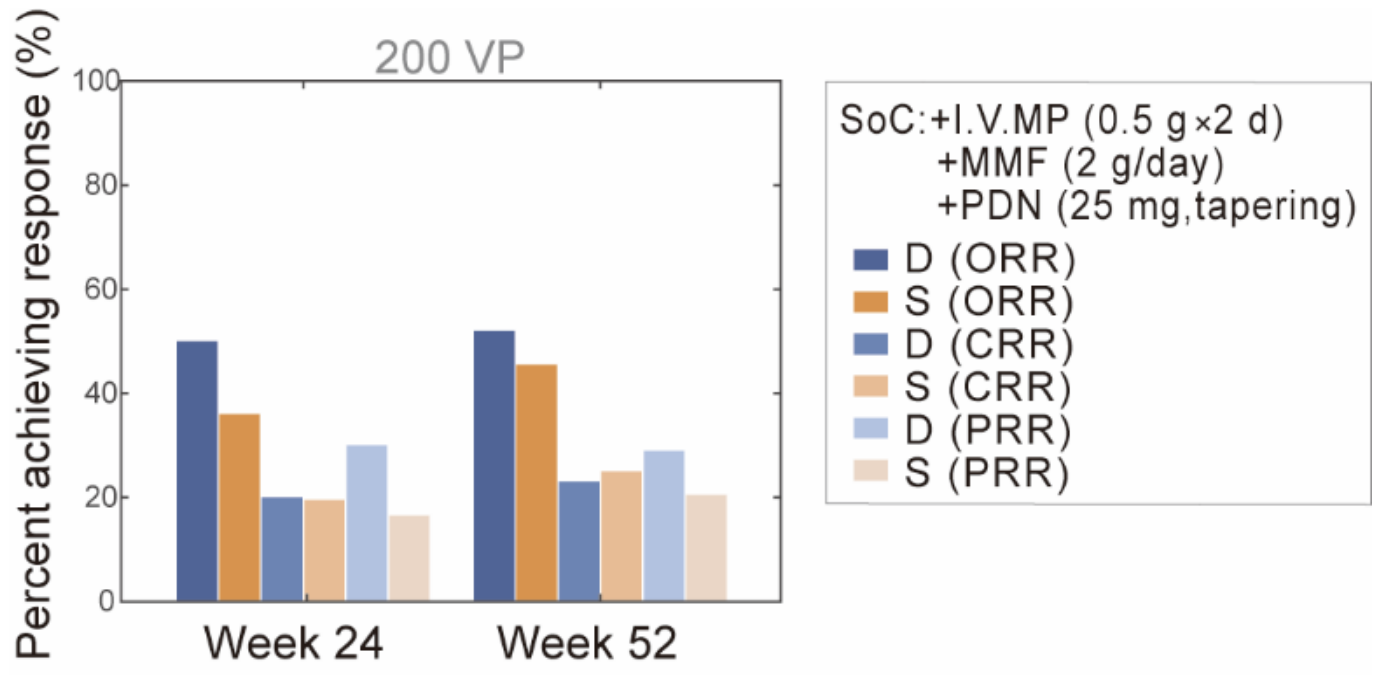

Figure S10 Additional QSP model validation of the final virtual patient cohort against SoC clinical response datasets. Validation of model-based virtual patients using clinical data on renal response rates (blue) and comparisons with simulated population-level patient response rates (orange). Clinical data were derived from the AURORA 1 trial and the SoC dosages are given as follows[67]: mycophenolate mofetil (single target total dose of 1 g, twice daily); methylprednisolone (0.5 g for 2 consecutive days); prednisone (25 mg per day, then tapered to 2.5 mg/day). S, simulation; D, clinical data. ORR, overall renal response; CRR, complete renal response; PRR, partial renal response.

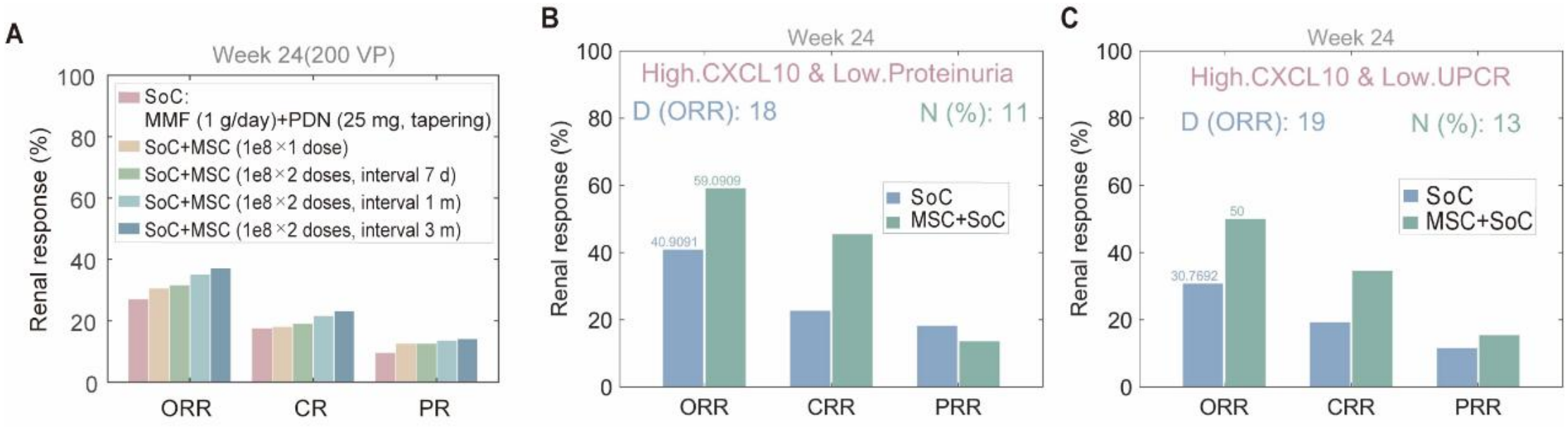

Figure S11 Evaluation of biomarker-based patient stratification in 24-week MSC plus SoC therapy using virtual clinical trials–part 1. (A) Simulated 24-week patient renal response rates under different dosing regimens of MSC and SoC. (B, C) Simulated 24-week patient renal response rates under the MSC plus SoC regimen in biomarker-stratified patient subgroups. D(ORR), the renal response rate differences between SoC and MSC treatment scenarios. N (%), subgroup size compared to the original virtual patient population (in percentages).

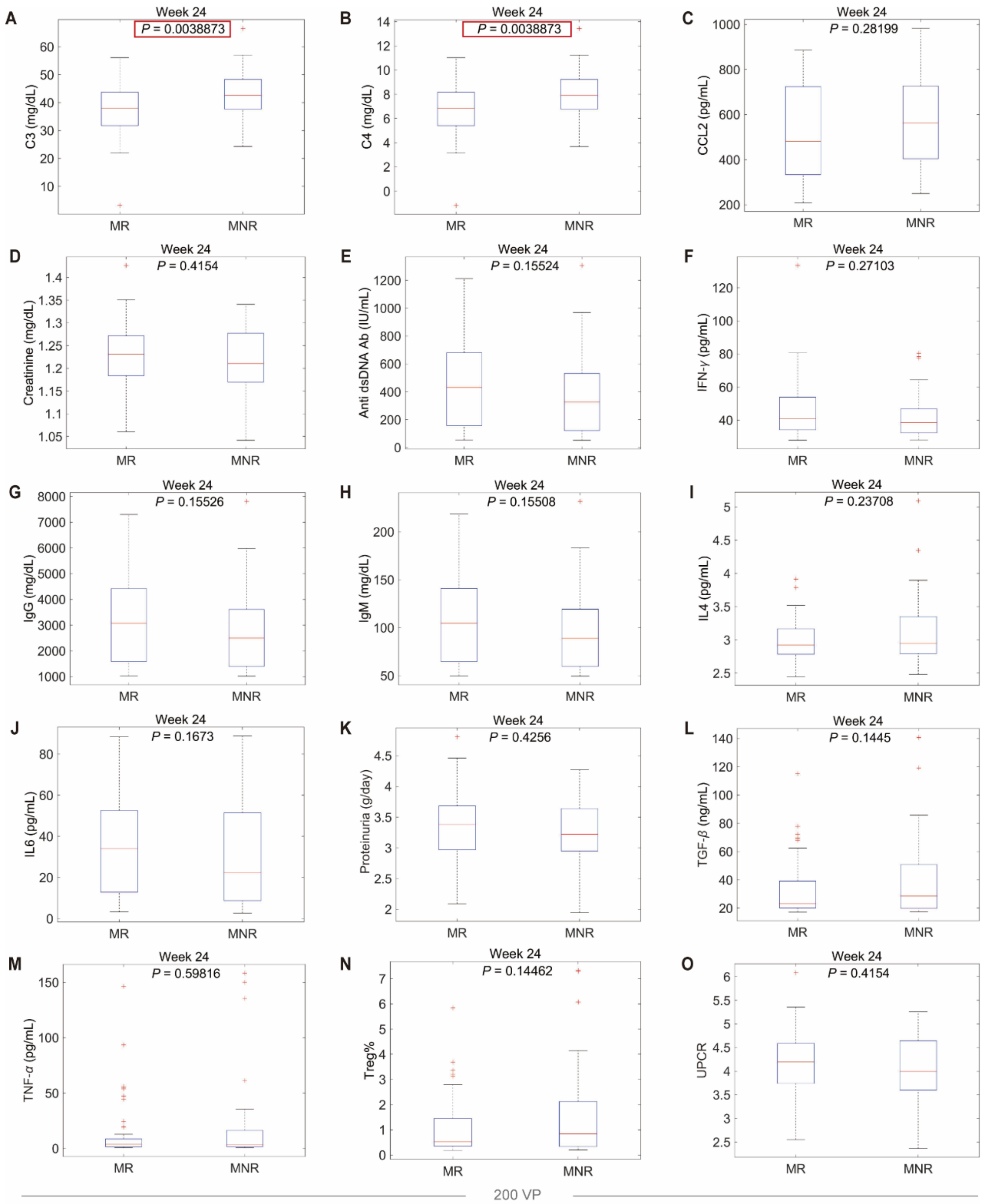

Figure S12 Model-based screening of potential pretreatment biomarkers in 24-week MSC plus SoC therapy–part 1. (A–O) Comparison of pre-treatment biomarker distributions in the responder (to MSC) subgroup and non-responder subgroup at week 24 using the selected virtual population (n = 200). The 25th, 50th, and 75th percentiles are depicted by boxplots with whiskers that define 1.5 times the interquartile range away from the bottom or top of the box. MR, responders to MSC therapy. MNR, non-responders to MSC therapy. *P*-values were calculated by *t*-test. The dosages of treatments are given as follows: mycophenolate mofetil (target total dose 2–2.5 g/day); methylprednisolone (a total of 1000–3000 mg intravenous); prednisone (initial prednisone dose 0.5 mg/kg/day, maximum 60 mg/day, with taper to 7.5 mg/day by week 12); MSC (single total dose of 1×10^8 cells, two doses infused at three-month intervals).

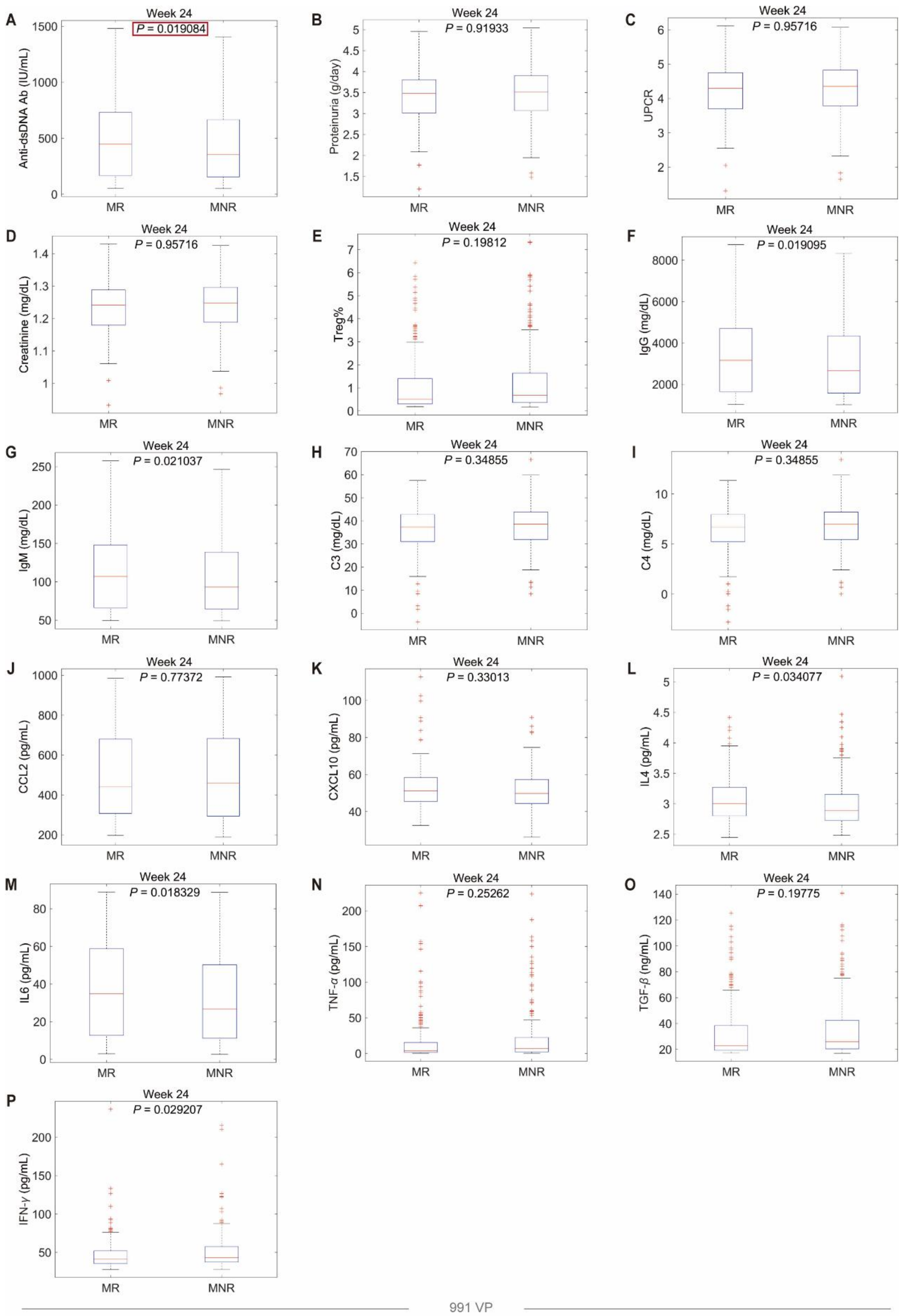

Figure S13 Model-based screening of potential pretreatment biomarkers in 24-week MSC plus SoC therapy–part 2. (A–P) Comparison of pre-treatment biomarker distributions in the responder (to MSC) subgroup and non-responder subgroup at week 24 using the full virtual population ($n = 991$). The 25th, 50th, and 75th percentiles are depicted by boxplots with whiskers that define 1.5 times

the interquartile range away from the bottom or top of the box. MR, responders to MSC therapy. MNR, non-responders to MSC therapy. *P*-values were calculated by *t*-test. The dosages of treatments are the same as in Fig. S12.

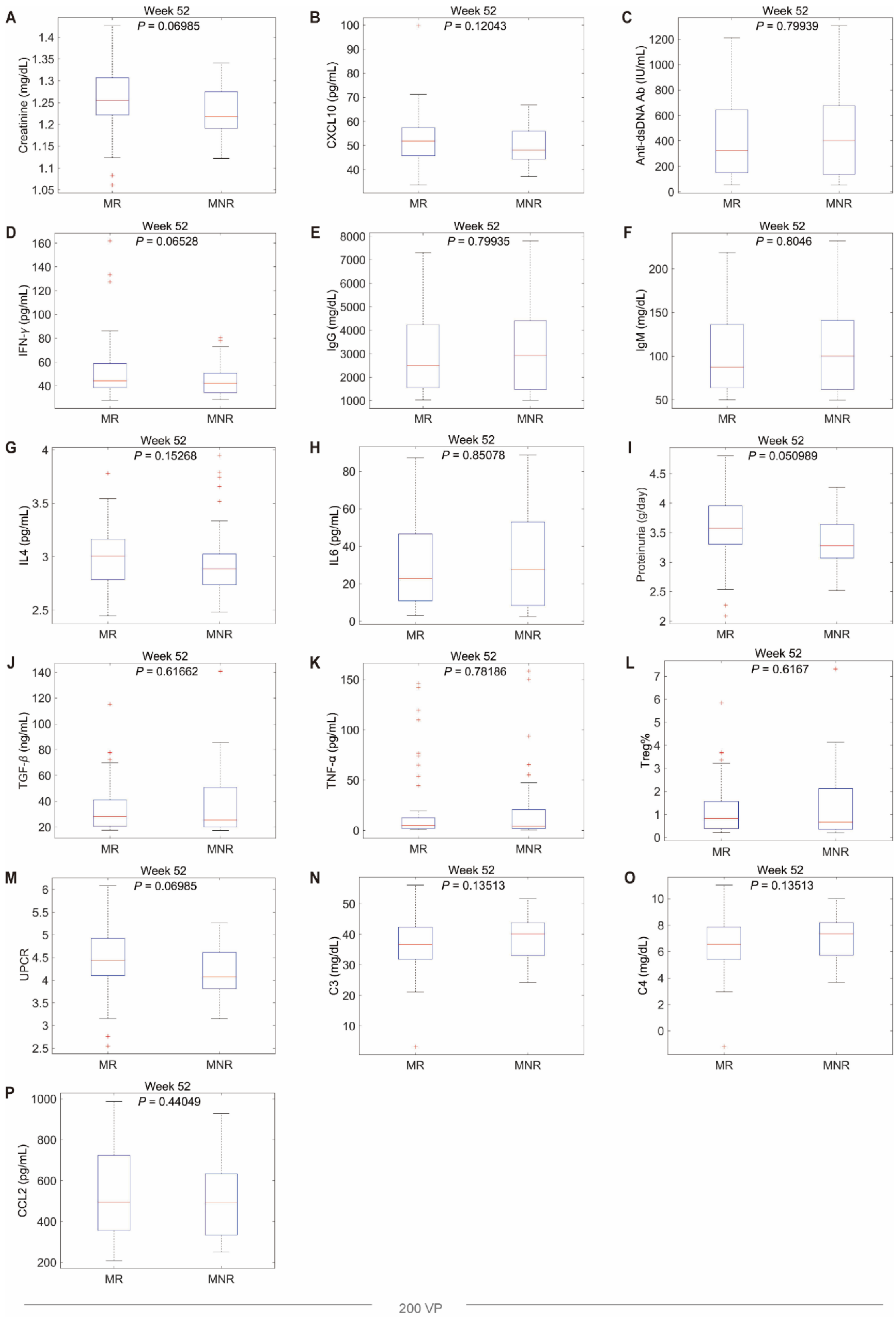

Figure S14 Model-based screening of potential pretreatment biomarkers in 52-week MSC plus SoC therapy–part 1. (A–P) Comparison of pre-treatment biomarker distributions in the responder (to MSC) subgroup and non-responder subgroup at week 52 using the selected virtual population (n = 200). The 25th, 50th, and 75th percentiles are depicted by boxplots with whiskers that define 1.5

times the interquartile range away from the bottom or top of the box. MR, responders to MSC therapy. MNR, non-responders to MSC therapy. *P*-values were calculated by *t*-test. The dosages of treatments are the same as in Fig. S12.

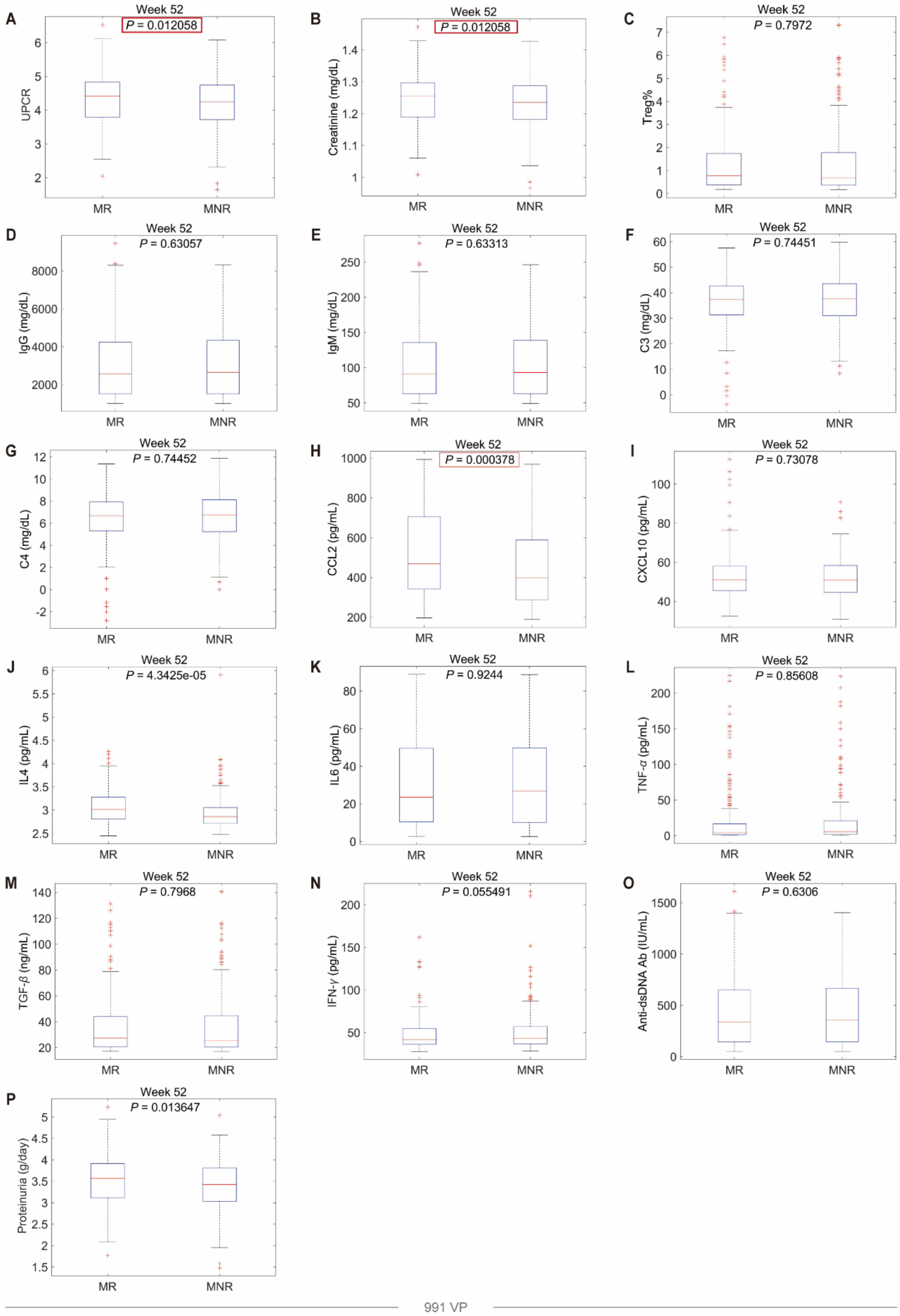

Figure S15 Model-based screening of potential pretreatment biomarkers in 52-week MSC plus SoC therapy–part 2. (A–P) Comparison of pre-treatment biomarker distributions in the responder (to MSC) subgroup and non-responder subgroup at week 52 using the full virtual population ($n = 991$). The 25th, 50th, and 75th percentiles are depicted by boxplots with whiskers that define 1.5 times

the interquartile range away from the bottom or top of the box. MR, responders to MSC therapy. MNR, non-responders to MSC therapy. *P*-values were calculated by *t*-test. The dosages of treatments are the same as in Fig. S12.

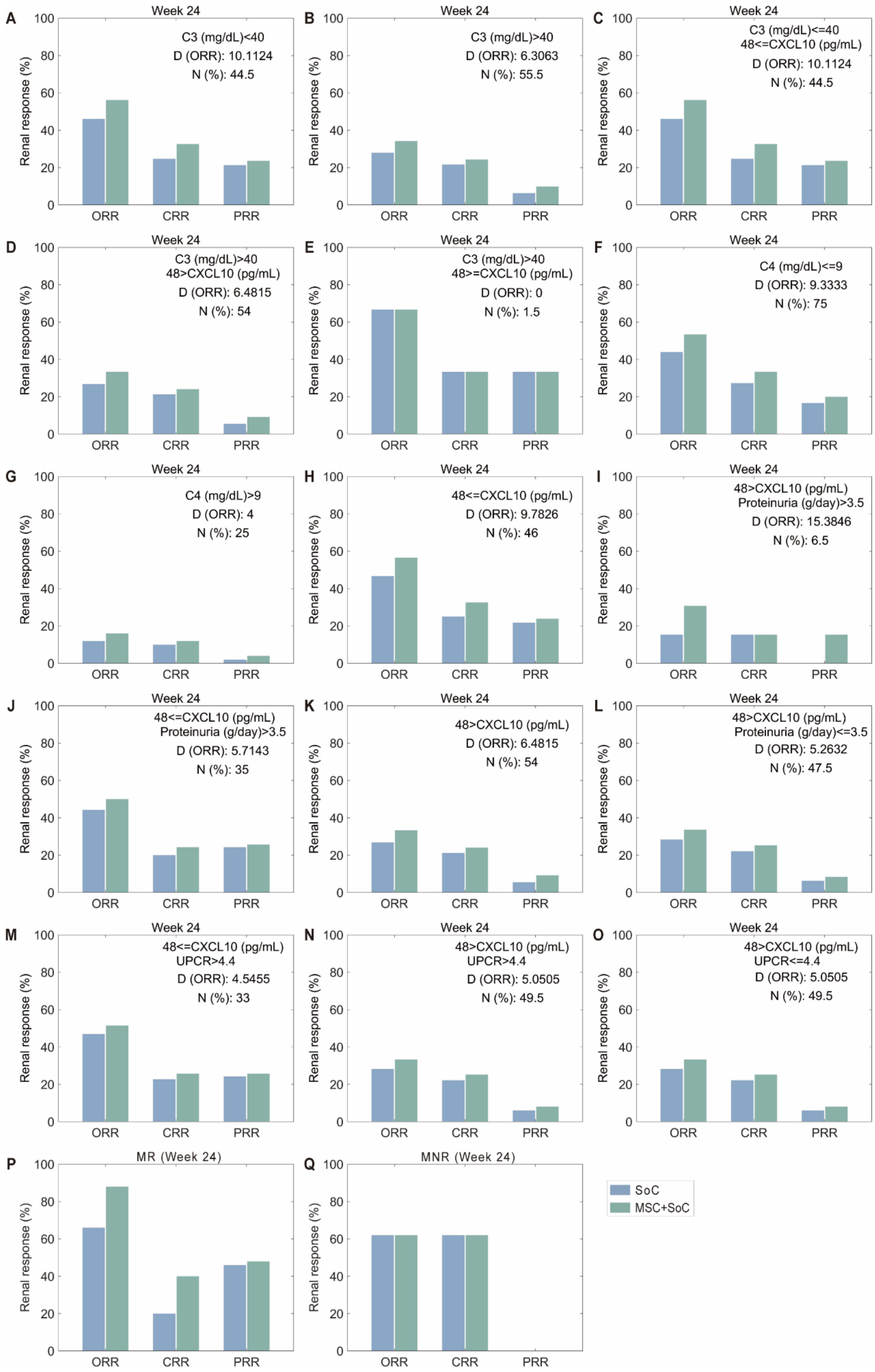
A
Week 24
C3 (mg/dL)<40
D (ORR): 10.1124
N (%): 44.5
B
Week 24
C3 (mg/dL)>40
D (ORR): 6.3063
N (%): 55.5
C
Week 24
C3 (mg/dL)<=40
48<=CXCL10 (pg/mL)
D (ORR): 10.1124
N (%): 44.5
D
Week 24
C3 (mg/dL)>40
48>CXCL10 (pg/mL)
D (ORR): 6.4815
N (%): 54
E
Week 24
C3 (mg/dL)>40
48>=CXCL10 (pg/mL)
D (ORR): 0
N (%): 1.5
F
Week 24
C4 (mg/dL)<=9
D (ORR): 9.3333
N (%): 75
G
Week 24
C4 (mg/dL)>9
D (ORR): 4
N (%): 25
H
Week 24
48<=CXCL10 (pg/mL)
D (ORR): 9.7826
N (%): 46
I
Week 24
48>CXCL10 (pg/mL)
Proteinuria (g/day)>3.5
D (ORR): 15.3846
N (%): 6.5
J
Week 24
48<=CXCL10 (pg/mL)
Proteinuria (g/day)>3.5
D (ORR): 5.7143
N (%): 35
K
Week 24
48>CXCL10 (pg/mL)
D (ORR): 6.4815
N (%): 54
L
Week 24
48>CXCL10 (pg/mL)
Proteinuria (g/day)<=3.5
D (ORR): 5.2632
N (%): 47.5
M
Week 24
48<=CXCL10 (pg/mL)
UPCR>4.4
D (ORR): 4.5455
N (%): 33
N
Week 24
48>CXCL10 (pg/mL)
UPCR>4.4
D (ORR): 5.0505
N (%): 49.5
O
Week 24
48>CXCL10 (pg/mL)
UPCR<=4.4
D (ORR): 5.0505
N (%): 49.5
P
MR (Week 24)
Q
MNR (Week 24)
Renal response (%)
ORR
CRR
PRR
SoC
MSC+SoC

Figure S16 Evaluation of biomarker-based patient stratification in 24-week MSC plus SoC therapy using virtual clinical trials—part 2. (A–O) Simulated 24-week population-level patient renal response rates under the MSC plus SoC regimen in biomarker-stratified patient subgroups. D(ORR), the renal response rate differences between SoC and MSC treatment scenarios. N (%), subgroup size compared to the original virtual patient population (in percentages). (P) Simulated 24-week patient renal response rates under the MSC plus SoC regimen in the top-ranked MSC responders. (Q) Simulated 24-week patient renal response rates under the MSC plus SoC regimen in the bottom-ranked MSC nonresponders. SoC dosages (similar to Ref. 54): mycophenolate mofetil (target total dose 2–2.5 g/day), methylprednisolone (a total of 1000–3000 mg intravenous), prednisone (initial prednisone dose 0.5 mg/kg/day, maximum 60 mg/day, with taper to 7.5 mg/day by week 12). MSCs dosages: single dose of 1×10^8 cells, two doses infused at three-month intervals. ORR, overall renal response; CRR, complete renal response; PRR, partial renal response.

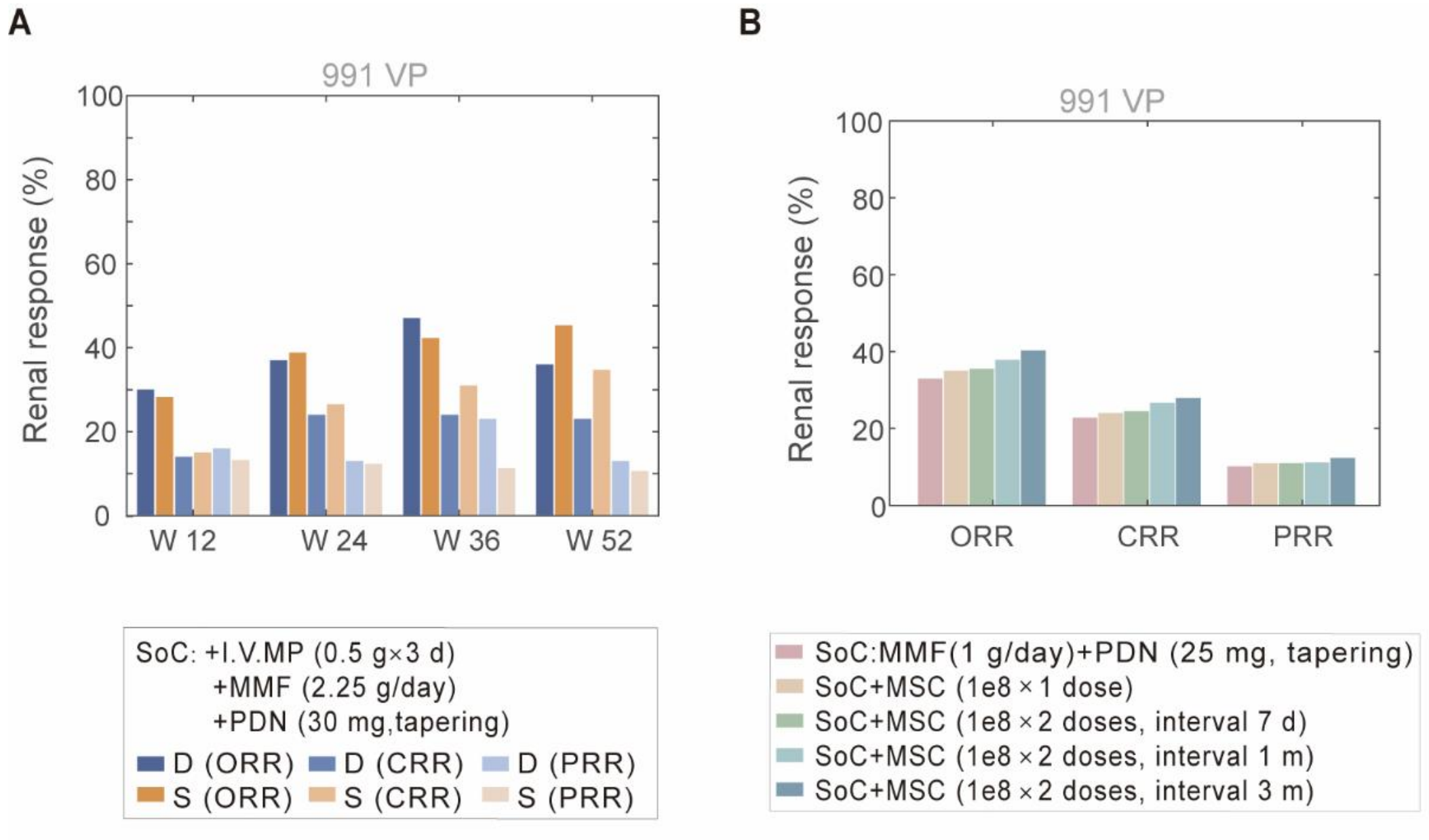

Figure S17 Clinical response prediction in an expanded LN virtual population. (A) Comparison of predicted versus observed population-level renal response rates in the full virtual patient population. The dosages of SoC treatments are similar to[54]: mycophenolate mofetil (target total dose 2–2.5 g/day) plus methylprednisolone (a total of 1000–3000 mg intravenous) plus prednisone (initial prednisone dose 0.5mg/kg/day, maximum 60 mg/day, with taper to 7.5 mg/day by week 12). (B) Simulated 24-week population-level renal response rates under different SoC and MSC dosing regimens using the full virtual patient population. The dosing regimens are the same as in Fig. S11. S, simulation; D, clinical data; ORR, overall renal response; CRR, complete renal response; PRR, partial renal response.

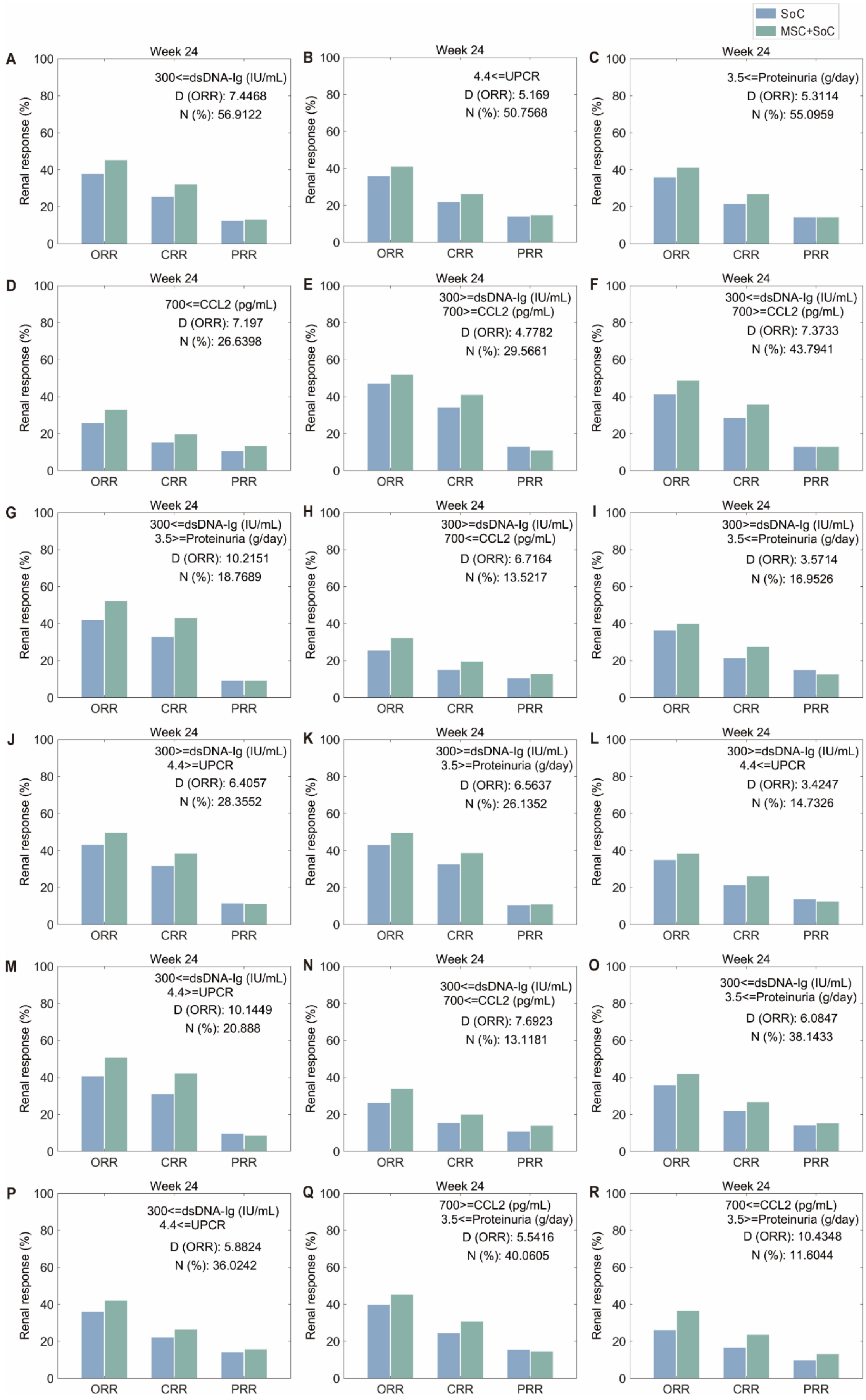

SoC
MSC+SoC
A
Week 24
300<=dsDNA-Ig (IU/mL)
D (ORR): 7.4468
N (%): 56.9122
B
Week 24
4.4<=UPCR
D (ORR): 5.169
N (%): 50.7568
C
Week 24
3.5<=Proteinuria (g/day)
D (ORR): 5.3114
N (%): 55.0959
D
Week 24
700<=CCL2 (pg/mL)
D (ORR): 7.197
N (%): 26.6398
E
Week 24
300>=dsDNA-Ig (IU/mL)
700>=CCL2 (pg/mL)
D (ORR): 4.7782
N (%): 29.5661
F
Week 24
300<=dsDNA-Ig (IU/mL)
700>=CCL2 (pg/mL)
D (ORR): 7.3733
N (%): 43.7941
G
Week 24
300<=dsDNA-Ig (IU/mL)
3.5>=Proteinuria (g/day)
D (ORR): 10.2151
N (%): 18.7689
H
Week 24
300>=dsDNA-Ig (IU/mL)
700<=CCL2 (pg/mL)
D (ORR): 6.7164
N (%): 13.5217
I
Week 24
300>=dsDNA-Ig (IU/mL)
3.5<=Proteinuria (g/day)
D (ORR): 3.5714
N (%): 16.9526
J
Week 24
300>=dsDNA-Ig (IU/mL)
4.4>=UPCR
D (ORR): 6.4057
N (%): 28.3552
K
Week 24
300>=dsDNA-Ig (IU/mL)
3.5>=Proteinuria (g/day)
D (ORR): 6.5637
N (%): 26.1352
L
Week 24
300>=dsDNA-Ig (IU/mL)
4.4<=UPCR
D (ORR): 3.4247
N (%): 14.7326
M
Week 24
300<=dsDNA-Ig (IU/mL)
4.4>=UPCR
D (ORR): 10.1449
N (%): 20.888
N
Week 24
300<=dsDNA-Ig (IU/mL)
700<=CCL2 (pg/mL)
D (ORR): 7.6923
N (%): 13.1181
O
Week 24
300<=dsDNA-Ig (IU/mL)
3.5<=Proteinuria (g/day)
D (ORR): 6.0847
N (%): 38.1433
P
Week 24
300<=dsDNA-Ig (IU/mL)
4.4<=UPCR
D (ORR): 5.8824
N (%): 36.0242
Q
Week 24
700>=CCL2 (pg/mL)
3.5<=Proteinuria (g/day)
D (ORR): 5.5416
N (%): 40.0605
R
Week 24
700<=CCL2 (pg/mL)
3.5>=Proteinuria (g/day)
D (ORR): 10.4348
N (%): 11.6044
Renal response (%)
0
20
40
60
80
100
ORR
CRR
PRR

Figure S18 Evaluation of biomarker-based patient stratification in 24-week MSC plus SoC therapy using virtual clinical trials—part 3. (A–R) Simulated 24-week population-level renal response rates under the MSC plus SoC regimen in biomarker-stratified patient subgroups (derived from the full virtual patient population of n = 991). D (ORR), the renal response rate differences between SoC and MSC treatment scenarios. N (%), subgroup size compared to the original virtual patient population (in percentages). The dosing regimens are the same as in Fig. S16.

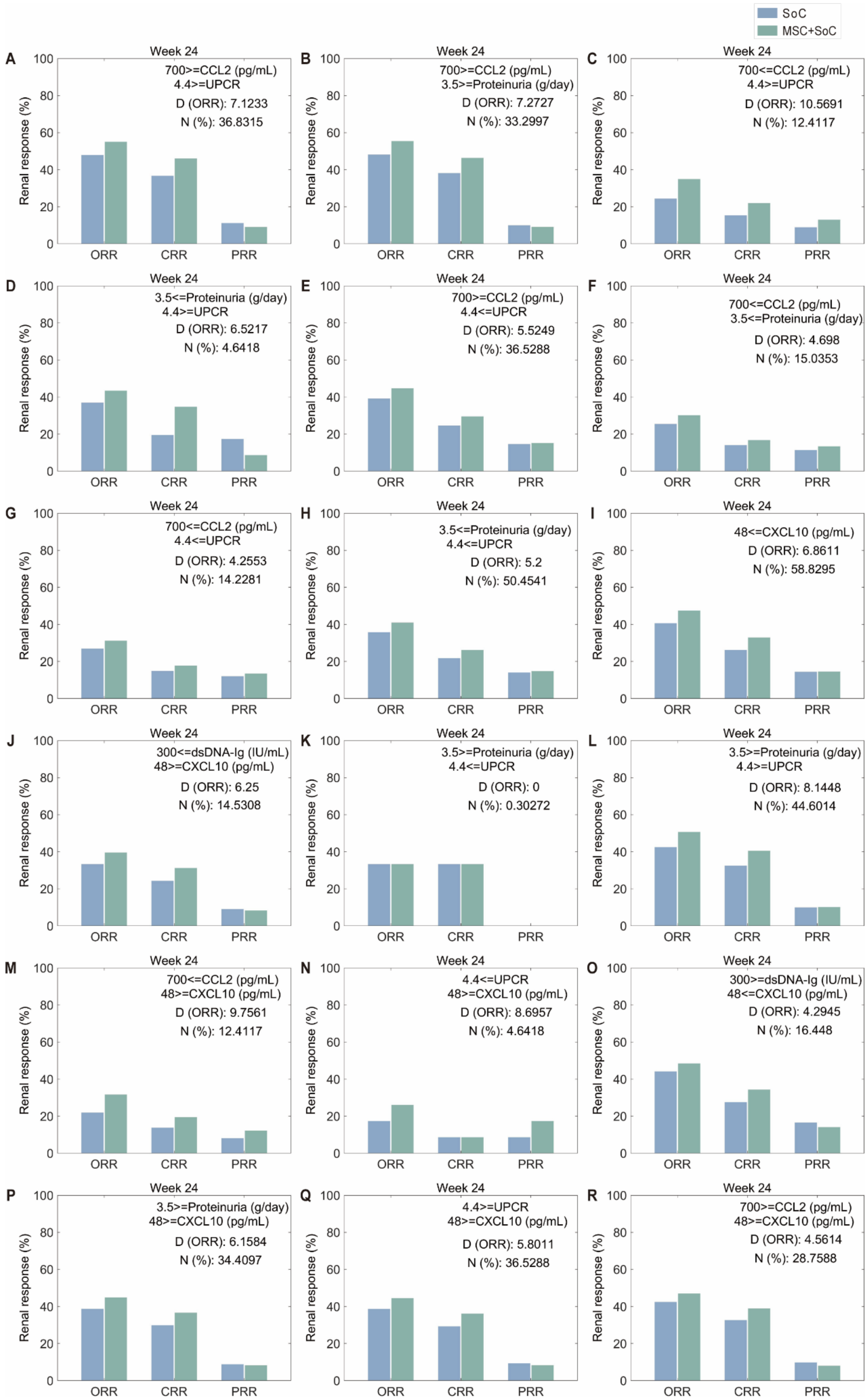
SoC
MSC+SoC
A Week 24
700>=CCL2 (pg/mL)
4.4>=UPCR
D (ORR): 7.1233
N (%): 36.8315
B Week 24
700>=CCL2 (pg/mL)
3.5>=Proteinuria (g/day)
D (ORR): 7.2727
N (%): 33.2997
C Week 24
700<=CCL2 (pg/mL)
4.4>=UPCR
D (ORR): 10.5691
N (%): 12.4117
D Week 24
3.5<=Proteinuria (g/day)
4.4>=UPCR
D (ORR): 6.5217
N (%): 4.6418
E Week 24
700>=CCL2 (pg/mL)
4.4<=UPCR
D (ORR): 5.5249
N (%): 36.5288
F Week 24
700<=CCL2 (pg/mL)
3.5<=Proteinuria (g/day)
D (ORR): 4.698
N (%): 15.0353
G Week 24
700<=CCL2 (pg/mL)
4.4<=UPCR
D (ORR): 4.2553
N (%): 14.2281
H Week 24
3.5<=Proteinuria (g/day)
4.4<=UPCR
D (ORR): 5.2
N (%): 50.4541
I Week 24
48<=CXCL10 (pg/mL)
D (ORR): 6.8611
N (%): 58.8295
J Week 24
300<=dsDNA-Ig (IU/mL)
48>=CXCL10 (pg/mL)
D (ORR): 6.25
N (%): 14.5308
K Week 24
3.5>=Proteinuria (g/day)
4.4<=UPCR
D (ORR): 0
N (%): 0.30272
L Week 24
3.5>=Proteinuria (g/day)
4.4>=UPCR
D (ORR): 8.1448
N (%): 44.6014
M Week 24
700<=CCL2 (pg/mL)
48>=CXCL10 (pg/mL)
D (ORR): 9.7561
N (%): 12.4117
N Week 24
4.4<=UPCR
48>=CXCL10 (pg/mL)
D (ORR): 8.6957
N (%): 4.6418
O Week 24
300>=dsDNA-Ig (IU/mL)
48<=CXCL10 (pg/mL)
D (ORR): 4.2945
N (%): 16.448
P Week 24
3.5>=Proteinuria (g/day)
48>=CXCL10 (pg/mL)
D (ORR): 6.1584
N (%): 34.4097
Q Week 24
4.4>=UPCR
48>=CXCL10 (pg/mL)
D (ORR): 5.8011
N (%): 36.5288
R Week 24
700>=CCL2 (pg/mL)
48>=CXCL10 (pg/mL)
D (ORR): 4.5614
N (%): 28.7588
Renal response (%)
0
20
40
60
80
100
ORR
CRR
PRR

Figure S19 Evaluation of biomarker-based patient stratification in 24-week MSC plus SoC therapy using virtual clinical trials—part 4. (A–R) Simulated 24-week population-level renal response rates under the MSC plus SoC regimen in biomarker-stratified patient subgroups (derived from the full virtual patient population of n = 991). D (ORR), the renal response rate differences between SoC and MSC treatment scenarios. N (%), subgroup size compared to the original virtual patient population (in percentages). The dosing regimens are the same as in Fig. S16.

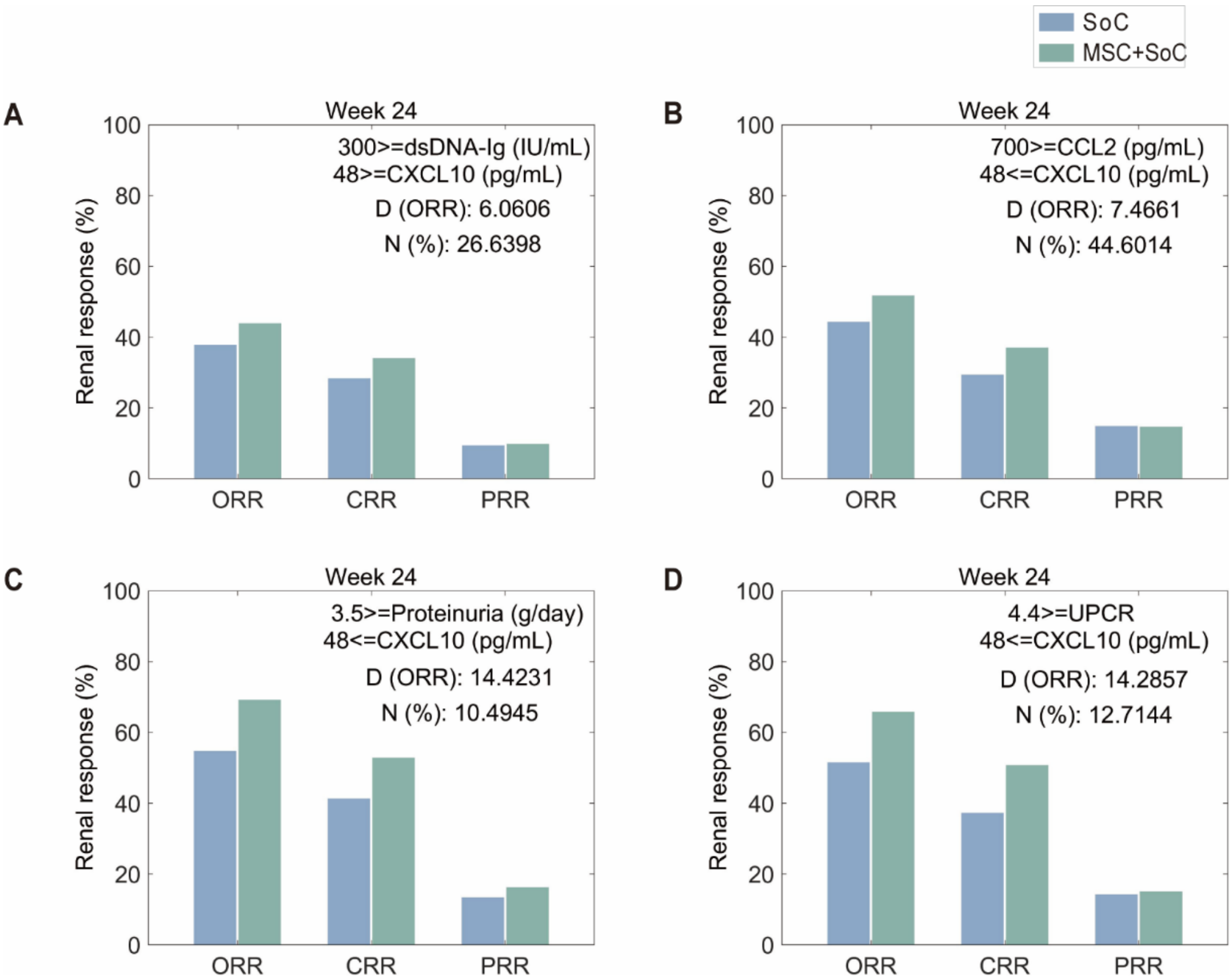

Figure S20 Evaluation of biomarker-based patient stratification in 24-week MSC plus SoC therapy using virtual clinical trials–part 5. (A–D) Simulated 24-week population-level renal response rates under the MSC plus SoC regimen in biomarker-stratified patient subgroups (derived from the full virtual patient population of n = 991). D (ORR), the renal response rate differences between SoC and MSC treatment scenarios. N (%), subgroup size compared to the original virtual patient population (in percentages). The dosing regimens are the same as in Fig. S16.

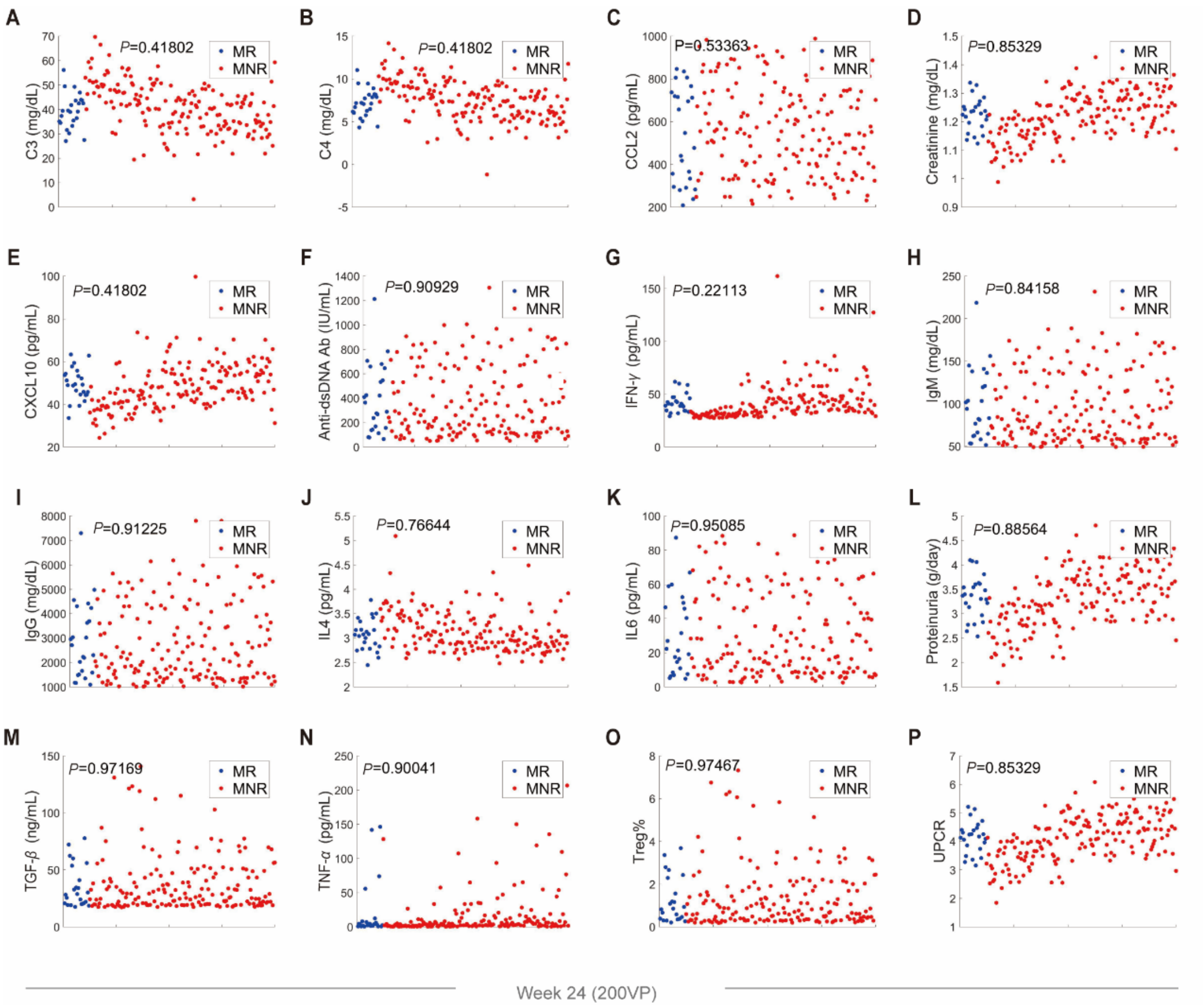

Figure S21 Pretreatment biomarker distribution of virtual patients in 24-week MSC plus SoC therapy. (A–P) Visualization of pre-treatment biomarker distribution profiles in the responder (to MSC) subgroup and non-responder subgroup at week 24 using the selected virtual population (n = 200). The distribution profiles are depicted by scatter plots with each dot indicating the biomarker value of an individual patient. The dosing regimens are the same as in Fig. S12. MR, responders to MSC therapy. MNR, non-responders to MSC therapy.

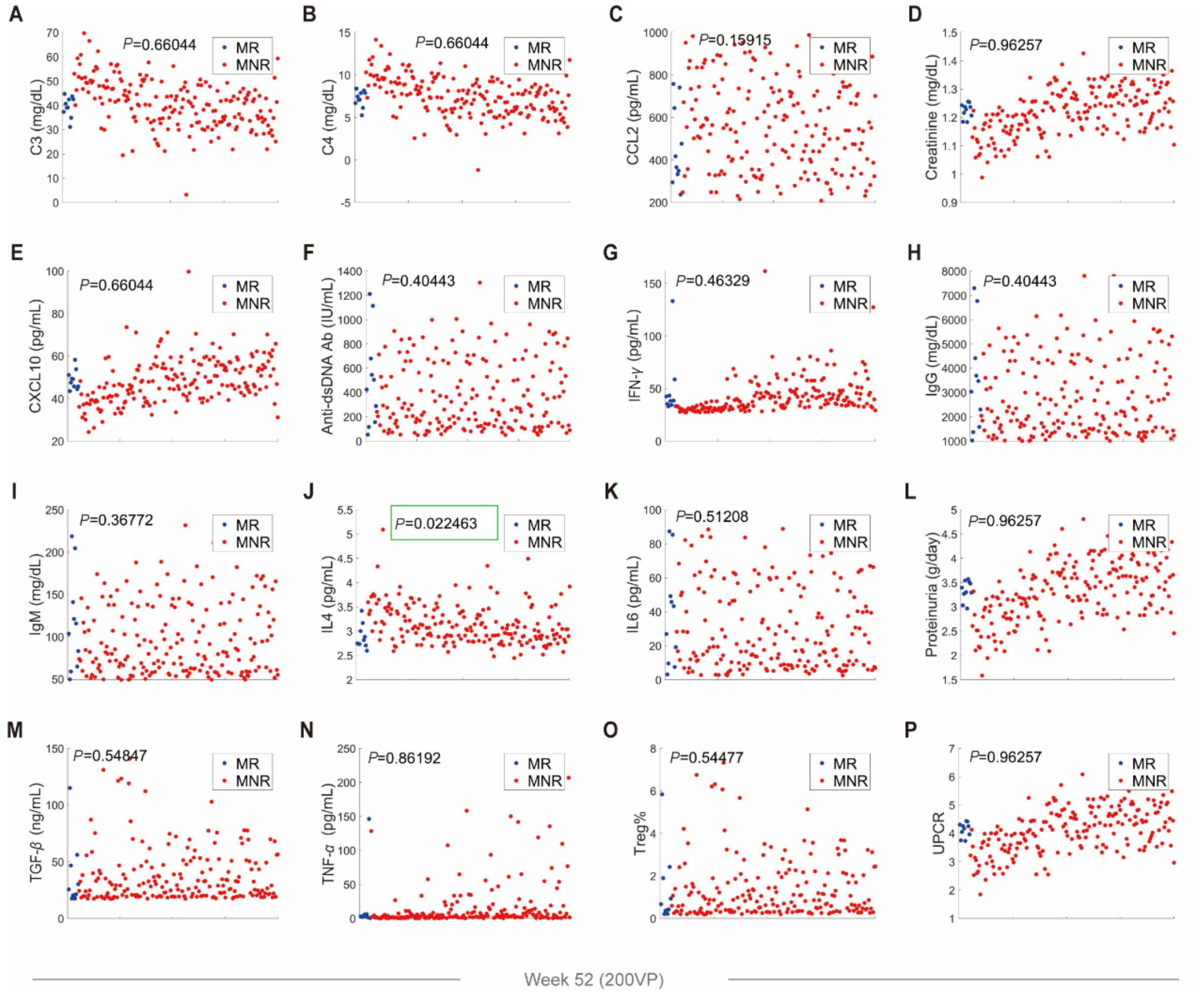

Figure S22 Pretreatment biomarker distribution of virtual patients in 52-week MSC plus SoC therapy. (A–P) Visualization of pre-treatment biomarker distribution profiles in the responder (to MSC) subgroup and non-responder subgroup at week 52 using the selected virtual population (n = 200). The distribution profiles are depicted by scatter plots with each dot indicating the biomarker value of an individual patient. The dosing regimens are the same as in Fig. S12. MR, responders to MSC therapy. MNR, non-responders to MSC therapy.

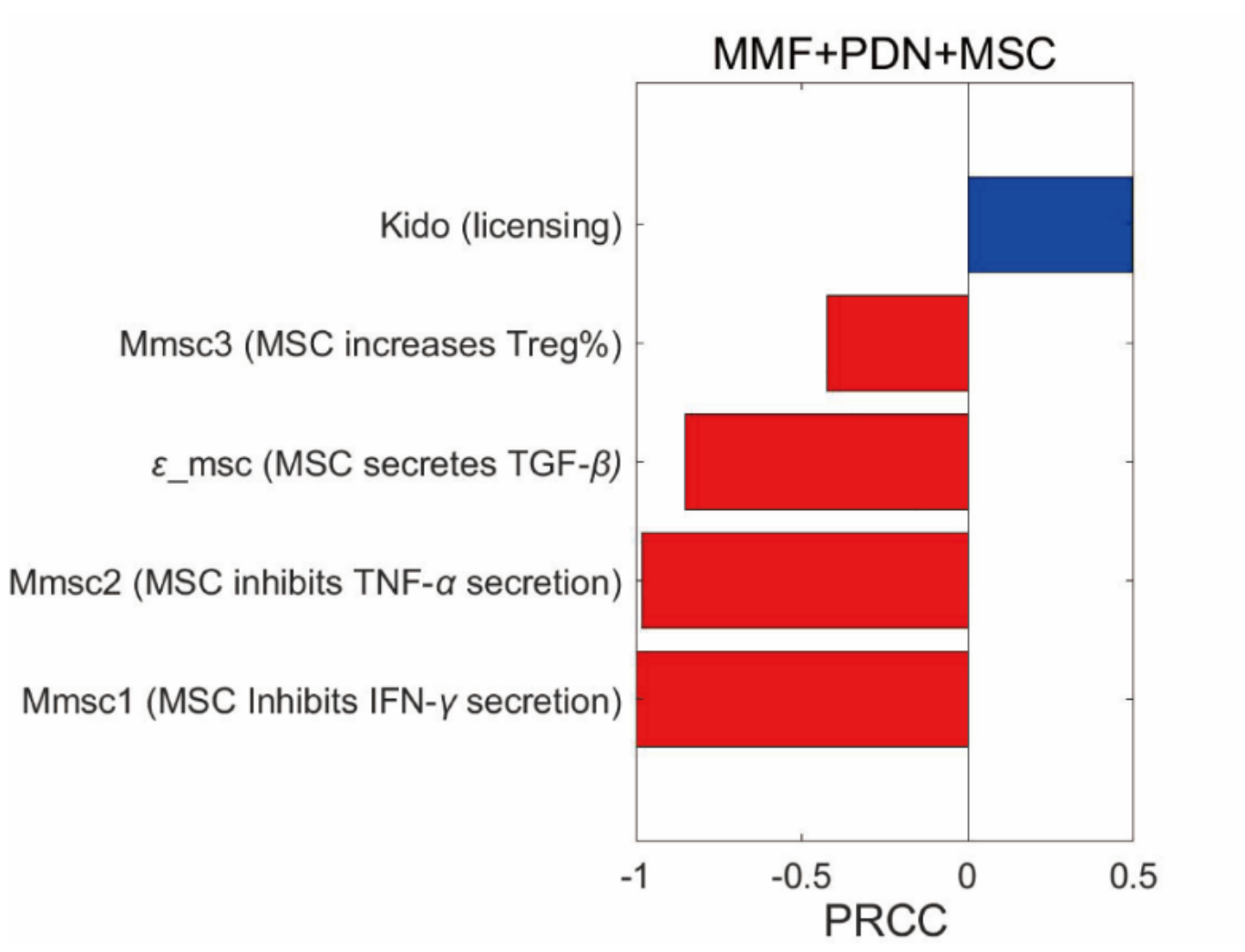

Figure S23 Global sensitivity analysis of MSC-specific modulatory parameters. Partial rank correlation coefficients (PRCC) indices of MSC-specific modulatory parameters using proteinuria as the output of interest, under MMF plus prednisone and MSC regimen. The positive or negative signs of PRCC values represent positive or negative effects of parameter increase on the model output.

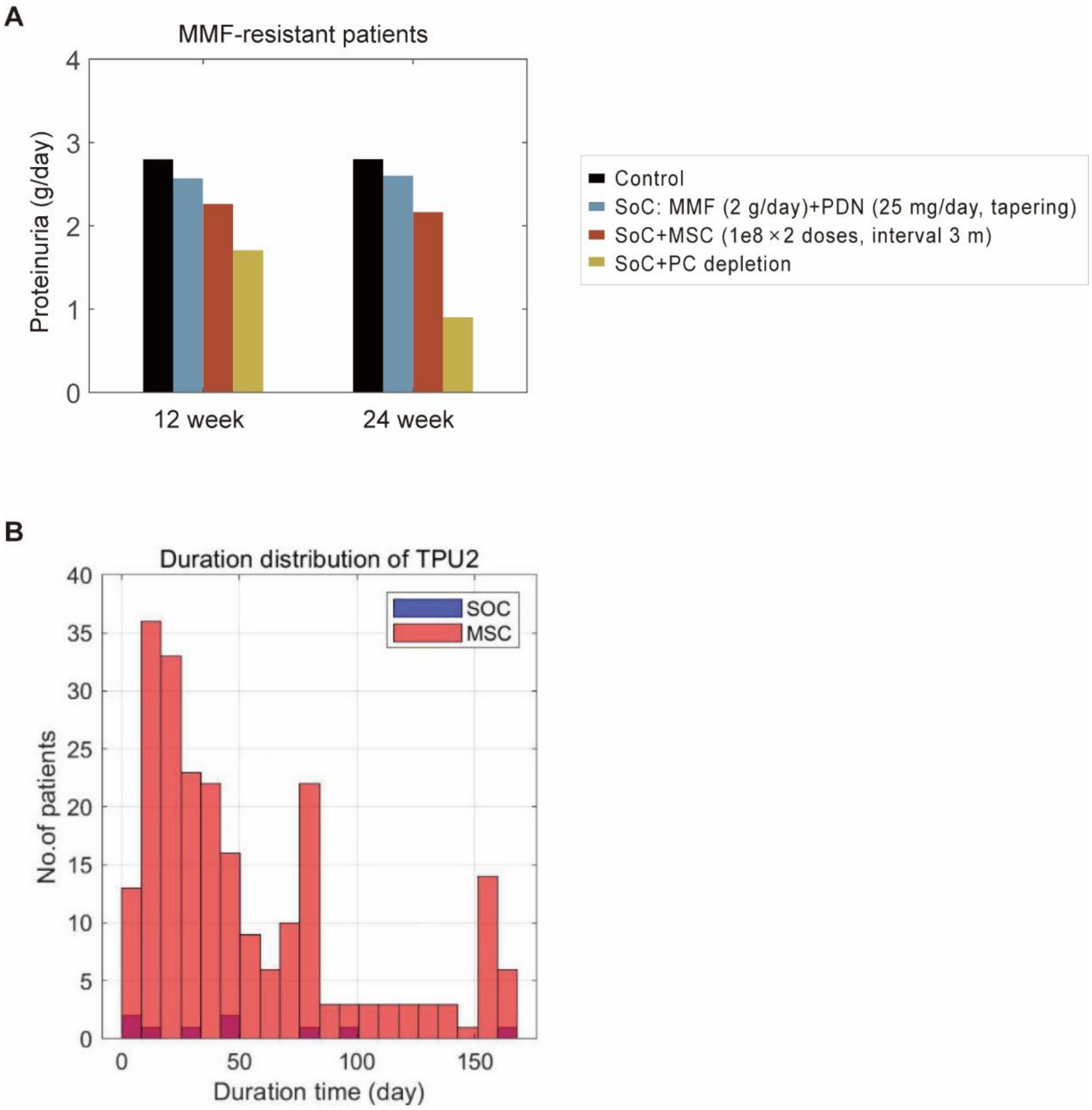

Figure S24 Predicted 24-week clinical response in MMF-resistant patients in response to different treatment strategies. (A) Simulated 24 h proteinuria response to different treatment strategies (including MSCs and plasma cell depletion) in a reference LN patient that is resistant to MMF-containing SoC regimens. SoC here consisted of mycophenolate mofetil (single target dose of 1 g, twice daily) combined with prednisone (initial dose of 25 mg, tapering). (B) In a larger virtual population resistant to SoC (*n* ~600), significantly more patients would be classified as TPU2 patients in the MSC+SoC arm compared to the SoC only arm (232 *vs* 9) with the simulated resulting TPU2 duration distribution, meaning that significantly more drug-resistant patients would experience treatment benefits (in terms of reduction in proteinuria to below 2) upon MSC treatment.

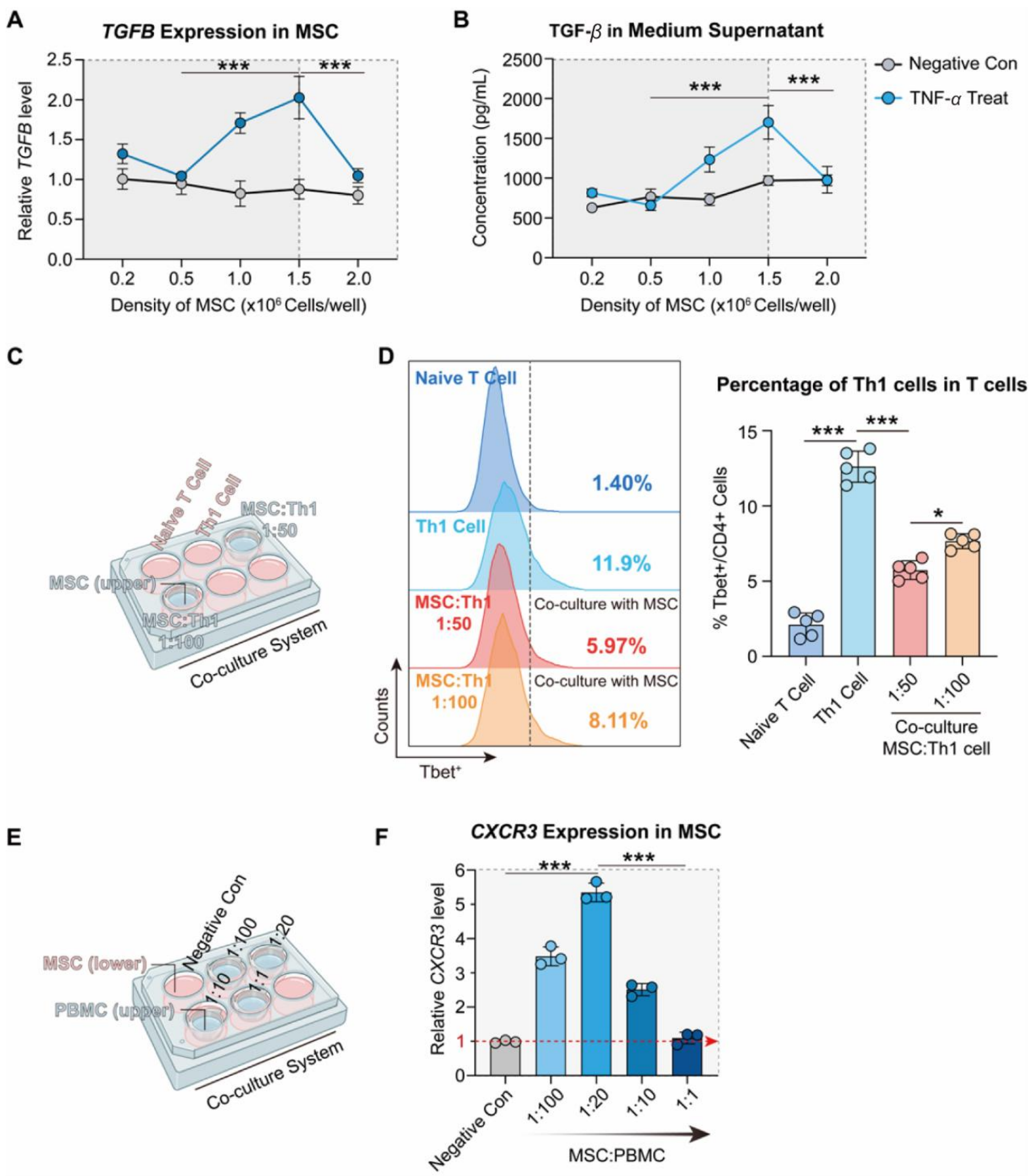

Figure S25 Effect of MSC concentrations on the immunomodulatory potential and *in vivo* homing ability of MSCs. (A, B) For MSCs at different concentrations, with or without TNF-α (100 ng/mL) stimulation for 12 h, (A) q-PCR was performed to assess TGFB expression in MSCs, (B) while ELISA was used to quantify TGFβ protein secretion levels in the corresponding culture supernatants (n = 6). The seeded cell density range was calculated proportional to the ratio of the culture plate surface area to the projected cell attachment area. To guarantee an adequate nutrient supply, the culture medium was supplemented at 1.5 times the standard volume required for normal cell growth. During the culture period, no adverse phenotypes such as cell-to-cell overlapping, morphological shrinkage, or floating dead cells were observed. Prior to sample collection at the experimental endpoint, cell viability was determined to be consistently above 95% using a trypan blue exclusion assay, thereby ensuring the reliability of the experimental data. (C) Schematic diagram of a non-contact MSC-Th1 cell co-culture system. (D) Upon MSC-Th1 co-culture, the percentage of Th1

cells in $CD4^+$ T cells was detected by flow cytometry. Th1 cells were labeled with Anti-Tbet Antibody ($n = 5$). (E) Schematic diagram of a non-contact MSC-PBMC co-culture system. (F) Upon MSC-PBMC co-culture, the expression level of CXCR3 in the obtained MSCs was investigated using q-PCR ($n = 3$). Data represent mean ± SD. Two-tailed unpaired equal-variance *t*-test. *$P < 0.05$ and ***$P < 0.001$, respectively.

2. Supporting tables

Table S1 Calculated reference cell amount values (in lymph nodes and kidneys) used in the LN disease module.

Cell	Healthy		Lupus nephritis	
	#/g of tissue (Lymph node)	#/g of tissue (Kidney)	#/g of tissue (Lymph node)	#/g of tissue (Kidney)
B cells	1.00E+07[116]	2.24E+03[117]	1.16E+07[118]	4.18E+04[117]
Plasma cell	2.04E+05[119*]	2.56E+03[120*]	1.05E+07[118]	8.14E+04[117*]
CD4$^+$ T	1.00E+07[116]	5.43E+04[121*]	1.89E+07[118]	1.46E+05[119*]
Th1 cells	2.00E+06[122,123*]	2.14E+04[124]	3.78E+06[122,123*]	8.84E+04[124]
Th2 cells	8.05E+05[125*]	8.61E+03[125*]	1.55E+06[125*]	3.63E+04[125*]
Treg cell	1.5E+06[116*]	1.20E+03[121*]	5.5E+05[126*]	4.40E+02[126*]
CD8$^+$ T	7.00E+06[116]	5.46E+04[121]	7.56E+06[118]	1.72E+05[119*]
CTL	3.50E+05[119*]	2.73E+03[119*]	1.13E+06[119*]	2.58E+04[119*]
Total Mø	–	7.20E+04[119]	–	1.09E+05[119]
Podocyte	–	2.34E+06[127,128]	–	1.43E+06[92]

*Represents an estimated value calculated from the ratio of specific immune cells to total immune cells in the cited literature.

Table S2 Calculated reference cytokine/biomarker secretion or production rates used in the LN disease module.

Cytokine	Secreting cell	Secretion rate (pg/cell/day)
IFN-*γ*	Th1	2.33E-3[129]
	CTL	1.23E-4[130]
TNF-*α*	Th1	2.30E-3[131]
	M*ø*	2.19E-3[132]
IL-6	Th1	5.00E-4[133]
	M*ø*	2.1E-4[134]
IL-4	Th2	2.80E-3[135]
TGF-*β*	Treg	3.49E-3[136]
	M*ø*	2.51E-5[137]
CCL2	M*ø*	1.13E-2[138]
CXCL10	M*ø*	4.05E-4[139]
C3	M*ø*	2.43E-2[140]
C4	M*ø*	9.04E-2[141]
IgM	PC	4E2 (assumed)
IgG	PC	5.8E5 (assumed)
Anti-dsDNA Ab	PC	0.2 (assumed, unitless)

Table S3 Reference clinical measurement values of efficacy biomarkers, circulating cytokines and biomolecules in healthy volunteers and LN patients.

Clinical index	Unit	Healthy	Lupus nephritis
Proteinuria	g/day	< 0.15[142]	[a] 4.1 ± 3.1[49]
UPCR	g/g	< 0.2	[a] 2.9 ± 2.5[54]
Creatinine	mg/dL	0.8–1.2[142]	[a] 1.06 ± 0.52[49]
IgM	mg/dL	42–300[143]	[a] 64 ± 46.13[56]
IgG	mg/dL	701–1622[143]	[a] 1520 ± 147.06[56]
Anti-dsDNA Ab	IU/mL	0–35[52]	[a] 168 ± 140.1[55]
C3	mg/dL	90–180[142]	[a] 69.6 ± 30.0[49]
C4	mg/dL	10–40[142]	[a] 13.2 ± 8.5[49]
IL-6	pg/mL	[b] 2.18(0.62–4.64) [109]	[b] 10.02(0.62-81.78)[109]
IL-4	pg/mL	[b] 2.66(1.22–3.05)[110]	[b] 2.9(0-3.36)[110]
TNF-α	pg/mL	[b] 0.55(0.55–4.29)[109]	[b] 1.24(0.55–12.51)[109]
IFN-γ	pg/mL	[c] 28.4(23.0–36.1)[111]	[c] 31.2(25.5–43.3)[111]
TGF-β	ng/dL	[b] 82.7(57.8–119.1)[109]	[b] 43(10.2–85.9)[109]
CCL2	pg/mL	[d] 322.6 ± 89.4[112]	[d] 516.1 ± 236.6[112]
Treg%	/	[c] 8.49(1.55–10.8)[115]	[c] 2.71(1.04–8.23)[115]
CXCL10	pg/mL	[e] 11.7[113]	[a] 32.98 ± 27.81[114]

[a] Mean ± standard deviations; [b] Median (range); [c] Median (25th percentile,75th percentile); [d] Mean ± SEM; [e] Median value.

Table S4 Physiological parameters used in the PBPK module.

Parameter (unit)	Mouse	Rat	Human
Body weight (kg)	0.02	0.25	60
Cardiac output (L/h/Kg$^{0.75}$)	16.5	15	12.9
Blood flow to organ (fraction of cardiac output, unitless)			
Lung	1	1	1
Liver	0.161	0.183	0.227
Spleen	0.011	0.011	0.011
Kidney	0.091	0.141	0.175
Heart	0.066	0.051	0.04
Rest of body	0.671	0.614	0.547
Organ volumes (fraction of body weight, unitless)			
Lung	0.007	0.005	0.014
Liver	0.055	0.037	0.026
Spleen	0.0035	0.002	0.0026
Kidney	0.017	0.007	0.0044
Heart	0.005	0.0033	0.0047
Blood	0.0085	0.074	0.079
Volume fraction of blood in organs (unitless)			
Lung	0.5	0.36	0.3
Liver	0.31	0.21	0.11
Spleen	0.17	0.22	0.5
Kidney	0.24	0.16	0.36
Heart	0.26	0.26	0.17
Rest of body	0.04	0.04	0.01

All values are taken from literature Ref. 144 and then optimized.

Table S5 MSC-specific parameters used in the PBPK module.

Parameter	Description	Blood	Lung	Liver	Spleen	Kidney	Heart	Rest
P (unitless)	Partition coefficients	–	740	260	1633	305	3	6.7
K_{arrest} (h^{-1})	Arrest rate constant	–	5.434	1.4	0.6	1.73	1.251	0.14
$K_{release}$ (h^{-1})	Release rate constant	–	0.1	0.07	0.86	0.05	0.016	0.96
$K_{depletion}$ (h^{-1})	Depletion rate constant	0.636	0.06	0.06	0.002	0.15	0.04	0.15

Table S6. General trends of efficacy markers upon MSC treatment as observed in published trials.

Disease	Proteinuria	Creatinine	C3	Treg%	Anti-dsDNA Ab
SLE[41]				↑	
SLE[145]	↓			↑	
SLE[146]	↓			↑	
SLE[147]	↓	↓	↑		↓
SLE[148]	↓	↓	↑		↓
SLE[149#]	↓	↓	↑		↓
SLE[150]		↓			↓

MSCs were given in two infusion doses at 7-day intervals; all other trials performed one MSC infusions; patient response shown here in the SLE setting were used as qualitative references for the evaluation of our lupus nephritis QSP model performance.

Table S7 Description of top-ranked sensitive model parameters.

Parameter (unitless)	Description
$\delta 4$	Rate of plasma cell apoptosis
Km54	CCL2 secretion inhibition by Pred
$\alpha 8$	Rate of podocytes differentiation
$\delta 1$	Rate of CD4/Th1/Th2/CD8 apoptosis
Km41	T/B proliferation inhibition by MPA
$\alpha 3$	Rate of Treg differentiation
Km42	CXCL10 secretion inhibition by Pred
Km51	IFN-γ secretion inhibition by Pred
$\delta 5$	Rate of CTL apoptosis
$\gamma 1$	Rate of Th1 proliferation
$\beta 1$	Rate of $CD4^+$ T proliferation
$\delta 3$	Rate of Treg apoptosis
$\delta 8$	Rate of podocytes apoptosis

Table S8 Proposed MoAs of SoC therapeutics and MSCs in the integrative QSP model.

Therapeutics	Mechanisms of action	Ref.
Mesenchymal stem cells (MSCs)	(1) Inhibits IFN-γ and TNF-α secretion by T cells.	47
	(2) CXCL10 promotes MSC migration.	39
	(3) MSC secretes TGF-β.	40
	(4) MSCs secrete TGF-β to rebalance the abnormal Treg cell ratio.	41,42,151
	(5) MSCs require IFN-γ "licensing" to secrete IDO to perform their immunosuppressive function.	43-46
Mycophenolate mofetil (MMF)	(1) Inhibits proliferation of T and B cells.	26
	(2) Induces apoptosis of T cells.	28
	(3) Inhibits Treg differentiation.	27
	(4) Inhibits antibody secretion by plasma cells.	29
Prednisolone	(1) Induces apoptosis of T-cell subsets.	30
	(2) Inhibits antibody secretion by plasma cells.	25
	(3) Inhibits cytokines secretion by PBMC cells (IL-6, IFN-γ, TNF-α, CCL2, CXCL10).	31-35
cyclophosphamide (CTX)	(1) Induces apoptosis of T cells.	36
	(2) Inhibits IgG synthesis.	37

References

1 Wigren M, Nilsson J, Kaplan MJ. Pathogenic immunity in systemic lupus erythematosus and atherosclerosis: common mechanisms and possible targets for intervention. *J Intern Med* 2015;**278**:494-506.

2 Agrewala JN, Brown DM, Lepak NM, Duso D, Huston G, Swain SL. Unique ability of activated CD4+ T cells but not rested effectors to migrate to non-lymphoid sites in the absence of inflammation. *J Biol Chem* 2007;**282**:6106-15.

3 Tesch GH, Maifert S, Schwarting A, Rollins BJ, Kelley VR. Monocyte chemoattractant protein 1-dependent leukocytic infiltrates are responsible for autoimmune disease in MRL-Fas(lpr) mice. *J Exp Med* 1999;**190**:1813-24.

4 Xie H, Lim YC, Luscinskas FW, Lichtman AH. Acquisition of selectin binding and peripheral homing properties by CD4(+) and CD8(+) T cells. *J Exp Med* 1999;**189**:1765-76.

5 McGeachy MJ, Cua DJ. Th17 cell differentiation: the long and winding road. *Immunity* 2008;**28**:445-53.

6 Figueiredo AS, Schumacher A. The T helper type 17/regulatory T cell paradigm in pregnancy. *Immunology* 2016;**148**:13-21.

7 Jiang D, Liang J, Guo R, Xie T, Kelly FL, Martinu T, et al. Long-term exposure of chemokine CXCL10 causes bronchiolitis-like inflammation. *Am J Respir Cell Mol Biol* 2012;**46**:592-8.

8 Diehl SA, Schmidlin H, Nagasawa M, Blom B, Spits H. IL-6 triggers IL-21 production by human CD4+ T cells to drive STAT3-dependent plasma cell differentiation in B cells. *Immunol Cell Biol* 2012;**90**:802-11.

9 Hiepe F, Radbruch A. Plasma cells as an innovative target in autoimmune disease with renal manifestations. *Nat Rev Nephrol* 2016;**12**:232-40.

10 Espeli M, Bökers S, Giannico G, Dickinson HA, Bardsley V, Fogo AB, et al. Local renal autoantibody production in lupus nephritis. *J Am Soc Nephrol* 2011;**22**:296-305.

11 Liu RX, Wei Y, Zeng QH, Chan KW, Xiao X, Zhao XY, et al. Chemokine (C-X-C motif) receptor 3-positive B cells link interleukin-17 inflammation to protumorigenic macrophage polarization in human hepatocellular carcinoma. *Hepatology* 2015;**62**:1779-90.

12 Genin M, Clement F, Fattaccioli A, Raes M, Michiels C. M1 and M2 macrophages derived from THP-1 cells differentially modulate the response of cancer cells to etoposide. *BMC Cancer* 2015;**15**:577.

13 Luo XY, Takahara T, Kawai K, Fujino M, Sugiyama T, Tsuneyama K, et al. IFN-γ deficiency attenuates hepatic inflammation and fibrosis in a steatohepatitis model induced by a methionine- and choline-deficient high-fat diet. *Am J Physiol Gastrointest Liver Physiol* 2013;**305**:G891-9.

14 Chen W, Frank ME, Jin W, Wahl SM. TGF-beta released by apoptotic T cells contributes to an immunosuppressive milieu. *Immunity* 2001;**14**:715-25.

15 Casella G, Garzetti L, Gatta AT, Finardi A, Maiorino C, Ruffini F, et al. IL4 induces IL6-producing M2 macrophages associated to inhibition of neuroinflammation in vitro and in vivo. *J Neuroinflammation* 2016;**13**:139.

16 López-Hernández FJ, López-Novoa JM. Role of TGF-β in chronic kidney disease: an integration of tubular, glomerular and vascular effects. *Cell Tissue Res* 2012;**347**:141-54.

17 Kwant LE, Vegting Y, Tsang ASMWP, Kwakernaak AJ, Vogt L, Voskuyl AE, et al. Macrophages in Lupus Nephritis: Exploring a potential new therapeutic avenue. *Autoimmun Rev* 2022;**21**:103211.

18 Förger F, Matthias T, Oppermann M, Becker H, Helmke K. Clinical significance of anti-dsDNA antibody isotypes: IgG/IgM ratio of anti-dsDNA antibodies as a prognostic marker for lupus nephritis. *Lupus* 2004;**13**:36-44.

19 de Zubiria Salgado A, Herrera-Diaz C. Lupus nephritis: an overview of recent findings. *Autoimmune Dis* 2012;**2012**:849684.

20 Steiger S, Anders HJ. Interferon blockade in lupus: effects on antiviral immunity. *Nat Rev Nephrol* 2022;**18**:415-6.

21 Migliorini A, Anders HJ. A novel pathogenetic concept-antiviral immunity in lupus nephritis. *Nat Rev Nephrol* 2012;**8**:183-9.

22 Gasparotto M, Gatto M, Binda V, Doria A, Moroni G. Lupus nephritis: clinical presentations and outcomes in the 21st century. *Rheumatology (Oxford)* 2020;**59**:v39-v51.

23 Jiao Z, Ding JJ, Shen J, Liang HQ, Zhong LJ, Wang Y, et al. Population pharmacokinetic modelling for enterohepatic circulation of mycophenolic acid in healthy Chinese and the influence of polymorphisms in UGT1A9. *Br J Clin Pharmacol* 2008;**65**:893-907.

24 Kochappan R, Cao E, Han S, Hu L, Quach T, Senyschyn D, et al. Targeted delivery of mycophenolic acid to the mesenteric lymph node using a triglyceride mimetic prodrug approach enhances gut-specific immunomodulation in mice. *J Control Release* 2021;**332**:636-51.

25 Haneda M, Owaki M, Kuzuya T, Iwasaki K, Miwa Y, Kobayashi T. Comparative analysis of drug action on B-cell proliferation and differentiation for mycophenolic acid, everolimus, and prednisolone. *Transplantation* 2014;**97**:405-12.

26 Allison AC, Eugui EM. Mycophenolate mofetil and its mechanisms of action. *Immunopharmacology* 2000;**47**:85-118.

27 Prémaud A, Rousseau A, Johnson G, Canivet C, Gandia P, Muscari F, et al. Inhibition of T-cell activation and proliferation by mycophenolic acid in patients awaiting liver transplantation: PK/PD relationships. *Pharmacol Res* 2011;**63**:432-8.

28 Tang Q, Yang Y, Zhao M, Liang G, Wu H, Liu Q, et al. Mycophenolic acid upregulates miR-142-3P/5P and miR-146a in lupus CD4+T cells. *Lupus* 2015;**24**:935-42.

29 Matz M, Lehnert M, Lorkowski C, Fabritius K, Unterwalder N, Doueiri S, et al. Effects of sotrastaurin, mycophenolic acid and everolimus on human B-lymphocyte function and activation. *Transpl Int* 2012;**25**:1106-16.

30 Lanza L, Scudeletti M, Puppo F, Bosco O, Peirano L, Filaci G, et al. Prednisone increases apoptosis in in vitro activated human peripheral blood T lymphocytes. *Clin Exp Immunol* 1996;**103**:482-90.

31 Noack M, Ndongo-Thiam N, Miossec P. Evaluation of Anti-inflammatory Effects of Steroids and Arthritis-Related Biotherapies in an In Vitro Coculture Model with

Immune Cells and Synoviocytes. *Front Immunol* 2016;**7**:509.
32 Correale J, Arias M, Gilmore W. Steroid hormone regulation of cytokine secretion by proteolipid protein-specific CD4+ T cell clones isolated from multiple sclerosis patients and normal control subjects. *J Immunol* 1998;**161**:3365-74.
33 Okoye IS, Xu L, Walker J, Elahi S. The glucocorticoids prednisone and dexamethasone differentially modulate T cell function in response to anti-PD-1 and anti-CTLA-4 immune checkpoint blockade. *Cancer Immunol Immunother* 2020;**69**:1423-36.
34 Gauthier M, Chakraborty K, Oriss TB, Raundhal M, Das S, Chen J, et al. Severe asthma in humans and mouse model suggests a CXCL10 signature underlies corticosteroid-resistant Th1 bias. *JCI Insight* 2017;**2**:e94580.
35 Kim BY, Son Y, Kim MS, Kim K. Prednisolone suppresses the immunostimulatory effects of 27-hydroxycholesterol. *Exp Ther Med* 2020;**19**:2335-42.
36 Strauss G, Westhoff MA, Fischer-Posovszky P, Fulda S, Schanbacher M, Eckhoff SM, et al. 4-hydroperoxy-cyclophosphamide mediates caspase-independent T-cell apoptosis involving oxidative stress-induced nuclear relocation of mitochondrial apoptogenic factors AIF and EndoG. *Cell Death Differ* 2008;**15**:332-43.
37 Kodo H, Bonavida B, Colvin M, Gale RP. Dose-response effects of 4-hydroperoxycyclophosphamide on human T and B cell function in vitro. *Int J Immunopharmacol* 1985;**7**:555-60.
38 Wang H, Liang X, Xu ZP, Crawford DH, Liu X, Roberts MS. A physiologically based kinetic model for elucidating the in vivo distribution of administered mesenchymal stem cells. *Sci Rep* 2016;**6**:22293.
39 Lee HK, Kim KH, Kim HS, Kim JS, Lee JH, Ji A, et al. Effect of a Combination of Prednisone or Mycophenolate Mofetil and Mesenchymal Stem Cells on Lupus Symptoms in MRL.Fas(lpr) Mice. *Stem Cells Int* 2018;**2018**:4273107.
40 Kim JH, Jo CH, Kim HR, Hwang YI. Comparison of Immunological Characteristics of Mesenchymal Stem Cells from the Periodontal Ligament, Umbilical Cord, and Adipose Tissue. *Stem Cells Int* 2018;**2018**:8429042.
41 Wang D, Huang S, Yuan X, Liang J, Xu R, Yao G, et al. The regulation of the Treg/Th17 balance by mesenchymal stem cells in human systemic lupus erythematosus. *Cell Mol Immunol* 2017;**14**:423-31.
42 Aggarwal S, Pittenger MF. Human mesenchymal stem cells modulate allogeneic immune cell responses. *Blood* 2005;**105**:1815-22.
43 Fibbe WE, Rabelink TJ. Lupus nephritis: Mesenchymal stromal cells in lupus nephritis. *Nat Rev Nephrol* 2017;**13**:452-3.
44 Boyt DT, Boland LK, Burand AJ, Jr., Brown AJ, Ankrum JA. Dose and duration of interferon γ pre-licensing interact with donor characteristics to influence the expression and function of indoleamine-2,3-dioxygenase in mesenchymal stromal cells. *J R Soc Interface* 2020;**17**:20190815.
45 Kim DS, Jang IK, Lee MW, Ko YJ, Lee DH, Lee JW, et al. Enhanced Immunosuppressive Properties of Human Mesenchymal Stem Cells Primed by Interferon-γ. *EBioMedicine* 2018;**28**:261-73.
46 François M, Romieu-Mourez R, Li M, Galipeau J. Human MSC suppression correlates with cytokine induction of indoleamine 2,3-dioxygenase and bystander M2

macrophage differentiation. *Mol Ther* 2012;**20**:187-95.
47 Zappia E, Casazza S, Pedemonte E, Benvenuto F, Bonanni I, Gerdoni E, et al. Mesenchymal stem cells ameliorate experimental autoimmune encephalomyelitis inducing T-cell anergy. *Blood* 2005;**106**:1755-61.
48 Spetie DN, Tang Y, Rovin BH, Nadasdy T, Nadasdy G, Pesavento TE, et al. Mycophenolate therapy of SLE membranous nephropathy. *Kidney Int* 2004;**66**:2411-5.
49 Ginzler EM, Dooley MA, Aranow C, Kim MY, Buyon J, Merrill JT, et al. Mycophenolate mofetil or intravenous cyclophosphamide for lupus nephritis. *N Engl J Med* 2005;**353**:2219-28.
50 Macallan DC, Wallace DL, Zhang Y, Ghattas H, Asquith B, de Lara C, et al. B-cell kinetics in humans: rapid turnover of peripheral blood memory cells. *Blood* 2005;**105**:3633-40.
51 Yap DY, Yung S, Ma MK, Mok MM, Kwan LP, Chan GC, et al. Serum immunoglobulin G level in patients with lupus nephritis and the effect of treatment with corticosteroids and mycophenolate mofetil. *Lupus* 2014;**23**:678-83.
52 Chan TM, Li FK, Tang CS, Wong RW, Fang GX, Ji YL, et al. Efficacy of mycophenolate mofetil in patients with diffuse proliferative lupus nephritis. Hong Kong-Guangzhou Nephrology Study Group. *N Engl J Med* 2000;**343**:1156-62.
53 Deng D, Zhang P, Guo Y, Lim TO. A randomised double-blind, placebo-controlled trial of allogeneic umbilical cord-derived mesenchymal stem cell for lupus nephritis. *Ann Rheum Dis* 2017;**76**:1436-9.
54 Furie RA, Aroca G, Cascino MD, Garg JP, Rovin BH, Alvarez A, et al. B-cell depletion with obinutuzumab for the treatment of proliferative lupus nephritis: a randomised, double-blind, placebo-controlled trial. *Ann Rheum Dis* 2022;**81**:100-7.
55 Moroni G, Raffiotta F, Trezzi B, Giglio E, Mezzina N, Del Papa N, et al. Rituximab vs mycophenolate and vs cyclophosphamide pulses for induction therapy of active lupus nephritis: a clinical observational study. *Rheumatology (Oxford)* 2014;**53**:1570-7.
56 Ginzler EM, Wax S, Rajeswaran A, Copt S, Hillson J, Ramos E, et al. Atacicept in combination with MMF and corticosteroids in lupus nephritis: results of a prematurely terminated trial. *Arthritis Res Ther* 2012;**14**:R33.
57 Gu F, Wang D, Zhang H, Feng X, Gilkeson GS, Shi S, et al. Allogeneic mesenchymal stem cell transplantation for lupus nephritis patients refractory to conventional therapy. *Clin Rheumatol* 2014;**33**:1611-9.
58 Wang J, Hu W, Xie H, Zhang H, Chen H, Zeng C, et al. Induction therapies for class IV lupus nephritis with non-inflammatory necrotizing vasculopathy: mycophenolate mofetil or intravenous cyclophosphamide. *Lupus* 2007;**16**:707-12.
59 Chan TM, Tse KC, Tang CS, Mok MY, Li FK. Long-term study of mycophenolate mofetil as continuous induction and maintenance treatment for diffuse proliferative lupus nephritis. *J Am Soc Nephrol* 2005;**16**:1076-84.
60 Sedhain A, Hada R, Agrawal RK, Bhattarai GR, Baral A. Low dose mycophenolate mofetil versus cyclophosphamide in the induction therapy of lupus nephritis in Nepalese population: a randomized control trial. *BMC Nephrol* 2018;**19**:175.
61 Li X, Ren H, Zhang Q, Zhang W, Wu X, Xu Y, et al. Mycophenolate mofetil or tacrolimus compared with intravenous cyclophosphamide in the induction treatment

for active lupus nephritis. *Nephrol Dial Transplant* 2012;**27**:1467-72.

62 El-Shafey EM, Abdou SH, Shareef MM. Is mycophenolate mofetil superior to pulse intravenous cyclophosphamide for induction therapy of proliferative lupus nephritis in Egyptian patients? *Clin Exp Nephrol* 2010;**14**:214-21.

63 Rivera F, Fulladosa X, Poveda R, Frutos MA, García-Frías P, Ara J, et al. Mycophenolate as induction therapy in lupus nephritis with renal function impairment. *Am J Nephrol* 2012;**35**:424-33.

64 Radhakrishnan J, Moutzouris DA, Ginzler EM, Solomons N, Siempos, II, Appel GB. Mycophenolate mofetil and intravenous cyclophosphamide are similar as induction therapy for class V lupus nephritis. *Kidney Int* 2010;**77**:152-60.

65 Appel GB, Contreras G, Dooley MA, Ginzler EM, Isenberg D, Jayne D, et al. Mycophenolate mofetil versus cyclophosphamide for induction treatment of lupus nephritis. *J Am Soc Nephrol* 2009;**20**:1103-12.

66 Furie R, Rovin BH, Houssiau F, Malvar A, Teng YKO, Contreras G, et al. Two-Year, Randomized, Controlled Trial of Belimumab in Lupus Nephritis. *N Engl J Med* 2020;**383**:1117-28.

67 Rovin BH, Teng YKO, Ginzler EM, Arriens C, Caster DJ, Romero-Diaz J, et al. Efficacy and safety of voclosporin versus placebo for lupus nephritis (AURORA 1): a double-blind, randomised, multicentre, placebo-controlled, phase 3 trial. *Lancet* 2021;**397**:2070-80.

68 Klinke DJ, 2nd. Integrating epidemiological data into a mechanistic model of type 2 diabetes: validating the prevalence of virtual patients. *Ann Biomed Eng* 2008;**36**:321-34.

69 Sové RJ, Jafarnejad M, Zhao C, Wang H, Ma H, Popel AS. QSP-IO: A Quantitative Systems Pharmacology Toolbox for Mechanistic Multiscale Modeling for Immuno-Oncology Applications. *CPT Pharmacometrics Syst Pharmacol* 2020;**9**:484-97.

70 Allen RJ, Rieger TR, Musante CJ. Efficient Generation and Selection of Virtual Populations in Quantitative Systems Pharmacology Models. *CPT Pharmacometrics Syst Pharmacol* 2016;**5**:140-6.

71 Schmidt BJ, Casey FP, Paterson T, Chan JR. Alternate virtual populations elucidate the type I interferon signature predictive of the response to rituximab in rheumatoid arthritis. *BMC Bioinformatics* 2013;**14**:221.

72 Xu H, Wang N, Cao W, Huang L, Zhou J, Sheng L. Influence of various medium environment to in vitro human T cell culture. *In Vitro Cell Dev Biol Anim* 2018;**54**:559-66.

73 Fike AJ, Nguyen LT, Kumova OK, Carey AJ. Characterization of CD31 expression on murine and human neonatal T lymphocytes during development and activation. *Pediatr Res* 2017;**82**:133-40.

74 Le Campion A, Bourgeois C, Lambolez F, Martin B, Léaument S, Dautigny N, et al. Naive T cells proliferate strongly in neonatal mice in response to self-peptide/self-MHC complexes. *Proc Natl Acad Sci U S A* 2002;**99**:4538-43.

75 MacPherson S, Keyes S, Kilgour MK, Smazynski J, Chan V, Sudderth J, et al. Clinically relevant T cell expansion media activate distinct metabolic programs uncoupled from cellular function. *Mol Ther Methods Clin Dev* 2022;**24**:380-93.

76 Olesch C, Sirait-Fischer E, Berkefeld M, Fink AF, Susen RM, Ritter B, et al. S1PR4 ablation reduces tumor growth and improves chemotherapy via CD8+ T cell expansion. *J Clin Invest* 2020;**130**:5461-76.

77 Ulbar F, Villanova I, Giancola R, Baldoni S, Guardalupi F, Fabi B, et al. Clinical-Grade Expanded Regulatory T Cells Are Enriched with Highly Suppressive Cells Producing IL-10, Granzyme B, and IL-35. *Biol Blood Marrow Transplant* 2020;**26**:2204-10.

78 Guo H, Xun L, Zhang R, Hu F, Luan J, Lao K, et al. Stability and inhibitory function of Treg cells under inflammatory conditions in vitro. *Exp Ther Med* 2019;**18**:2443-50.

79 Zou B, Xia S, Du X, Xu Y, Ning N, Li S, et al. Treatment Effect of Tuftsin and Antigen Peptide Combined with Immune Cells on Colorectal Cancer. *Med Sci Monit* 2019;**25**:5465-72.

80 Yoon SH, Cho HI, Kim TG. Activation of B cells using Schneider 2 cells expressing CD40 ligand for the enhancement of antigen presentation in vitro. *Exp Mol Med* 2005;**37**:567-74.

81 Rousset F, Peyrol S, Garcia E, Vezzio N, Andujar M, Grimaud JA, et al. Long-term cultured CD40-activated B lymphocytes differentiate into plasma cells in response to IL-10 but not IL-4. *Int Immunol* 1995;**7**:1243-53.

82 O'Nions J, Allday MJ. Proliferation and differentiation in isogenic populations of peripheral B cells activated by Epstein-Barr virus or T cell-derived mitogens. *J Gen Virol* 2004;**85**:881-95.

83 Liu F, Dai S, Feng D, Qin Z, Peng X, Sakamuri S, et al. Distinct fate, dynamics and niches of renal macrophages of bone marrow or embryonic origins. *Nat Commun* 2020;**11**:2280.

84 Tanaka Y, Koyanagi Y, Tanaka R, Kumazawa Y, Nishimura T, Yamamoto N. Productive and lytic infection of human CD4+ type 1 helper T cells with macrophage-tropic human immunodeficiency virus type 1. *J Virol* 1997;**71**:465-70.

85 Jung U, Foley JE, Erdmann AA, Eckhaus MA, Fowler DH. CD3/CD28-costimulated T1 and T2 subsets: differential in vivo allosensitization generates distinct GVT and GVHD effects. *Blood* 2003;**102**:3439-46.

86 Pilat N, Mahr B, Gattringer M, Baranyi U, Wekerle T. CTLA4Ig Improves Murine iTreg Induction via TGFβ and Suppressor Function In Vitro. *J Immunol Res* 2018;**2018**:2484825.

87 Blinova VG, Novachly NS, Gippius SN, Hilal A, Gladilina YA, Eliseeva DD, et al. Phenotypical and Functional Characteristics of Human Regulatory T Cells during Ex Vivo Maturation from CD4+ T Lymphocytes. *Applied Sciences* 2021;**11**:5776.

88 Al-Shanti N, Aldahoudi Z. Human purified CD8+ T cells: Ex vivo expansion model to generate a maximum yield of functional cytotoxic cells. *Immunol Invest* 2007;**36**:85-104.

89 Nhung HTM, Anh BV, Huyen TL, Hiep DT, Thao CT, Lam PN, et al. Ex vivo expansion of human peripheral blood natural killer cells and cytotoxic T lymphocytes from lung cancer patients. *Oncol Lett* 2018;**15**:5730-8.

90 Zhang J, Pippin JW, Krofft RD, Naito S, Liu ZH, Shankland SJ. Podocyte repopulation by renal progenitor cells following glucocorticoids treatment in experimental FSGS. *Am J Physiol Renal Physiol* 2013;**304**:F1375-89.

91 Verma R, Venkatareddy M, Kalinowski A, Li T, Kukla J, Mollin A, et al. Nephrin is necessary for podocyte recovery following injury in an adult mature glomerulus. *PLoS One* 2018;**13**:e0198013.

92 Liao R, Liu Q, Zheng Z, Fan J, Peng W, Kong Q, et al. Tacrolimus Protects Podocytes from Injury in Lupus Nephritis Partly by Stabilizing the Cytoskeleton and Inhibiting Podocyte Apoptosis. *PLoS One* 2015;**10**:e0132724.

93 Shim G, Lee S, Han J, Kim G, Jin H, Miao W, et al. Pharmacokinetics and in vivo fate of intra-articularly transplanted human bone marrow-derived clonal mesenchymal stem cells. *Stem Cells Dev* 2015;**24**:1124-32.

94 Lee RH, Pulin AA, Seo MJ, Kota DJ, Ylostalo J, Larson BL, et al. Intravenous hMSCs improve myocardial infarction in mice because cells embolized in lung are activated to secrete the anti-inflammatory protein TSG-6. *Cell Stem Cell* 2009;**5**:54-63.

95 Sokal EM, Lombard CA, Roelants V, Najimi M, Varma S, Sargiacomo C, et al. Biodistribution of Liver-Derived Mesenchymal Stem Cells After Peripheral Injection in a Hemophilia A Patient. *Transplantation* 2017;**101**:1845-51.

96 Gholamrezanezhad A, Mirpour S, Bagheri M, Mohamadnejad M, Alimoghaddam K, Abdolahzadeh L, et al. In vivo tracking of 111In-oxine labeled mesenchymal stem cells following infusion in patients with advanced cirrhosis. *Nucl Med Biol* 2011;**38**:961-7.

97 Couto BG, Goldenberg RC, da Fonseca LM, Thomas J, Gutfilen B, Resende CM, et al. Bone marrow mononuclear cell therapy for patients with cirrhosis: a Phase 1 study. *Liver Int* 2011;**31**:391-400.

98 Sood V, Mittal BR, Bhansali A, Singh B, Khandelwal N, Marwaha N, et al. Biodistribution of 18F-FDG-Labeled Autologous Bone Marrow-Derived Stem Cells in Patients With Type 2 Diabetes Mellitus: Exploring Targeted and Intravenous Routes of Delivery. *Clin Nucl Med* 2015;**40**:697-700.

99 Sherwin CM, Sagcal-Gironella AC, Fukuda T, Brunner HI, Vinks AA. Development of population PK model with enterohepatic circulation for mycophenolic acid in patients with childhood-onset systemic lupus erythematosus. *Br J Clin Pharmacol* 2012;**73**:727-40.

100 Lertdumrongluk P, Somparn P, Kittanamongkolchai W, Traitanon O, Vadcharavivad S, Avihingsanon Y. Pharmacokinetics of mycophenolic acid in severe lupus nephritis. *Kidney Int* 2010;**78**:389-95.

101 Joy MS, Hilliard T, Hu Y, Hogan SL, Dooley MA, Falk RJ, et al. Pharmacokinetics of mycophenolic acid in patients with lupus nephritis. *Pharmacotherapy* 2009;**29**:7-16.

102 Łuszczyńska P, Pawiński T, Kunicki PK, Durlik M, Augustyniak-Bartosik H, Hurkacz M. Pharmacokinetics of free and total mycophenolic acid in adult lupus nephritis patients-implications for therapeutic drug monitoring. *Eur J Clin Pharmacol* 2019;**75**:371-9.

103 Woillard JB, Bader-Meunier B, Salomon R, Ranchin B, Decramer S, Fischbach M, et al. Pharmacokinetics of mycophenolate mofetil in children with lupus and clinical findings in favour of therapeutic drug monitoring. *Br J Clin Pharmacol* 2014;**78**:867-76.

104 Bashar T, Apu MNH, Mostaid MS, Islam MS, Hasnat A. Pharmacokinetics and Bioavailability Study of a Prednisolone Tablet as a Single Oral Dose in Bangladeshi

Healthy Volunteers. *Dose Response* 2018;**16**:1559325818783932.
105 Booker BM, Magee MH, Blum RA, Lates CD, Jusko WJ. Pharmacokinetic and pharmacodynamic interactions between diltiazem and methylprednisolone in healthy volunteers. *Clin Pharmacol Ther* 2002;**72**:370-82.
106 Sagcal-Gironella AC, Sherwin CM, Tirona RG, Rieder MJ, Brunner HI, Vinks AA. Pharmacokinetics of prednisolone at steady state in young patients with systemic lupus erythematosus on prednisone therapy: an open-label, single-dose study. *Clin Ther* 2011;**33**:1524-36.
107 Wang HN, Zhu XY, Zhu Y, Xie QH, Lai LY, Zhao M, et al. The GSTA1 polymorphism and cyclophosphamide therapy outcomes in lupus nephritis patients. *Clin Immunol* 2015;**160**:342-8.
108 Joy MS, La M, Wang J, Bridges AS, Hu Y, Hogan SL, et al. Cyclophosphamide and 4-hydroxycyclophosphamide pharmacokinetics in patients with glomerulonephritis secondary to lupus and small vessel vasculitis. *Br J Clin Pharmacol* 2012;**74**:445-55.
109 Zickert A, Amoudruz P, Sundström Y, Rönnelid J, Malmström V, Gunnarsson I. IL-17 and IL-23 in lupus nephritis - association to histopathology and response to treatment. *BMC Immunol* 2015;**16**:7.
110 Sigdel KR, Duan L, Wang Y, Hu W, Wang N, Sun Q, et al. Serum Cytokines Th1, Th2, and Th17 Expression Profiling in Active Lupus Nephritis-IV: From a Southern Chinese Han Population. *Mediators Inflamm* 2016;**2016**:4927530.
111 Chun HY, Chung JW, Kim HA, Yun JM, Jeon JY, Ye YM, et al. Cytokine IL-6 and IL-10 as biomarkers in systemic lupus erythematosus. *J Clin Immunol* 2007;**27**:461-6.
112 Devarapu SK, Kumar Vr S, Rupanagudi KV, Kulkarni OP, Eulberg D, Klussmann S, et al. Dual blockade of the pro-inflammatory chemokine CCL2 and the homeostatic chemokine CXCL12 is as effective as high dose cyclophosphamide in murine proliferative lupus nephritis. *Clin Immunol* 2016;**169**:139-47.
113 Reynolds JA, McCarthy EM, Haque S, Ngamjanyaporn P, Sergeant JC, Lee E, et al. Cytokine profiling in active and quiescent SLE reveals distinct patient subpopulations. *Arthritis Res Ther* 2018;**20**:173.
114 Zhang CX, Cai L, Shao K, Wu J, Zhou W, Cao LF, et al. Serum IP-10 is useful for identifying renal and overall disease activity in pediatric systemic lupus erythematosus. *Pediatr Nephrol* 2018;**33**:837-45.
115 Girimaji N, Kaundal U, Rathi M, Sharma A, Nada R, Ramachandran R, et al. Regulatory B and T Cells and Their Association With Clinical Response in New-Onset Lupus Nephritis Patients. *Kidney Int Rep* 2020;**5**:1081-6.
116 Mashud R, Nomachi A, Hayakawa A, Kubouchi K, Danno S, Hirata T, et al. Impaired lymphocyte trafficking in mice deficient in the kinase activity of PKN1. *Sci Rep* 2017;**7**:7663.
117 Chernova I, Song W, Steach H, Hafez O, Al Souz J, Chen PM, et al. The ion transporter Na(+)-K(+)-ATPase enables pathological B cell survival in the kidney microenvironment of lupus nephritis. *Sci Adv* 2023;**9**:eadf8156.
118 Hiramatsu-Asano S, Sunahori-Watanabe K, Zeggar S, Katsuyama E, Mukai T, Morita Y, et al. Deletion of Mir223 Exacerbates Lupus Nephritis by Targeting S1pr1 in Fas(lpr/lpr) Mice. *Front Immunol* 2020;**11**:616141.

119 Gottschalk TA, Hall P, Tsantikos E, L'Estrange-Stranieri E, Hickey MJ, Hibbs ML. Loss of CD11b Accelerates Lupus Nephritis in Lyn-Deficient Mice Without Disrupting Glomerular Leukocyte Trafficking. *Front Immunol* 2022;**13**:875359.
120 Cassese G, Lindenau S, de Boer B, Arce S, Hauser A, Riemekasten G, et al. Inflamed kidneys of NZB / W mice are a major site for the homeostasis of plasma cells. *Eur J Immunol* 2001;**31**:2726-32.
121 Baudoux T, Husson C, De Prez E, Jadot I, Antoine MH, Nortier JL, et al. CD4(+) and CD8(+) T Cells Exert Regulatory Properties During Experimental Acute Aristolochic Acid Nephropathy. *Sci Rep* 2018;**8**:5334.
122 Saito S, Tsukaguchi N, Hasegawa T, Michimata T, Tsuda H, Narita N. Distribution of Th1, Th2, and Th0 and the Th1/Th2 cell ratios in human peripheral and endometrial T cells. *Am J Reprod Immunol* 1999;**42**:240-5.
123 Kitazawa J, Kimura F, Nakamura A, Morimune A, Hanada T, Amano T, et al. Alteration in endometrial helper T-cell subgroups in chronic endometritis. *Am J Reprod Immunol* 2021;**85**:e13372.
124 Schmidt T, Luebbe J, Kilian C, Riedel JH, Hiekmann S, Asada N, et al. IL-17 Receptor C Signaling Controls CD4(+) T(H)17 Immune Responses and Tissue Injury in Immune-Mediated Kidney Diseases. *J Am Soc Nephrol* 2021;**32**:3081-98.
125 Lin TJ, Wu CY, Tsai PY, Hsu WH, Hua KF, Chu CL, et al. Accelerated and Severe Lupus Nephritis Benefits From M1, an Active Metabolite of Ginsenoside, by Regulating NLRP3 Inflammasome and T Cell Functions in Mice. *Front Immunol* 2019;**10**:1951.
126 Humrich JY, Morbach H, Undeutsch R, Enghard P, Rosenberger S, Weigert O, et al. Homeostatic imbalance of regulatory and effector T cells due to IL-2 deprivation amplifies murine lupus. *Proc Natl Acad Sci U S A* 2010;**107**:204-9.
127 Kikuchi M, Wickman L, Rabah R, Wiggins RC. Podocyte number and density changes during early human life. *Pediatr Nephrol* 2017;**32**:823-34.
128 Nyengaard JR, Bendtsen TF. Glomerular number and size in relation to age, kidney weight, and body surface in normal man. *Anat Rec* 1992;**232**:194-201.
129 Oth T, Schnijderberg MC, Senden-Gijsbers BL, Germeraad WT, Bos GM, Vanderlocht J. Monitoring the initiation and kinetics of human dendritic cell-induced polarization of autologous naive CD4+ T cells. *PLoS One* 2014;**9**:e103725.
130 Pandiyan P, Hegel JK, Krueger M, Quandt D, Brunner-Weinzierl MC. High IFN-gamma production of individual CD8 T lymphocytes is controlled by CD152 (CTLA-4). *J Immunol* 2007;**178**:2132-40.
131 Ohshima Y, Yang LP, Avice MN, Kurimoto M, Nakajima T, Sergerie M, et al. Naive human CD4+ T cells are a major source of lymphotoxin alpha. *J Immunol* 1999;**162**:3790-4.
132 Agbanoma G, Li C, Ennis D, Palfreeman AC, Williams LM, Brennan FM. Production of TNF-α in macrophages activated by T cells, compared with lipopolysaccharide, uses distinct IL-10-dependent regulatory mechanism. *J Immunol* 2012;**188**:1307-17.
133 de Jong AJ, Pollastro S, Kwekkeboom JC, Andersen SN, Dorjée AL, Bakker AM, et al. Functional and phenotypical analysis of IL-6-secreting CD4(+) T cells in human adipose tissue. *Eur J Immunol* 2018;**48**:471-81.

134 Pan H, Huang W, Wang Z, Ren F, Luo L, Zhou J, et al. The ACE2-Ang-(1-7)-Mas Axis Modulates M1/M2 Macrophage Polarization to Relieve CLP-Induced Inflammation via TLR4-Mediated NF-κb and MAPK Pathways. *J Inflamm Res* 2021;**14**:2045-60.

135 Mohrs K, Wakil AE, Killeen N, Locksley RM, Mohrs M. A two-step process for cytokine production revealed by IL-4 dual-reporter mice. *Immunity* 2005;**23**:419-29.

136 Amarnath S, Dong L, Li J, Wu Y, Chen W. Endogenous TGF-beta activation by reactive oxygen species is key to Foxp3 induction in TCR-stimulated and HIV-1-infected human CD4+CD25- T cells. *Retrovirology* 2007;**4**:57.

137 Liu Z, Kuang W, Zhou Q, Zhang Y. TGF-β1 secreted by M2 phenotype macrophages enhances the stemness and migration of glioma cells via the SMAD2/3 signalling pathway. *Int J Mol Med* 2018;**42**:3395-403.

138 Xu R, Li Y, Yan H, Zhang E, Huang X, Chen Q, et al. CCL2 promotes macrophages-associated chemoresistance via MCPIP1 dual catalytic activities in multiple myeloma. *Cell Death Dis* 2019;**10**:781.

139 Vargas-Inchaustegui DA, Hogg AE, Tulliano G, Llanos-Cuentas A, Arevalo J, Endsley JJ, et al. CXCL10 production by human monocytes in response to Leishmania braziliensis infection. *Infect Immun* 2010;**78**:301-8.

140 Mogilenko DA, Kudriavtsev IV, Trulioff AS, Shavva VS, Dizhe EB, Missyul BV, et al. Modified low density lipoprotein stimulates complement C3 expression and secretion via liver X receptor and Toll-like receptor 4 activation in human macrophages. *J Biol Chem* 2012;**287**:5954-68.

141 Gulati P, Guc D, Lemercier C, Lappin D, Whaley K. Expression of the components and regulatory proteins of the classical pathway of complement in normal and diseased synovium. *Rheumatol Int* 1994;**14**:13-9.

142 Gonzalez-Suarez ML, Waheed AA, Andrews DM, Ascherman DP, Zeng X, Nayer A. Lupus vasculopathy: Diagnostic, pathogenetic and therapeutic considerations. *Lupus* 2014;**23**:421-7.

143 Reddy V, Martinez L, Isenberg DA, Leandro MJ, Cambridge G. Pragmatic Treatment of Patients With Systemic Lupus Erythematosus With Rituximab: Long-Term Effects on Serum Immunoglobulins. *Arthritis Care Res (Hoboken)* 2017;**69**:857-66.

144 Brown RP, Delp MD, Lindstedt SL, Rhomberg LR, Beliles RP. Physiological parameter values for physiologically based pharmacokinetic models. *Toxicol Ind Health* 1997;**13**:407-84.

145 Sun L, Akiyama K, Zhang H, Yamaza T, Hou Y, Zhao S, et al. Mesenchymal stem cell transplantation reverses multiorgan dysfunction in systemic lupus erythematosus mice and humans. *Stem Cells* 2009;**27**:1421-32.

146 Sun L, Wang D, Liang J, Zhang H, Feng X, Wang H, et al. Umbilical cord mesenchymal stem cell transplantation in severe and refractory systemic lupus erythematosus. *Arthritis Rheum* 2010;**62**:2467-75.

147 Wang D, Li J, Zhang Y, Zhang M, Chen J, Li X, et al. Umbilical cord mesenchymal stem cell transplantation in active and refractory systemic lupus erythematosus: a multicenter clinical study. *Arthritis Res Ther* 2014;**16**:R79.

148 Wang D, Zhang H, Liang J, Li X, Feng X, Wang H, et al. Allogeneic mesenchymal stem cell transplantation in severe and refractory systemic lupus erythematosus: 4 years

of experience. *Cell Transplant* 2013;**22**:2267-77.

149 Wang D, Akiyama K, Zhang H, Yamaza T, Li X, Feng X, et al. Double allogenic mesenchymal stem cells transplantations could not enhance therapeutic effect compared with single transplantation in systemic lupus erythematosus. *Clin Dev Immunol* 2012;**2012**:273291.

150 Ranjbar A, Hassanzadeh H, Jahandoust F, Miri R, Bidkhori HR, Monzavi SM, et al. Allogeneic adipose-derived mesenchymal stromal cell transplantation for refractory lupus nephritis: Results of a phase I clinical trial. *Curr Res Transl Med* 2022;**70**:103324.

151 Di Ianni M, Del Papa B, De Ioanni M, Moretti L, Bonifacio E, Cecchini D, et al. Mesenchymal cells recruit and regulate T regulatory cells. *Exp Hematol* 2008;**36**:309-18.
